# Structural heterogeneity and dynamics in the apical stem loop of s2m from SARS-CoV-2 Delta by an integrative NMR spectroscopy and MD simulation approach

**DOI:** 10.1093/nar/gkaf552

**Published:** 2025-06-30

**Authors:** Maria A Wirtz Martin, Joseph A Makowski, Tobias Matzel, Adam H Kensinger, Alexander Herr, Christian Richter, Hendrik R A Jonker, Anna Wacker, Jeffrey D Evanseck, Harald Schwalbe

**Affiliations:** Institute for Organic Chemistry and Chemical Biology, Center for Biomolecular Magnetic Resonance (BMRZ), Goethe University Frankfurt, Max-von-Laue-Str. 7, 60438 Frankfurt, Germany; Department of Chemistry and Biochemistry and Center for Computational Sciences, Duquesne University, Pittsburgh, PA 15282, United States; Institute for Organic Chemistry and Chemical Biology, Center for Biomolecular Magnetic Resonance (BMRZ), Goethe University Frankfurt, Max-von-Laue-Str. 7, 60438 Frankfurt, Germany; Department of Chemistry and Biochemistry and Center for Computational Sciences, Duquesne University, Pittsburgh, PA 15282, United States; Institute for Organic Chemistry and Chemical Biology, Center for Biomolecular Magnetic Resonance (BMRZ), Goethe University Frankfurt, Max-von-Laue-Str. 7, 60438 Frankfurt, Germany; Institute for Organic Chemistry and Chemical Biology, Center for Biomolecular Magnetic Resonance (BMRZ), Goethe University Frankfurt, Max-von-Laue-Str. 7, 60438 Frankfurt, Germany; Institute for Organic Chemistry and Chemical Biology, Center for Biomolecular Magnetic Resonance (BMRZ), Goethe University Frankfurt, Max-von-Laue-Str. 7, 60438 Frankfurt, Germany; Institute for Organic Chemistry and Chemical Biology, Center for Biomolecular Magnetic Resonance (BMRZ), Goethe University Frankfurt, Max-von-Laue-Str. 7, 60438 Frankfurt, Germany; Department of Chemistry and Biochemistry and Center for Computational Sciences, Duquesne University, Pittsburgh, PA 15282, United States; Institute for Organic Chemistry and Chemical Biology, Center for Biomolecular Magnetic Resonance (BMRZ), Goethe University Frankfurt, Max-von-Laue-Str. 7, 60438 Frankfurt, Germany

## Abstract

In structured RNAs, helical elements are often capped by apical loops that are integral structural elements, ranging from 3 to >20 nts of size on average, and display a highly heterogeneous energy landscape profile, rendering structural characterization particularly challenging. We here provide a characterization of the SARS-CoV-2 Delta s2m element containing a highly dynamic nonaloop using an integrative approach of nuclear magnetic resonance spectroscopy (NMR), small angle X-ray scattering (SAXS), and molecular dynamics simulations (MD). We further explored the conformational space in the s2m nonaloop and its transient closing 5′-G-U-3′ base pair by MD simulations weighted by experimental NMR observables, leading to a comprehensive representation of the s2m nonaloop motif. Our deconvolution of the ensemble into conformations and dynamics provides a basis for future ensemble-functional characterization of RNA structures featuring dynamic motifs.

## Graphical Abstract

**Graphical Abstract:**
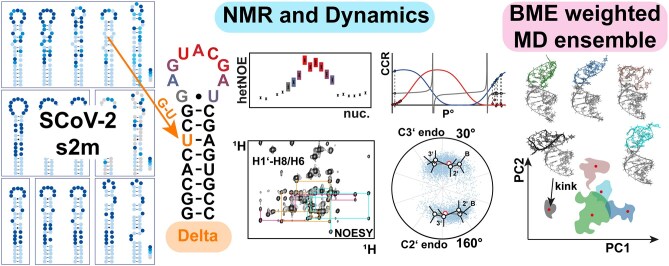

## Introduction

Even though the pivotal roles of RNAs in cellular processes are increasingly recognized [1], the number of experimental high-resolution three-dimensional RNA structures is sparse (<1%) compared to the number of protein structures that make up to 90% of deposited structures in the Protein Data Bank (PDB) [2]. This under-representation is partly caused by RNA’s inherent flexibility, which poses a substantial challenge for structure determination [3]. Nonetheless, understanding RNA dynamics and the formation of intricate higher-order structures is crucial for elucidating RNA function in diverse biological processes [4].

The emergence of the SARS-CoV-2 (SCoV-2) virus initiated an explosive increase in RNA-related research [5]. The coronavirus positive-sense single-stranded-RNA genome with ∼30 kilobases is one of the largest RNA viral genomes [6]. It exhibits conserved structural elements, especially in its 5′ and 3′ genomic ends and in the frameshift region [7]. Secondary structure predictions of these elements were computed early in the pandemic [8]. In August 2020, the secondary structures of 15 *cis-*acting elements within these regions were determined by NMR spectroscopy (NMR) and cross-validated by dimethyl sulfoxide (DMS) foot-printing [9].

The 3′-untranslated region (3′-UTR) of SCoV-2 contains the stem loop 2 motif (s2m), a highly conserved RNA element [7]. Being highly conserved, it is, however, part of the hypervariable region (HVR) (Rfam: RF00164), which diverges significantly between different viruses. S2m was first described in human astroviruses in 1997 as a 41 nucleotide (nt) long structured region [10]. Phylogenetic analyses have since confirmed the preservation of s2m in *astroviridae*, *calciviridae*, *picornaviridae*, and *coronaviridae*, all distantly related virus families [11, 12]. The phylogenetic distribution of s2m suggests that this element could be transferred from virus to virus as a mobile element [13–15].

Within the RNA genome of SARS-CoV-1 (SCoV-1), s2m was the only isolated RNA element for which a 2.7 Å resolution X-ray crystal structure was solved [16]. This structure revealed a 90° kink between the upper and the lower helical stems of s2m. Within this kink region, a binding site for two magnesium ions (Mg^2+^) was detected. The observation of the GNRA-like (N: any nucleotide, R: either guanine or adenine) pentaloop and the unique fold of the SCoV-1 s2m element led to the hypothesis that s2m could potentially mimic the ribosomal 530 loop, hijacking host protein synthesis [16].

**Figure 1.**
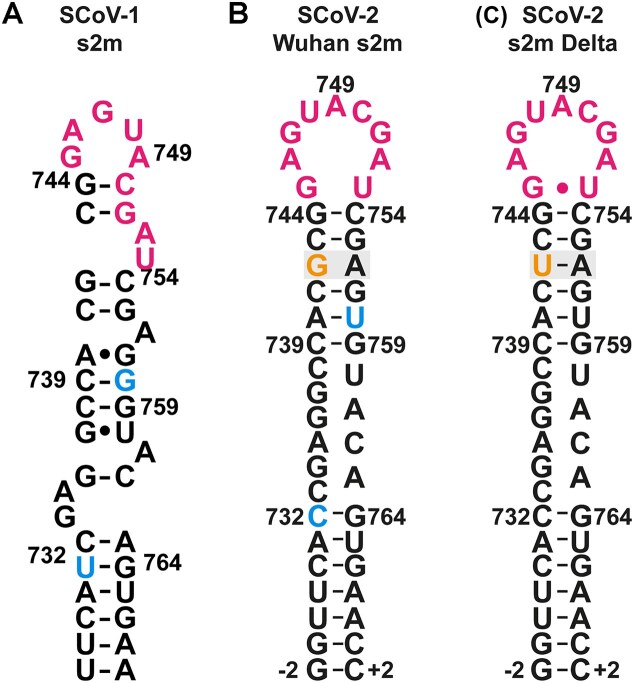
Secondary structures of s2m in SCoV-1 (**A**) [16], SCoV-2 Wuhan (**B**) [9], and SCoV-2 Delta (**C**) [17]. Highlighted in pink are the nucleotides that form a nonaloop with a transient G-U base pair in SCoV-2 Delta. Highlighted in blue are the mutated nucleotides from SCoV-1 to SCoV-2: U732-C732; G758-U758. Highlighted in orange are the mutated nucleotides from SCoV-2 Wuhan to SCoV-2 Delta: G742-U742. Nucleotide numbering corresponds to the position in the genome -29 000.

In SCoV-2, two mutations in s2m induced a change in secondary structure compared to SCoV-1 s2m [9]. The initial SCoV-2 s2m secondary structure from the wild-type variant of the virus (hereafter called Wuhan s2m) has two stem regions separated by an internal loop of 10 nt involving nucleotides C29,733 to C29,738 on the 5′-terminal side and U29,760 to A29,763 on the 3′-terminal side, respectively, and an apical nonaloop comprising G29,745 to U29,753 instead of a pentaloop. In SCoV-2 Delta, s2m acquired an additional mutation in the upper stem where G29,742 (G742) was mutated to a uridine, allowing for a stabilizing A-U base pair instead of a G-A mismatch (Fig. 1). Extensive and thorough NMR investigations were performed for this RNA resulting in complete assignment and characterization of its dynamic properties [17].

It has been shown that the loop of SCoV-2 s2m contains a palindromic sequence leading to a Mg^2+^-dependent formation of kissing-loop dimers *in vitro*. Further, s2m was suggested to impact the miR-1307-3p regulation pathway through binding of this microRNA (miRNA) [18, 19]. A subsequent MD study modelled the three-dimensional structure and dynamics based on NMR data previously described [9] and conducted a comparison to the SCoV-1 crystallographic structure (PDB: 1XJR) [20]. This study revealed the thermodynamic consequences of the highly dynamic SCoV-2 s2m RNA compared to the SCoV-1 s2m RNA since SCoV-1 has a stable GNRA-like pentaloop in contrast to the nonaloop with a transient closing 5′-G-U-3′ base pair in SCoV-2 s2m RNA [21], rationalizing experimentally observed differences in kissing dimer formation. However, later genotypes of s2m in the Omicron variant contain only a truncated remnant of s2m [22], thus inspiring several studies that showed its dispensability for the virus [23].

In fact, despite experimental evidence delineating which nucleotides are involved in base pairing, many different secondary structures have been proposed and published for SCoV-2 s2m. These models for the secondary structure of s2m were based on *in vitro* as well as *in vivo* methods including homology models [14, 21, 24], bioinformatic analysis [7, 25], sequencing [26], SHAPE [27–30], RNA–RNA interaction studies [31, 32], and DMS footprinting [17, 27, 33]. In the work presented here, we discuss the results of these studies that report on the SCoV-2 secondary structure in light of the NMR experimental structure. By assessing the cleavage activity of the viral endonuclease, SCoV-2 Nsp15 prefers unstructured uridine residues as target sites [34], as present in the loop of s2m we also show how erroneous secondary structure predictions can lead to the wrong prediction of cellular function.

The still undisputable prevalence of s2m in several virus families, although not highly conserved in terms of primary structure, warrants the characterization of its three-dimensional structure and dynamics, as these are essential for understanding its function. As promising target sites for low molecular weight inhibitors, three-dimensional loop structures are of great pharmaceutical interest, yet remain relatively undefined due to the timescale and complexity linked to their dynamic nature [35–38]. To overcome these limitations, we integrate NMR spectroscopy with molecular dynamics (MD) simulations, leveraging experimental data to refine the structural ensembles from simulation, capturing the conformational heterogeneity of s2m with enhanced accuracy. This approach allows us to resolve dynamic structural features that would be difficult to characterize through either method alone. With our three-dimensional ensemble structure of s2m, we extend the knowledge base for structurally dynamic RNA apical loops and contribute to understanding of complex RNA folding pathways involving apical loops as structure formation hubs.

## Materials and methods

### DNA template preparation

DNA plasmids coding for s2m Delta_short (sequence listed in Supplementary Table S1) were prepared via cloning of hybridized complementary oligonucleotides into EcoRI and NcoI restriction sites of the pSP64 vector (Promega). This plasmid contains the HDV ribozyme sequence and was kindly provided by Prof. Dr. Julia Weigand within work conducted in the COVID-19 NMR consortium. DNA amplification and purification was performed as described before [9, 17]. Plasmid vector cards will be provided upon request. Further DNA sequences (SCoV-1, SCoV-2 Wuhan s2m, and SCoV-2 s2m Delta) in plasmid vectors are given in Supplementary Table S1 as well. All nucleotide numbering in text and figures is reduced by −29 000 for all s2m RNA sequences described in this study.

### *In vitro* transcription and RNA purification

*In vitro* transcriptions for RNA sample preparation were performed as published before [9, 17]. The RNAs were folded by heating to 95°C for 5 min and cooling down at room temperature. To ensure the monomeric state of the RNA, native gel electrophoresis was performed with an RNA concentration of 500 pmol per sample (diluted 1:5) and visualized under UV/Vis spectroscopy. The RNA samples used for NMR and their labelling schemes are described in Supplementary Table S2. The RNA samples used for the Nsp15 endoribonuclease assay are summarized in Supplementary Table S3.

### Nsp15 protein production, purification, and cleavage assay

Nsp15 protein production and purification were performed as described before [39]. Endoribonuclease cleavage assay was performed with an [RNA]:[protein] ratio of 5:1 and incubated for 1, 15, and 60 min at 25°C or 37°C, with and without MnCl_2_. In addition, samples were incubated with denatured Nsp15 for a period of 240 min to test for the absence of RNAse. For this purpose, the protein was heated to 95°C for 5 min in advance. The reaction was stopped by adding ethylenediaminetetraacetic acid (EDTA) with a final concentration of 20 mM. Analysis of cleavage products was performed through denaturing polyacrylamide gel electrophoresis (PAGE). PAGE was performed on 15% polyacrylamide (PAA) gels in 1× TBE buffer (90 mM Tris base, 90 mM boric acid, 2 mM EDTA, pH 8) at 260 V for 30 min. The individual gels were loaded separately according to RNA construct with 2 μl of the corresponding sample per chamber (10 pmol RNA). A reference sample of each construct was also applied. The assay was performed in endonuclease assay buffer (25 mM K_2_HPO_4_/KH_2_PO_4_ pH 7.5; 300 mM NaCl; 5 mM MgCl_2_; 5 mM DTT) with and without additional 2 mM MnCl_2_. To visualize the RNA on the gels, they were each stained in GelRed^™^ (Biotium, USA) solution for 10 min and observed under UV light.

### NMR experiments

A list of all measured NMR experiments with parameters can be found in Supplementary Tables S4–6. Spectra were measured at 600, 800, and 900 MHz Bruker Avance (III/ NEO) NMR spectrometers equipped with 5 mm cryogenic triple resonance TCI-N probes and at 700 MHz Bruker Avance (III) spectrometer with a QCI-^31^P probe and 800 MHz with TXO cryogenic probe. DSS (sodium trimethylsilylpropanesulfonate) was used as an external sample reference. All RNA samples were measured in 25 mM K_2_HPO_4_/KH_2_PO_4_, 50 mM KCl, pH 6.2. Analysis of NMR spectra was performed using TopSpin 4.2.0 and NMRFAM-SPARKY [40] for the assignment of resonance chemical shifts. The chemical shifts of s2m Delta_short at 283 K, 298 K, and 308 K are deposited in the BMRB with the ID: 34919.

### ARIA structure calculation

Structure calculations were performed on the s2m Delta_short RNA construct. NOE cross peaks from 2D ^1^H, ^1^H-NOESY spectra (Supplementary Table S6) with 50, 100, and 150 ms mixing time (sample#7) and from a 4D HMQC-NOESY-HMQC (Supplementary Table S6) with a mixing time of 150 ms were used to obtain ^1^H–^1^H distance restraints. For structure calculations with ARIA 1.2 (custom webportal implementation based on [41]), partially assigned NOESY peak lists, resonance lists, standard canonical A-form RNA dihedral values for α, β, γ, δ, ε, and ζ as well as the glycosidic torsion angle χ, H-bond restraints from confirmed hydrogen Watson–Crick base pairs and weak base-planarity restraints were used. A summary of experimental restraints and further constraints is given in Supplementary Tables S7–9. For loop residues, loop closing base pairs and stem loop terminal base pairs, dihedral angle values were not restrained. 20 structures with 100 structures per iterations and 200 structures in the last cycle were calculated using the standard protocols and settings for RNA structure calculations. Water refinement was performed for the final bundle of lowest-energy structures using optimized potentials for liquid simulation (OPLS [42]) charges and nonbonded parameters. The hydrodynamic radius and rotational correlation time *τ*_c_ was estimated using HydroNMR [43]. Assessment of the NMR solution structure quality and restraints of s2m Delta_short were performed using the standard analysis scripts, 3DNA [44], Molprobity [45], and validated by the PDB (ID: 9FM4).

### Heteronuclear {^1^H}-^13^C NOEs

Measurements were carried out with sample#4.5 at 800 MHz and 308 K. NOE and reference experiments were measured in an interleaved manner using a pseudo 3D ^1^H,^13^C HSQC (Supplementary Table S6) with a relaxation delay of 5 s and a presaturation delay of 3 s. An off-resonance pulse at −1000 ppm was applied in the reference experiment for temperature compensation. Heteronuclear NOE (hetNOE) values were calculated based on signal intensities and errors (${{\Delta }_{hetNOE}}$) were calculated based on the signal/noise ratio in the spectra according to the following equation:


(1) \begin{eqnarray*}
{{\Delta }_{{\mathrm{hetNOE}}}} = \sqrt {{{{\left( {\frac{{{{{\mathrm{I}}}_{{\mathrm{NOE}}}}}}{{{\mathrm{I}}_{{\mathrm{ref}}}^2}} \times {{{\mathrm{N}}}_{{\mathrm{ref}}}}} \right)}}^2} + {{{\left( {\frac{1}{{{{{\mathrm{I}}}_{{\mathrm{ref}}}}}} \times {{{\mathrm{N}}}_{{\mathrm{NOE}}}}} \right)}}^2}}
\end{eqnarray*}

- *I*_NOE_: Intensity of NOE spectrum
- *I*_ref_: Intensity of noNOE spectrum (reference spectrum)
- *N*_ref_: Noise in noNOE spectrum (reference spectrum)
- N_NOE_: Noise in NOE spectrum

### Determination of overall global correlation time from heteronuclear relaxation data

The overall global correlation time was determined using *T*_1_, *T*_1ρ_, and hetNOE data (600 and 800 MHz) from aromatic (C6H6/C8H8) spectra recorded at 308 K (Supplementary Table S6). *T*_1_ and *T*_1ρ_ analysis was performed using NMRFAM-SPARKY [40] from fitting of peak heights with a monoexponential two-parameter function. HetNOE values at 600 MHz were derived as described above. *R*_2_ values were obtained from R_1_ρ relaxation decays following the equation in (2) and (3) with a spin lock field strength power of 2000 Hz. *R*_1_, *R*_2_, and hetNOE values were then fitted using Modelfree Relax GUI software [46, 47] to obtain an averaged spin specific global correlation time. The mean value *τ*_c_ = 3.898 ± 0.56 ns from best fitting models (tm0-tm9) was used as global correlation time of s2m Delta_short RNA.


(2) \begin{eqnarray*}
{{{\mathrm{R}}}_2} = \frac{{{{{\mathrm{R}}}_{1\rho }} - {{{\mathrm{R}}}_1}{{{\cos }}^2}\theta }}{{{{{\sin }}^2}\theta }}
\end{eqnarray*}

- θ: Angle of the effective spin-lock field with *B*_0_ field, determined by (3)
  (3) \begin{eqnarray*}
\theta = {{\tan }^{ - 1}}\left( {\frac{\nu }{\Omega }} \right)
\end{eqnarray*}
- ν: Spin-lock field strength, in Hertz
- Ω: Offset of the nucleus resonance spin-lock carrier, in Hertz

### Determination of ribofuranosyl ring conformation by pseudorotation phase (*P*) and determination of the glycosidic angle χ

The calculation of the sugar pucker conformation based on ^13^C chemical shift assignment was carried out as published before [48, 49]. The obtained canonical coordinates can1 and can2 are representative for population of a conformation with a single pseudorotation phase (*P*) and exocyclic torsion angle (γ). The pseudorotation phase was also determined using ^3^*J* coupling constants (H1’-H2’, H2’-H3’, H3’-H4’) derived from a forward (*fw*)-HCC-TOCSY-CCH-E.COSY experiment [50, 51] measured with sample#10 at 308 K and 700 MHz (Supplementary Table S6). Analysis of the NMR spectrum was performed visually by determining the distance in Hz of coupled peaks, manually by extracting 2D planes of coupled peaks and overlaying them to obtain a shift and through a previously published script [52]. A mean value was then calculated. The ^3^*J* (H, H) coupling constants were fitted to Karplus parametrization curves [53]. Further, a quantitative 2D Γ-HCCH and 3D *fw*-Γ-HCCH experiment [54] were measured with sample#10 at 308 K at 700 MHz (Supplementary Table S6). Dipole (^1^H,^13^C)-dipole (^1^H,^13^C) cross-correlated-relaxation rates (CCR) $\Gamma _{{\mathrm{C}}1\prime {\mathrm{{\rm H}}}1\prime {\mathrm{C}}2\prime {\mathrm{H}}2\prime }^{{\mathrm{DD}},{\mathrm{DD}}}$ and $\Gamma _{{\mathrm{C}}3\prime {\mathrm{{\rm H}}}3\prime {\mathrm{C}}4\prime {\mathrm{H}}4\prime }^{{\mathrm{DD}},{\mathrm{DD}}}$ were determined and fitted to theoretical curves as described in Felli *et al.* [54]. The global rotational correlation time τ_C_ for calculation of parametrization curves was determined using Modelfree Relax GUI software [46, 47] from hetNOE-values and *T*_1_ and *T*_1ρ_ relaxation decays as described above. The C3’-endo population distribution was estimated by globally fitting the averaged experimental rates to a two-state model. Pseudoratation phases of 18° and 162° for C3’-endo and C2’-endo, respectively, were used. For different percentages of C3’-endo conformation (1%–100%) the rates were calculated and subtracted from the experimental rates. The minima of the resulting curves were interpreted as the percentage of nucleotides that adopt a C3’-endo conformation. Derivation of C3’-endo population was performed with CCR rates and ^3^*J* (H, H) coupling constant data separately.

The relative orientation of the ribose and the nucleobase was derived from the glycosidic angle χ. A quantitative Γ-HCN experiment [55] was measured with sample#10 at 308 K and 600 MHz (Supplementary Table S6). Dipole(H1’-C1’)-dipole(H6/8-C6/8)-CCR rates $\Gamma _{{\mathrm{C}}1\prime {\mathrm{{\rm H}}}1\prime {\mathrm{C}}6{\mathrm{H}}6}^{{\mathrm{DD}},{\mathrm{DD}}}$ and $\Gamma _{{\mathrm{C}}1\prime {\mathrm{{\rm H}}}1\prime {\mathrm{C}}8{\mathrm{H}}8}^{{\mathrm{DD}},{\mathrm{DD}}}$ were obtained and fitted to theoretical curves to obtain the χ angle.

### SAXS sample preparation

RNA samples for small angle X-ray scattering (SAXS) were prepared as described in methods (*in vitro* transcription and RNA purification) at concentrations of 1.5 and 2.5 mg/ml (corresponding to 181 and 302 μM) in SAXS buffer (50 mM Bis-Tris, 25 mM NaCl, pH 6.2). The filtrates from centrifugal buffer exchange were used as buffer blank for background scattering subtraction.

### SAXS data acquisition and initial processing

Synchrotron SAXS data were collected in batch mode on the EMBL P12 BioSAXS beamline at the PETRA III storage ring (DESY, Hamburg, Germany) [56] using a Pilatus 6 M 2D photon detector at a sample-detector distance of 3.0 m and a wavelength of *λ* = 0.124 nm. Data were measured at 298 K at continuous flow with a total exposure time of 3.135 s (33 × 95 ms frames). Automated processing was performed at the beamline, consisting of scattering data normalization to the transmitted beam intensity, radial averaging to yield 1D scattering curves and subtraction of the respective buffer scattering data.

### SAXS data analysis

SAXS data were processed with ATSAS 4.0.0-2 [57] and BioXTAS RAW 2.3.0 [58]. The 1D scattering curve of the 1.5 and 2.5 mg/ml sample was plotted as log(*I*(*q*)) versus *q*, where *q* is defined as:


(4) \begin{eqnarray*}
{\mathrm{q}} = 4\sin \theta /2\lambda
\end{eqnarray*}

In (4), θ is the scattering angle and λ is the X-ray wavelength.

For compatibility with downstream processing in BioXTAS RAW [58], λ was given in Å. The complete set of parameters according to the guidelines defined by Trewhella *et al.* [59] are provided in Supplementary Table S10.

The *R*_g_ of s2m Delta_short from SAXS scattering curves were determined by Guinier analysis and the molecular weight by the Bayesian interference model [60]. The *R*_g_ and *D*_max_ values were obtained for ARIA structure models using ATSAS software [57]. *Abinitio* model building was performed with DAMMIN [61] from 1.5 mg/ml sample and ensemble members from NMR ARIA structure calculation were aligned using CIFSUP from BioXTAS RAW [58]. Electron density maps were reconstructed with DENSS [62] from the GNOM-derived [57] pair distance distribution function and visualized using PyMOL, v2.6.0, Schrödinger LLC. SAXS data were deposited to the SASBDB with the ID: SASDUD9 (1.5 mg/ml) and SASDXB3 (2.5 mg/ml). Integrated low resolution models are visualized as movies in Supplementary Data (mov1 and mov2).

### NMR ensemble comparison with SAXS

Back-calculated scattering profiles of ARIA-derived structure models were compared with experimental scattering data using the online CRYSOL webserver [63] and ATSAS 4.0.0-2 [57]. χ² values between NMR-derived structures and SAXS data were calculated. To assess the impact of low-*q*-region artifacts on χ² values, the first 50 points of the scattering curves were discarded (low *q*-data). Furthermore, the small angle data (first 500 points) measured at low concentration were merged with the high angle data at higher concentration to obtain a merged scattering curve to reduce the contribution of inter-particle repulsion [64]. The members of the NMR ensemble members derived from the ARIA structure calculation were modified to each contain a 5′ triphosphate and a 3′ cyclic phosphate. At this stage, 10 out of 20 final structures were selected by filtering against SAXS data. In detail, each state was fitted to the experimental scattering data given in Supplementary Table S11, and the states were sorted according to χ² values derived from CRYSOL. Predicted SAXS scattering curves from ARIA structure models were calculated for each model and averaged over 20 or the 10 best models.

### Molecular dynamics simulations

MD simulation data were collected through 20 simulations, each initiated from a distinct ARIA structure [65]. All simulations employed the AMBER force field with the ff99_χ_OL3 parameter set through the NAMD MD engine [66–70]. To address known issues in AMBER nucleic acid parameterization for 5′ terminal phosphate capping, the ARIA structures were deprotonated at the 5′-end and used modified charge parameters, resulting in a net charge of −25 for the simulated RNA [71]. Systems were solvated with 15 Å padding of TIP3P waters with periodic conditions and charge neutralized through the addition of 25 K^+^ with Li/Merz parameters, resulting in an approximate concentration of 130 mM K^+^ [72, 73]. We simulated the system at 310 K under the NPT ensemble, consistent with our prior MD studies and comparable with experimental NMR conditions [20, 74–76]. The systems were subjected to 1000 steps of conjugate gradient energy minimization and equilibrated for 50 ns such that the potential energy and volume had stabilized prior to collecting data for analysis. Each of the 20 production simulations were carried out for 1 μs, for a total of 20 μs of aggregated simulation data.

### Principal component analysis

Concatenated simulated data from the 20 simulations were used to compute covariances for principal component analysis (PCA), wherein highly dimensional data are projected to a much lower-dimensional linear subspace capturing the largest fraction of variance in the data, as indicated by a scree plot [77, 78]. This procedure ultimately yields a depiction of molecular structural information separated by relative similarity and dissimilarity [79]. The index of selectivity used for PCA was based on the coordinate covariances of the terminal loop (nt. 8–18) nonhydrogen atoms, where ∼50% of coordinate variance was usually captured within two principal components (PCs) or 90% within 20 components. A 10 component Gaussian Mixture Model (GMM) was used to estimate the density of sampling in PC space through expectation maximization, allowing for identification of frequently visited regions of conformational space [80, 81]. The GMM density estimate was further used to approximate the underlying relative free energy Δ*F* via the relationship


(5) \begin{eqnarray*}
\Delta {\mathrm{F}} = - kT\ln \left( {{\mathrm{P}}/{{{\mathrm{P}}}_{\max }}} \right)
\end{eqnarray*}

where *k* is the Boltzmann constant, *T* is the simulated temperature, and *P* is the (marginal) probability of a given coordinate in configuration space [82, 83]. Subsequently, *k-*means clustering was used to partition simulated structures into conformational substates (CSs). Through minimizing the within-clusters sum of squares *J* given by


(6) \begin{eqnarray*}
{\mathrm{J}} = \sum _{i = 1}^{\mathrm{k}}{{\sum }_{{\mathrm{x}} \in {{C}_i}}}||\mathrm{x} - {{\mu }_i}||_2^2
\end{eqnarray*}

where *C_i_* denotes the set of points assigned to cluster *i* with centroid ${{\mu }_i}$, *k*-means produces clusters that minimize the distance to each centroid, maximizing similarity within clusters [84, 85]. Through optimization, a representative centroid structure was determined for each CS. The number of clusters *k* was selected to yield CSs with centroids as consistent as possible with free energy basin minima estimated by equation (5) [85]. PCA, GMM optimization, and clustering used the Scikit-learn Python library [86]. Leontis-Westhof annotated secondary structures were generated for each CS centroid using Barnaba [87, 88]. Molecular visualization was done with VMD [89].

### Bayesian maximum entropy reweighting

The Bayesian maximum entropy (BME) reweighting method was employed to construct a conformational ensemble of a biomolecular system by integrating molecular simulations and experimental data [90]. The BME cost function *L* is given as follows:


(7) \begin{eqnarray*}
L = \frac{m}{2}{{\chi }^2}({\mathrm{w}}) - \theta {{{\mathrm{S}}}_{{\mathrm{rel}}}}({\mathrm{w}})
\end{eqnarray*}

In (7), *m* is the number of observables, *w* is the vector of weights with dimensionality equal to the number of frames of simulation data, ${{\chi }^2}$ measures the agreement with experimental means, the relative entropy *S*_rel_ quantifies the difference between the optimized weights and the initial, uniform weighting, and *θ* the “temperature” hyperparameter used to modulate the contribution of the relative entropy to optimization. The cost function (7) balances the ${{\chi }^2}$ deviation between experimental observables (NOE, ^3^*J*, or CCR) and their simulated counterparts with an entropy term, scaled by *θ*, that penalizes excessive deviation from the original ensemble. The resulting reweighting assigns each frame from the original MD simulation a new probability, ensuring that reweighted observables better match experiment while maintaining maximal entropy. Interprotonic NOE distances were computed from trajectories using MDTraj, and ^3^*J* scalar couplings and CCRs were computed using an in-house Python script [54, 91]. We fitted the simulations against experimental NOEs, ^3^*J* couplings, and CCRs using the BME optimization as implemented in the BME Python library [90]. The hyperparameter *θ* was determined through 5-fold cross-validation for each set of observables (*θ*_NOE_ = 0.3; *θ*_CCR_ = 3; *θ*_3J_ = 5), ensuring that the resultant reweighting did not overfit to the experimental means. Cross-validation was not carried out when reweighting according to all NMR observables simultaneously due to software limitations, so we selected *θ*_ALL_ = 10 yielding 13% effective frames, enough for structural interpretation. To generate structural ensembles reflective of the reweighted probabilities, we sampled 50 000 out of 1 000 000 structures from 20 μs of MD data with replacement using the weights from BME reweighting with the NumPy Python library, producing refined ensembles corresponding to each observable [92].

## Results and discussion

### The secondary structure of s2m is retained in the context of the 3′ terminal hypervariable region of the SARS-CoV-2 genome

The NMR characterization of s2m started with the original sequence (in this study named Wuhan s2m) (Supplementary Table S1). A divide-and-conquer approach confirmed the conservation of the Wuhan s2m secondary structure in the context of the entire 3′-UTR and its substructure HVR (Supplementary Fig. S1) [9, 17]. While the imino spectrum of the entire 3′-UTR of SCoV-2 containing 337 nt was extremely crowded and did not resolve all signals of Wuhan s2m, the imino spectrum of the smaller HVR, on the other hand, showed almost perfect overlap of the NMR signatures with those of the corresponding Wuhan s2m signature.

The SCoV-2 RNA genome of the Delta variant acquired a mutation from G742 to U742. Instead of the G-A mismatch in the upper stem, the mutation in s2m Delta led to the formation of a new A-U base pair in the upper stem, as previously observed by us [17, 76]. Overall, the secondary structure of s2m Delta remained very similar to the NMR-derived secondary structure of Wuhan s2m. The G to U mutation reduced the conformational dynamics of Wuhan s2m, allowing us to reach >90% of chemical shift assignment of s2m Delta [17].

In addition to its highly dynamic nonaloop, the internal loop of s2m Delta (5′-C733-C738-3′ and 5′-U760-A763-3′) features significant dynamics [17], complicating determination of its solution structure by NMR. Here, we focused on solving the three-dimensional structure of the upper stem including the apical loop to describe the structural characteristics of this RNA sub-element, referred to as s2m Delta_short in this study.

### NMR secondary structure determination of s2m delta_short

**Figure 2.**
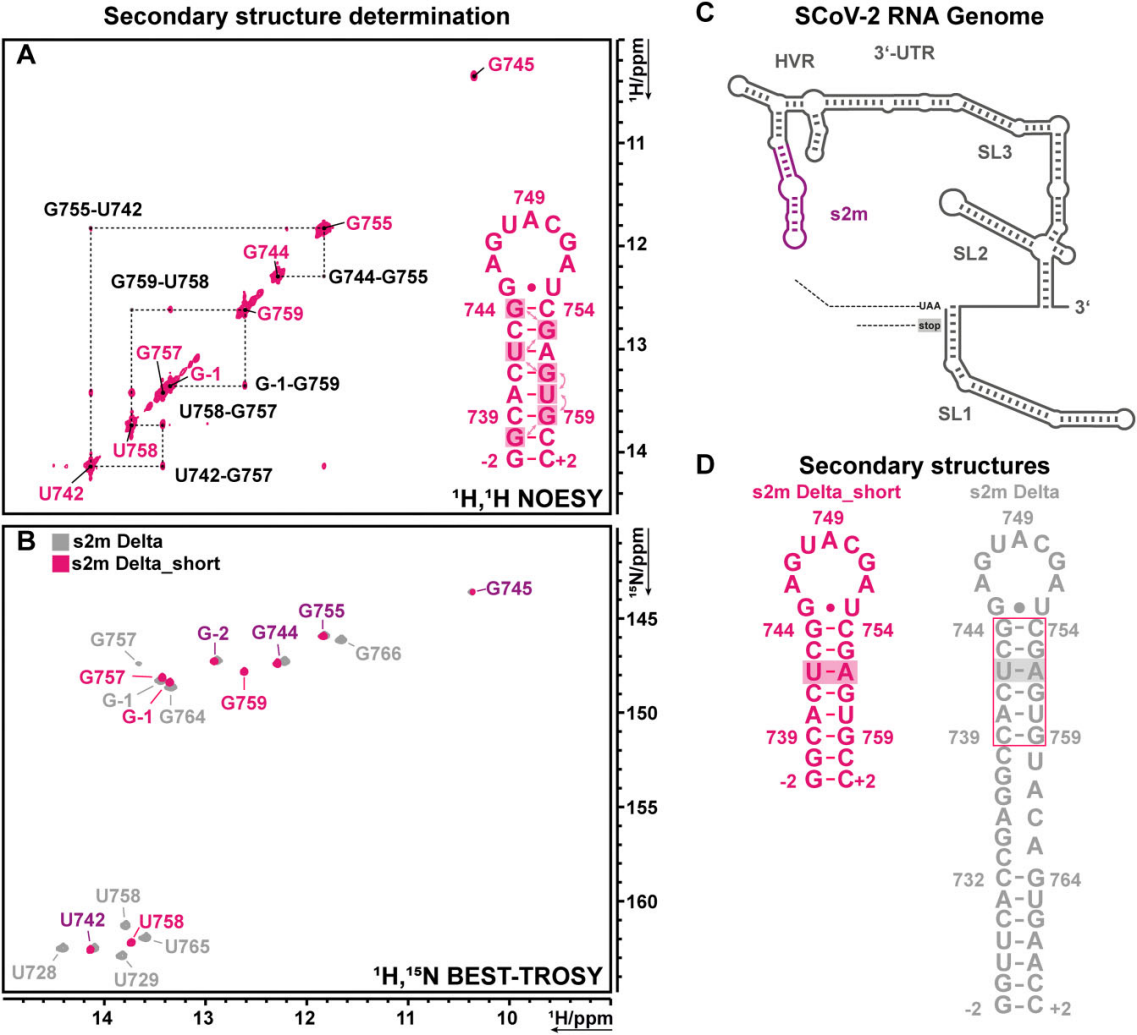
Secondary structure determination and imino proton assignment of s2m Delta_short. (**A**) ^1^H, ^1^H NOESY spectrum of the imino proton region with sequential walk GGUGUGG in black. (**B**) ^1^H,^15^N BEST-TROSY of the base paired region of s2m Delta (sample#2) and s2m Delta_short (sample#3). Nucleotide numbers in grey belong to the full-length construct, nucleotide numbers in magenta belong to the short construct that show a slight chemical shift perturbation, nucleotide numbers in violet belong to both constructs and overlap. (**C**) Schematic depiction of the 3′-UTR of SCoV-2 with s2m in the HVR highlighted in violet. (**D**) NMR-determined secondary structures of s2m Delta [17] and s2m Delta_short. Spectra were measured at 283 K.

The RNA sequence of the upper stem loop (s2m Delta_short) of s2m Delta comprises nucleotides 29 739 to 29 759 of the SCoV-2 RNA genome sequence (Fig. 2). We added two terminal G-C base pairs (G-2, G-1, C + 2, and C + 1) to increase conformational rigidity and chemical stability of the lower stem and *in vitro* transcription yield. The secondary structure of this RNA was determined using ^1^H, ^1^H NOESY spectra (Supplementary Table S4) in combination with information derived from an ^1^H,^15^N BEST-TROSY (Supplementary Table S4) spectrum (Fig. 2A and B) at 283 K. Typically, NOESY spectra show correlations of base paired imino protons in close proximity (≤6 Å) that allowed us to identify imino walks linking either sequentially neighbouring nucleobases or remote nucleobases in close distance through hydrogen bonding. Characteristic ^15^N chemical shifts of Gs and Us could be distinguished in ^1^H,^15^N-spectra, as chemical shifts of imino resonances (N1-H1) of Gs are expected between 140–150 ppm (^15^N) and 10–15 ppm (^1^H) while those of Us (N3-H3) are expected between 159–164 ppm (^15^N) and 10–15 ppm (^1^H).

The sequential connectivity of the locally C2-symmetric stretch of nucleotides (GGUGUGG) was ambiguous in the NOESY spectrum of s2m Delta_short (Fig. 2A). However, this ambiguity could be resolved by comparing the ^15^N BEST-TROSY spectra of the full-length construct (s2m Delta) and of the short construct. The imino signals of the full-length construct were unambiguously assigned in our previous work [17] and could be transferred to s2m Delta_short due to perfectly matching signal patterns (Fig. 2B). The ^1^H,^15^N imino resonance of G745 at 10.35 and 143.6 ppm implied a wobble base pair with U753, which does, however, not show an imino signal even at a low temperature of 283 K. The experimental data suggest the presence of a water-mediated G-U base pair for which only one, but not both imino protons are protected against solvent exchange as previously reported [93]. We also observed that G745 H1 was not present at higher temperatures, likely because of increased solvent exchange rates (Supplementary Fig. S2). We thus postulated this G745-U753 loop closing wobble base pair to be transient, which had also been shown in previous MD simulations of s2m Delta(76).

**Figure 3.**
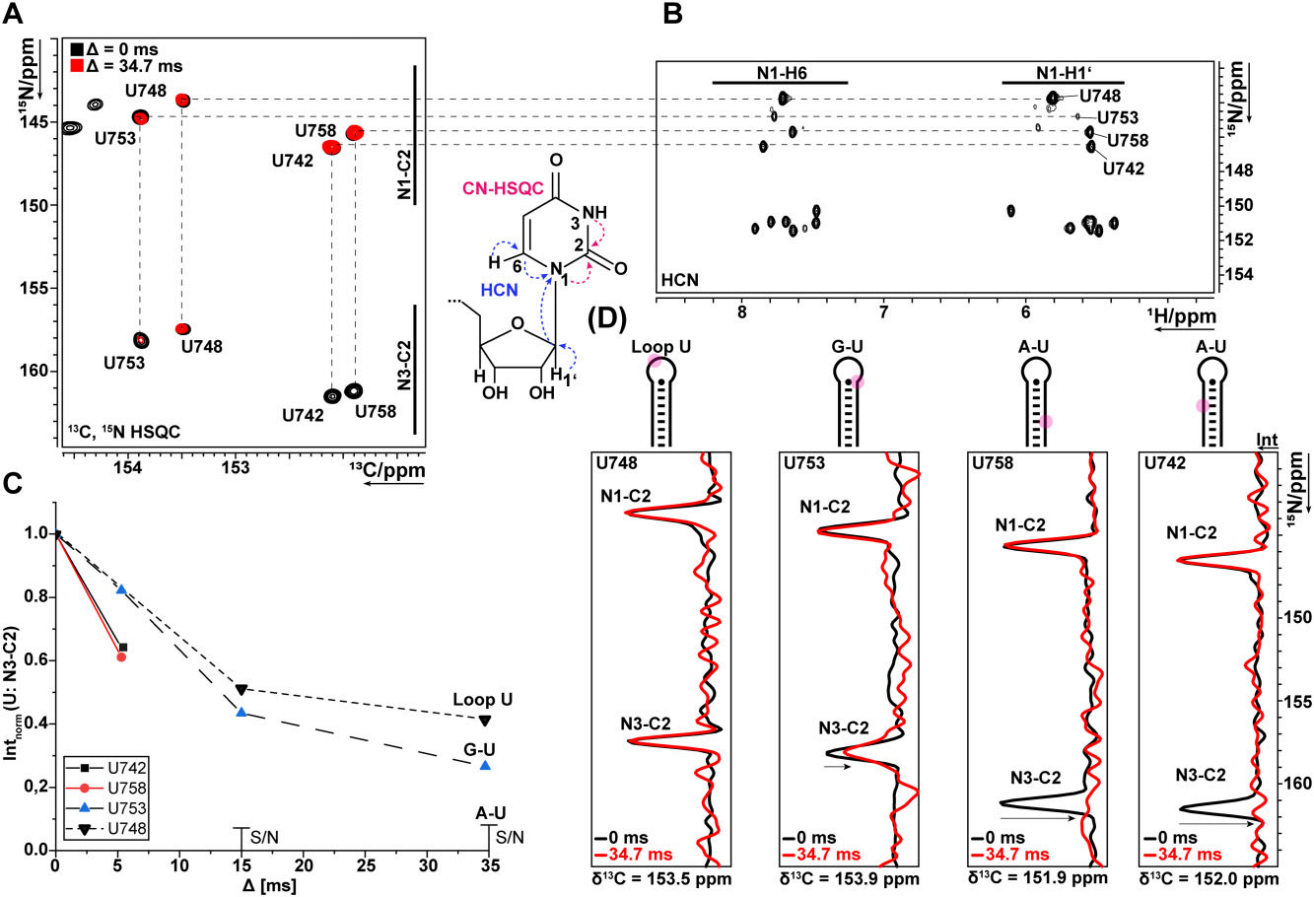
Analysis of N3-C2 modulation in s2m Delta_short using a ^13^C, ^15^N-HSQC experiment with deuterium filter. (**A**) ^13^C, ^15^N-HSQC measured at 308 K with sample#10. Exemplary magnetization transfer shown for CN-HSQC and HCN experiments. (**B**) ^1^H,^15^N plane of HCN experiment to assign N1 chemical shift of uridines measured with sample#3 at 308 K. Dashed lines indicate the N1 chemical shift transfer. (**C**) Normalized intensity of N3-C2 signals to N3-C2 signal intensity at deuterium filter delay Δ of 0 ms. (**D**) Projections along the ^15^N axis with (red) and without delay Δ (black). Projections were scaled to match N1-C2 signal intensity. Arrows indicate signal modulation in N3-C2 correlations for U748, U753, U758, and U742.

To further characterize the transient nature of the loop closing G-U base pair, we performed a deuterium-filtered low-γ ^13^C, ^15^N-HSQC experiment at 308 K [94] (Fig. 3 and Supplementary Table S6). This experiment avoids the excitation and detection of solvent-exchanging imino protons and reports on fast solvent exchange of hydrogen imino protons (N3-H3) using carbon-nitrogen correlations (N1-C2, N3-C2) as described in [94] and [95]. Here, we incorporated a ^15^N-^2^H spin echo delay after the initial INEPT transfer (Δ) optimized for 1/^1^*J*(N,D) with ^1^*J*(N,D) = (*γ*D/*γ*H)* ^1^*J*(N,H) to modulate the signal according to the solvent exchange properties of imino protons for the RNA that was dissolved in D_2_O. For s2m Delta__short at 308 K, the N3-C2 for A-U base pairs (U742, U758) showed line broadening with Δ = 34.7 ms, indicating slow solvent exchange, whereas unpaired loop nucleotides (U748) showed positive and unchanged (normalized to N1-C2) carbon-imino nitrogen correlation. For U753, we could delineate an intermediate solvent exchange even at 308 K as manifested by a reduced but still positive N3-C2 signal [94].

Thus, we confirmed the secondary structure of s2m Delta_short to be the same as the NMR-derived secondary structure of s2m Delta, except for two additional base pairs in the upper stem (Fig. 2D). The G742 to U742 mutation stabilized the upper stem of s2m Delta and s2m Delta_short, so that the transient G-U base pair between G745 and U753 could be observed in NOESY spectra recorded at 283 K and validated with ^13^C, ^15^N deuterium filtered experiments, which we similarly described previously for the full-length s2m Delta RNA construct [17] (Fig. 2B).

### NMR chemical shift assignment of s2m Delta_short

To determine a 3D NMR structure, a near-to-complete chemical shift assignment of NMR signals is required. Starting from the imino proton chemical shift assignments of s2m Delta_short, we assigned the aromatic signals C8H8 (G) and C6H6 (U) using correlation peaks observed in HCCNH experiments [96, 97] (Supplementary Table S6) that link the imino protons to aromatic protons (Supplementary Fig. S3). For nucleotides with an imino proton assignment, all aromatic resonances were thus assigned in ^13^C HSQC spectra (Supplementary Table S6) with the exception of G-2 and G745. Chemical shift assignments of the corresponding C1’H1’ resonances were derived from 3D pyrimidine- and purine-specific HCN experiments [98] (Supplementary Table S6) which correlate aromatic signals (C8H8 G, A; C6H6 C, U) with C1’H1’ signals via their connecting nitrogen N1 (U, C), N9 (A, G).

**Figure 4.**
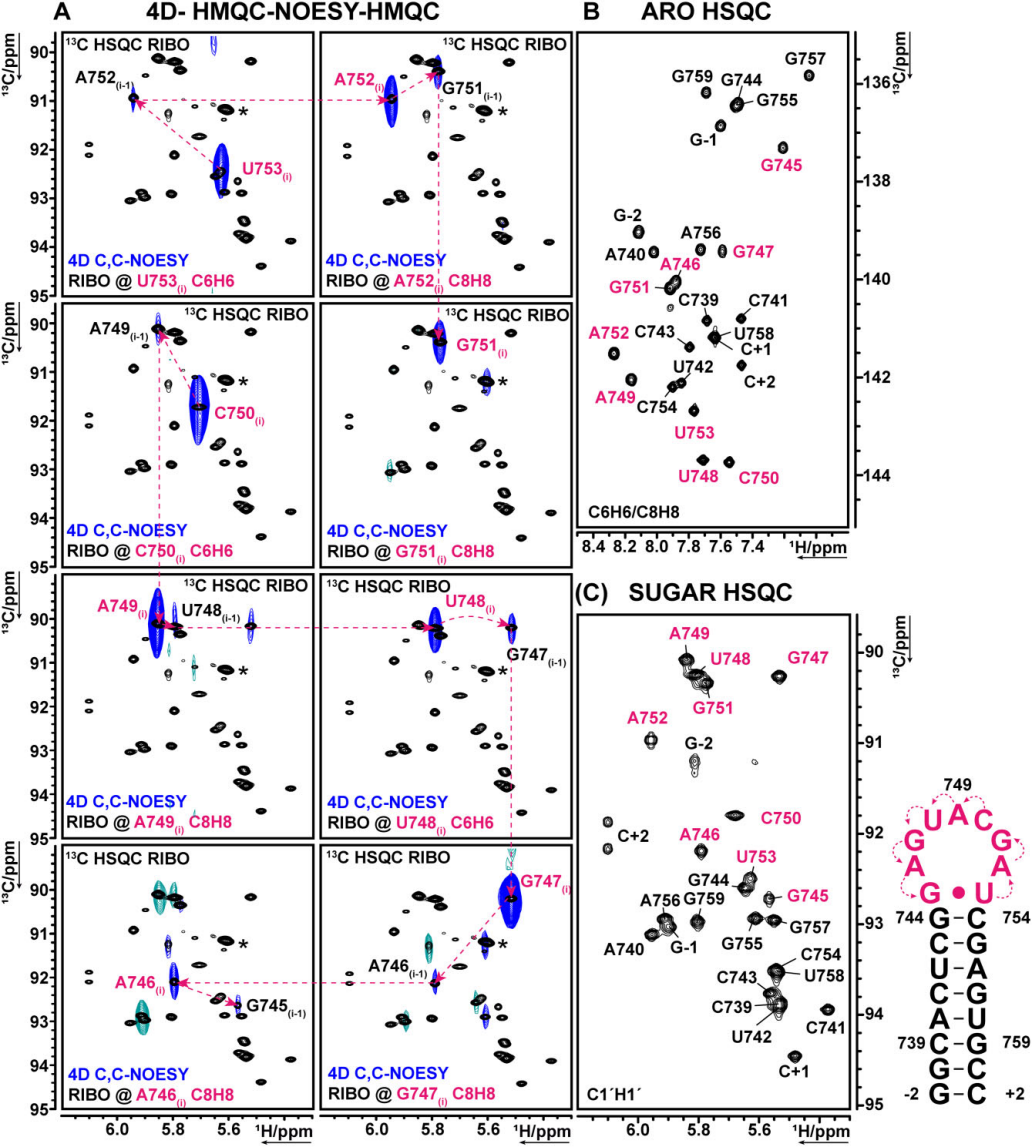
4D HMQC-NOESY-HMQC planes of nucleotides G745-U753 (pink) in s2m Delta_short. (**A**) In extracted C1’H1’-2D planes of the 4D, a sequential NOESY walk is depicted (pink arrows). Starting from an aromatic signal of a specific nucleotide (i) peaks are visible for the C1’H1’ resonance corresponding to the same nucleotide and to the previous nucleotide (i-1) (blue). By following these connectivities through the planes from 3′ to 5′ direction, a sequential assignment is achieved. Overlay of the 4D HMQC-NOESY-HMQC spectrum (blue, sample#4, Supplementary Table S1) and ^1^H,^13^C-HSQC spectrum (black, sample#4, Supplementary Table S1). Both spectra were recorded at 308 K. The resonance assignment of all nucleotides is annotated and the C6H6/C8H8 aromatic chemical shift leads to the shown planes. The respective nucleotide for that plane (pink) is annotated in the lower left corner of each spectrum. Degradation peaks are visible of s2m Delta_short from sample#4 (*). (**B**) Aromatic ^1^H,^13^C-HSQC at 308 K (sample#3, Supplementary Table S1). (**C**) C1’H1’ region of s2m Delta_short at 308 K (sample#3, Supplementary Table S1).

Severe signal overlap and missing crosspeaks prevented the H6/8-H1’ sequential walk in 2D NOESY spectra (Supplementary Fig. S4). Even 3D-NOESY (Supplementary Table S6) spectra allowed only for ambiguous assignment due to signal overlap (Supplementary Fig. S5). The classic route of NMR chemical shift assignment relies on sequential aromatic-anomeric proton NOEs observed in A-helical motifs. For s2m Delta_short, the nonhelical loop regions accounted for about a third of the complete RNA construct, thus leaving the whole stretch of loop nucleotides (A746–U753) ambiguous. We thus recorded a 4D HMQC-NOESY-HMQC [99] experiment (Supplementary Table S6) that resolved all C8H8 and C6H6 signals. Analysis of the 4D experiment allowed a close-to-complete chemical shift assignment of aromatic and C1’H1’ resonances for all nucleotides (Fig. 4, and Supplementary Figs S6 and 7). In representative C8H8, C6H6 2D planes, NOE cross peaks to their intraresidual C1’H1’ (i) and sequential C1’H1’ (i-1) of the 5′-terminal nucleotide could be resolved (Fig. 4). Overlay of these planes with ^1^H,^13^C HSQC spectra in the C1’H1’ region showed the correlating signals. Even without prior knowledge of the nucleotide identity belonging to the aromatic resonance, a sequential assignment was possible by correlating each resonance with the one of the subsequent nucleotide. Selectively labelled (^13^C^15^N C, ^13^C^15^N AU) samples were used to further validate our assignment and to achieve an almost complete assignment of the ribose H1’-H5’’ signals using *fw*-HCCH-TOCSY NMR spectra [50] (Supplementary Table S6), except for C3’H3’ of G-2, at 308 K (Supplementary Fig. S8). The chemical shift assignment of s2m Delta_short resonances (^1^H: 92%, ^13^C: 90%, ^15^N: 82%) is listed in Supplementary Tables S12–14 and was deposited in the BMRB with the ID: 34919.

### Heteronuclear NOEs depict dynamic behaviour of the loop nucleotides

**Figure 5.**
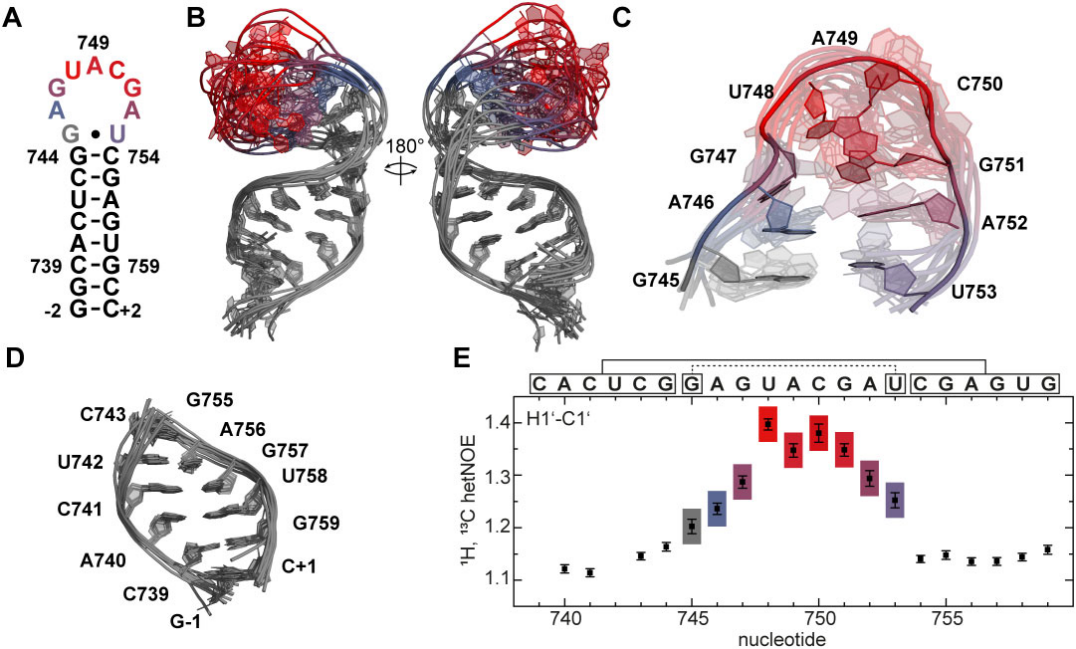
NMR solution structure of s2m Delta_short RNA. (**A**) Secondary structure of s2m Delta_short. The loop nucleotides are highlighted in a hetNOE-derived heatmap (red: high, blue: low) from panel (E). (**B**) Aria-calculated NMR solution structure ensemble of 10 superimposed structures with the lowest χ^2^ to SAXS data. Loop sequence highlighted in hetNOE derived colours (red: high, blue: low) from panel (E). The overall RMSD was calculated to be 3.60 ± 1.13 Å. (**C**) Aligned and overlayed loop sequence with an RMSD of 3.31 ± 0.77 Å. (**D**) Overlaid stem region without G-2-C + 2 base pair and G744-C754 base pair. The stem ensemble of all 20 structures converges with an RMSD of 0.94 ± 0.23 Å (only 10 structures shown). (**E**) ^1^H,^13^C heteronuclear NOE of s2m Delta_short for sugar C1’H1’ resonances (sample#4.5). The RNA sequence is given on the top of each plot with squares indicating base paired regions.

To investigate the local subnanosecond dynamics faster than the overall rotational correlation time *τ*_c_ of s2m Delta_short, we recorded ^1^H,^13^C heteronuclear NOE (hetNOE) experiments for the aromatic C6H6/C8H8 (Supplementary Fig. S9) and ribose C1’H1’ (Fig. 5E) signals. As NMR parameters relevant for relaxation, especially chemical shift anisotropy, of ribose atoms are more similar to each other than the aromatic CH groups, respectively, C1’H1’ hetNOE values for different nucleotides can readily be compared. We focus therefore on the interpretation of the data for these resonances to derive qualitatively differences in local dynamics. We determined mean hetNOE values of 1.14 for stem nucleotides and 1.30 for the loop nucleotides. Within the loop, the hetNOE increased from 1.20 at the start of the loop (G745) up to 1.40 at the tip of the loop (U748) before gradually decreasing again to 1.25 (U753). The hetNOE relaxation data thus support a highly dynamic character of the nonaloop.

### NOE-based NMR solution structure of s2m delta_short

To determine the NMR solution structure of s2m Delta_short, ^1^H, ^1^H NOE distance restraints were obtained using NOESY spectra (Supplementary Table S6) of an unlabelled sample. 382 NOE cross peaks were incorporated into the structure calculation and are schematically illustrated in Supplementary Fig. S10. In addition, NOEs from 4D HMQC-NOESY-HMQC experiment (C1’-H1’-C6/8′-H6/8′) were included in the structure calculations. The average pairwise root-mean-square deviation (RMSD) of the stem region (without the loop closing base pair, G744-C754 and G-2-C + 2) was 0.94 ± 0.23 Å (Fig. 5D). The average pairwise RMSD of only the loop nucleotides (G745-U753) was 3.60 ± 1.13 Å (Fig. 5C). The overall structure resembled a stem-rod like RNA with a kink at the transition between the stem and the loop segment with an average pairwise RMSD of 3.31 ± 0.77 Å (Fig. 5B).

The average number of interresidual NOEs per nucleotide was six and the average number of intraresidual NOEs per nucleotide was ten (Supplementary Table S15). For the loop nucleotides, a lower number of interresidual NOEs was found due to the unstructured and dynamic nature of the loop residues or the resultant larger averaged distances and NOE scaling due to local dynamics between residues, consistent with increased values of the hetNOE data. Except for the G745-U753 hydrogen bond and planarity base pair restraints, only NOE restraints were implemented for the loop nucleotides in the structure calculations. This sparsity of restraints precluded convergence of the loop region in structure calculations. We note that restraining distances and torsions to derive from a single conformation already constricts the conformational space of the RNA and that the NOE intensities might derive from many different conformations that, if implemented, could enforce artificial convergence in the ARIA structure calculations. Notably, for U753, ^13^C, ^15^N-HSQC measurements indicate imino base pairing (Fig. 3) which validates the use of this base pair restraint as previous studies have shown before [95].

### NMR ensemble comparison with SAXS data

Small-angle X ray scattering data of the Delta_short RNA were measured at two concentrations (1.5 and 2.5 mg/ml) (Supplementary Figs S11 and 12). No dimerization was observed at higher concentrations as the calculated molecular weight (7.3 kDa) roughly corresponded to a monomeric distribution. Inter-particle repulsion was observed at 2.5 and at 1.5 mg/ml in scattering curves which is why further processed SAXS scattering curves (as described in “Materials and methods” section, Supplementary Fig. S13) were used to compare with back calculated scattering curves from ARIA NMR ensemble members (Supplementary Table S11). This procedure allowed us to identify 10 conformational states in best agreement with experimental SAXS data available (models: 1, 3–6, 8, 10, 15, 17, 19; Supplementary Figs S14 and 15). The CRYSOL fits for the two concentrations measured yielded almost the same set of 10 best fitting structures (Supplementary Table S11). An improvement was observed in χ^2^ values when comparing modified structure ensembles (5TP3cP) and processed SAXS data at 1.5 mg/ml. The overall averaged back calculated χ^2^ value for processed SAXS data at 1.5 mg/ml was 6.077 (10 best χ^2^ = 3.889, 1 best χ^2^ = 2.701). These χ^2^ values are in good agreement with the NMR structure as seen in other publications [95, 100, 101]. The high χ^2^ values for SAXS data at 2.5 mg/ml can be explained by an increase in inter-particle repulsion at this concentration. When merging the SAXS data as described in “Materials and methods” section [64], the χ^2^ values decrease significantly. The *R*_g_ and *D*_max_ values of all scattering curves are in the range of *R*_g_ = 1.39–1.5 nm and *D*_max_= 4.0–5.0 nm (Supplementary Figs S11–13). However, we note that the limitations of available software did not allow us to calculate the back calculated scattering curve of the NMR structure ensemble as a whole, instead scattering curves were calculated for each individual ensemble member.

The *R*_g_ and *D*_max_ values determined with HullRad from the NMR structure ensemble are in a range of *R*_g_ = 1.28–1.44 nm and *D*_max_= 3.99–4.85 nm (Supplementary Table S16). An *R*_g_ and *D*_max_ discrepancy of maximally 15% was observed with all ARIA structure ensemble members. For the best fitting model, the discrepancy was only 4%. *Ab initio* model building was performed and compared to ARIA structure ensemble members (Supplementary Fig. S16). We were able to identify good agreement of the global geometry using DAMMIN and DENSS models (Supplementary Fig. S16) but also identified artefacts in the low-resolution models. These artefacts may be explained by slower time scale motions detected exclusively during SAXS measurements, such as helix fraying at the stem closing base pairs (see also provided movies, mov1 and mov2). We further note that to properly address the *ab initio* modelling of the s2m Delta_short RNA it would require a differentiation of the electron density into one belonging to the rather rigid stem and one with lower resolution corresponding to the loop. Considering the limitations of used software we cannot accurately describe the examined system here. In conclusion, we used SAXS data to refine and analyse the ARIA NMR structural ensemble. We note that discrepancies are observed in χ^2^, *R*_g_, and *D*_max_ values. However, these discrepancies could arise from the different temperatures (SAXS was measured at 298 K and NMR data for structure calculations were collected at 308 K) and from the different buffer conditions. The discrepancies in *D*_max_ and *R*_g_ (maximal 15% deviation) between SAXS and NMR data hint at dynamics still unaccounted for, motivating further investigation into the dynamic behaviour of the nonaloop nucleotides with a combined NMR and MD approach.

### Sugar pucker averaging of s2m loop nucleotides

**Figure 6.**
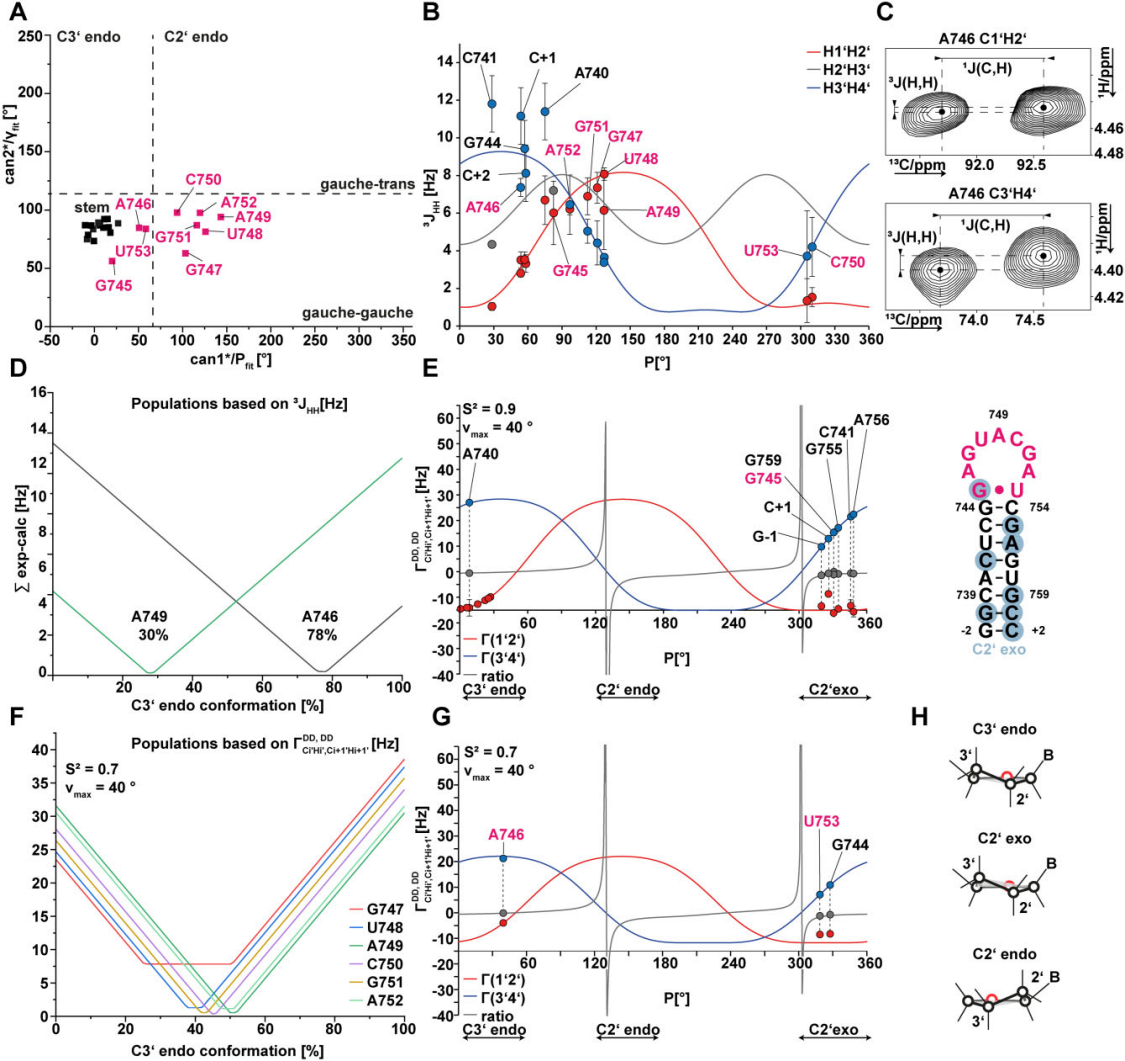
Sugar pucker conformation of s2m Delta_short determined by canonical coordinates, ^3^*J* (H, H) coupling constants and CCR at 308 K. (**A**) Canonical coordinates determined from ^13^C chemical shift assignments. (**B**) Determination of pseudorotation phase *P* from ^3^*J* (H1’, H2’), (H3’, H4’), and (H2’, H3’) coupling constants determined via a fw-HCC-TOCSY-CHH-E.COSY experiment. (**C**) fw-HCC-TOCSY-CHH-E.COSY experiment from A746 signals to determine ^3^*J* (H1’, H2’, and H3’, H4’) coupling constants. (**D**) C3’-endo population distribution of A746 and A749 derived from ^3^*J* (H, H) coupling constants. (**E**) Determination of pseudorotation phase *P* based on mean $\Gamma _{C1\prime H1\prime ,C2\prime H2\prime }^{DD,DD}$ and $\Gamma _{C3\prime H3\prime ,C4\prime H4\prime }^{DD,DD}$ from 2D and 3D quantitative Γ-HCCH experiment (*ν*_max_ = 40°, *S*^2^ = 0.9). Shown are the fitted values from more rigid nucleotides are shown. (**F**) C3’-endo population distribution of loop nucleotides based on $\Gamma _{C1\prime H1\prime ,C2\prime H2\prime }^{DD,DD}$ and $\Gamma _{C3\prime H3\prime ,C4\prime H4\prime }^{DD,DD}$. An *S*^2^ parameter of 0.7 was assumed for dynamic nucleotides. (**G**) Determination of the pseudorotation phase *P* from mean $\Gamma _{C1\prime H1\prime ,C2\prime H2\prime }^{DD,DD}$ and $\Gamma _{C3\prime H3\prime ,C4\prime H4\prime }^{DD,DD}$ from 2D and 3D quantitative Γ-HCCH experiments (*ν*_max_ = 40°, *S*^2^ = 0.7). Shown are the fitted values of more dynamic nucleotides. (**H**) Schematic representation of sugar pucker conformations (plane coloured in light grey).

The pseudorotation phase *P* and amplitude *v*_max_ report on the ribofuranosyl ring conformation. To describe the ribose puckering of s2m Delta_short, the pseudorotation phase *P* was derived using three different NMR parameters: analysis of canonical coordinates ([48 and 49]), ^3^*J* (H_i_, H_i+1_) coupling constants [50, 51], and CCR rates $\Gamma _{{\mathrm{Ci}}\prime {\mathrm{{\rm H}i}}\prime ,{\mathrm{Ci}} + 1\prime {\mathrm{Hi}} + 1\prime }^{{\mathrm{DD}},{\mathrm{DD}}}$ [54]. All three NMR methods are sensitive to torsion angle averaging up to the millisecond time scale. In general, ribose moieties in RNA can adopt either a north conformation (*P* = 270°–90°) or a south conformation (*P* = 90°–270°). While *v*_max_ is supposed not to vary substantially, typical values of *P* for A-canonical RNA nucleotides are between 0° and 36°. Values between 144°–180° represent the C2’-endo conformation. The ^13^C canonical coordinates of s2m Delta_short showed C2’-endo conformation for all loop nucleotides except for G745, A746, and U753 (Fig. 6C). These are the nucleotides closest to the stem, and they adopt a C3’-endo conformation as do the stem nucleotides.

*P* values derived from ^3^*J* (H, H) coupling constants are summarized in Supplementary Table S17. For stem nucleotides (C737, C738, A740, and C741), *P-*values between 25° and 70° were derived. Interestingly, the reported ^3^*J* (H3’, H4’) values of stem nucleotides were higher than expected from computed parametrization curves, a behaviour previously observed [52]. *P*(A746) calculated from ^3^*J* (H, H) coupling constants was 53.6° which corresponds to a C3’-endo conformation, in line with the canonical coordinates.

Assuming conformational averaging between a C3’-endo conformation with *P*= 18 and a C2’-endo conformation with *P*= 162° the conformational heterogeneity was modelled by a two-state-model as described in “Materials and methods” section. For A746 using the ^3^*J* (H, H) coupling constants a 78% distribution of C3’-endo conformation was derived. This distribution towards more C3’-endo conformation agreed with the canonical coordinates as well. *P*(G747, U748) were ∼120°. Assuming the two-state-model, their fraction of C3’-endo population was between 0% and 40%. The *P-*value for A749, the nucleotide most distant from the stem, was 127° when fitted to a single conformation. The two-state-model yielded a population of 30% of C3’-endo conformation. *P-*values of 300° obtained for C750 and U753 corresponded to a C2’-exo conformation. The two-state-model predicts 40%–100% in C3’-endo conformation for both nucleotides. According to ^3^*J* (H, H) coupling constants, all loop nucleotides experience ribose conformational averaging. Only for A746 (closest to the stem) and A749 (at the top of the loop), we were able to obtain a clear two-state distribution of 78% in C3’-endo for A746 and 30% in C3’-endo for A749, while for the other residues, more substantial multistate populations seem to be adopted (Supplementary Fig. S17 D ).

*P-*values were additionally derived from quantitative 2D Γ-HCCH and 3D *fw*-Γ-HCCH experiments [54, 102] and are summarized in Supplementary Table S18. Data derived from both independent measurements yielded highly similar results (Supplementary Fig. S17A and B) and were used to derive a mean value. The mean CCR rates $\Gamma _{{\mathrm{C}}1\prime {\mathrm{{\rm H}}}1\prime ,{\mathrm{C}}2\prime {\mathrm{H}}2\prime }^{{\mathrm{DD}},{\mathrm{DD}}}$ and $\Gamma _{{\mathrm{C}}3\prime {\mathrm{{\rm H}}}3\prime ,{\mathrm{C}}4\prime {\mathrm{H}}4\prime }^{{\mathrm{DD}},{\mathrm{DD}}}$ of s2m Delta_short nucleotides were fitted to parametrization curves (Fig. 6E). The parametrization curves were calculated with a *τ*_c_ of 3.9 ns. The global correlation time was derived from *T*_1_, *T*_1ρ_ (Supplementary Fig. S18) and hetNOE values at 600 and 800 MHz as described in “Materials and methods” section and summarized in Supplementary Table S19.

As depicted in Fig. 6E, some CCR rate values belonging to more rigid nucleotides (stem) were fitted to *P-*values between 320° and 350° with a *ν*_max_ of 40° and *S*^2^ of 0.9. These correspond to C2’-exo conformation and are categorised in the north conformation. The C2’-exo conformation is populated to a significant extent in the RNA structure database [103] and is energetically speaking not separated by a barrier from C3’-endo conformation. The nucleotides G744, A746, and U753 that could not be fitted in *E* with a *ν*_max_ of 40° were fitted with variable *ν*_max_ (Supplementary Fig. S17C) and *S*^2^ of 0.7 (Fig. 6G).

CCR rate values of A746 yielded a *P*-value of 39.6°, corresponding to a C3’-endo conformation, again in agreement with both canonical coordinates and ^3^*J* (H, H) coupling constants. For G744 and U753, we obtained *P-*values in the C2’-exo conformation region, similar to the stem nucleotides. CCR rate values of loop nucleotides (G747-A752) could neither be fitted by reducing *S*^2^ nor by changing *ν*_max_. Instead, we performed a two-state fitting. The population distribution of most loop nucleotides showed a clear 40%–50% population fraction in the C3’-endo conformation, confirming the averaging of ribose conformation in loop nucleotides G747-A752 (Fig. 6F).

The χ angle defines the relative orientation of the ribose ring and the nucleobase. We determined the χ angle for stem nucleotides A740, C741, G755, and loop nucleotides A749 and G751 using CRR rates $\Gamma _{{\mathrm{C}}1\prime {\mathrm{{\rm H}}}1\prime ,{\mathrm{C}}6{\mathrm{H}}6}^{{\mathrm{DD}},{\mathrm{DD}}}$ and $\Gamma _{{\mathrm{C}}1\prime {\mathrm{{\rm H}}}1\prime ,{\mathrm{C}}8{\mathrm{H}}8}^{{\mathrm{DD}},{\mathrm{DD}}}$ (Supplementary Table S20, and Supplementary Fig. S17E and F). Low signal intensities in the cross experiment prevented quantitative analysis for the remaining nucleotides. Nucleotides in A-helical RNA adopt anti-conformation. The here determined cross-correlated relaxation rates (CCRs) all corresponded to an *anti-*conformation for stem nucleotides A740, A741, and G755 and for loop nucleotides A749 and G751.

### Conformational space of s2m nonaloop by molecular dynamics

MD simulations were performed to explore the structural space available to s2m Delta_short based on the 20 states of the ARIA-derived conformational ensemble as starting structures. To establish a baseline of the structural and dynamical data, the major dynamics and corresponding free energy profile (Supplementary Fig. S19A) from the 20 μs of aggregated MD data was represented by the first two PCs (PC1 and PC2). The GMM density distribution revealed two basins, corresponding to CSs: one with broad conformational sampling across the entire PC1–PC2 space, separated from only one other resolvable cluster of structures. However, the limited variance captured in the first two dimensions (32%) indicated a relatively weak structural correlation, which would not adequately depict the full range of dynamics sampled over 20 μs. Therefore, to resolve CSs beyond the PC1–PC2 dynamic, it was necessary to include the first 20 PCs to capture ∼90% variance (Supplementary Fig. S19B), where the structural ensemble was decomposed into ten CSs and projected on to PC1 and PC2 with points coloured according to their assigned substate. The overlapping of clusters in PC space suggested that many more than the first two modes would be required to obtain a full description of the simulated dynamics, consistent with reasonable expectations of highly dimensional nonlinear dynamics from different initial conditions. The structural change for each CS (Supplementary Fig. S19C) is shown by the RMSD over time for each simulation, with colours corresponding to CS assignments. Several CSs, such as CS1, were visited by multiple independent simulations, while others, such as CS6, were visited by only one simulation. Structural analysis (Supplementary Fig. S19D and E) revealed that CS6 was characterized by a large magnitude “flipped-back” conformation of the terminal loop, rationalizing why only this CS stood out as a distinct cluster of points in the PC1–PC2 space. It is interesting to note that this state has qualitative similarities with the terminal loop region of the SCoV-1 s2m crystal structure (PDB: 1XJR), which is stabilised into a “kinked” conformation by a GC-quartet and nucleobase stacking [16, 20]. Given previous reports of differences in s2m kissing homodimerization through the terminal loop in SCoV-1, SCoV-2, and Delta, differences in loop dynamics, such as accessibility of a “kinked” or “flipped-back” conformation, may be important in explaining differences in dimerization [18–20, 76].

Due to the aggregated nature of the MD data, however, it is unclear whether CS6 and others were over- or underrepresented by the proportions they appeared in the MD data; indeed, the fact that some CSs were not visited by distinct independent simulations provided a relatively weak basis for estimating the true timescale of these dynamics. To better understand how these states are likely to contribute within true structural ensembles studied by biophysical experimentation, validation, and reweighting of the structures sampled by MD according to means of experimental observables was necessary.

### Integrative ensemble reweighting

The challenge of interpreting NMR data, especially for the extensive conformational dynamics of heterogeneous RNA structural ensembles, has been previously reported [38]. Considerable effort has been devoted to integrating experimental and theoretical data for precise characterization of biomolecular conformational heterogeneity, with one prominent approach utilizing ensemble reweighting that adjust unbiased populations based on experimental data to better align with back-calculated observables [52, 90, 104–109]. To this end, we performed MD simulations of s2m Delta_short and refined and validated the resulting structural ensemble against experimental NMR data. Specifically, BME reweighting was applied to refine the MD ensemble and improve agreement with experimental observables using three different sets of NMR data: NOE distances, ^3^*J* scalar coupling constants, and CCR rates. This process generated four distinct reweighted ensembles, each of the first three explicitly optimizing agreement to one respective NMR observable, and the last using all three types of NMR data together. The root mean squared errors were compared alongside the unweighted MD ensemble. The outcome of this integrative approach were ensembles of highly dynamic structures that better reconciled with the experimentally measured observables, particularly for the flexible apical loop region. For each of these four ensembles, we back-calculated values for NOEs, ^3^*J* coupling constants, and CCR rates, and evaluated their agreement with experimental data (Supplementary Fig. S20).

As expected, relative to the reweighted ensembles, the unweighted MD ensemble exhibited high root-mean-square-error (RMSE) across all three observables (RMSE_NOE_ = 0.8 Å; RMSE_3J_ = 2.6 Hz; RMSE_CCR_ = 8.7 Hz) and generally had the most violations with experimental mean values (Supplementary Fig. S20D). While agreement with NOE distances was already strong in the MD ensemble, reweighting based on NOEs significantly improved agreement with NOE distances, as reflected by a 0.6 Å reduction in RMSE and a reduction of NOE violations from 60 to 32 following BME optimization (Supplementary Fig. S20A). Additionally, the NOE-based reweighting modestly improved agreement with ^3^*J* scalar coupling constants by ∼0.1 Hz and CCR rates by ∼0.6 Hz compared to the unweighted MD data, though this still corresponded to most couplings and CCR rates being in violation (Supplementary Fig. S20D).

The ^3^*J*-based reweighting improved agreement for ^3^*J* coupling constants and CCRs, reducing RMSE by ∼1 and 3 Hz, respectively, but did not perform as well for NOEs, increasing RMSE by 0.1 Å and introducing nine new NOE violations (Supplementary Fig. S20D). This is not unexpected, given the more localised nature of scalar coupling constraints on the ensemble relative to through-space NOE distances. Essentially, the ribose conformation outside the structural context of A-form helices is not very sensitive to the structure of its neighbouring nucleotides. Thus, by optimizing the MD ensemble for ribose conformation agreement with experimental data, some medium-range structural information was likely lost. Additionally, the computed ^3^*J* values suffer from requiring empirically derived Karplus parameterization [110]. Applying parameterizations developed using different systems can introduce inaccuracies, resulting in the estimated ³*J* coupling constants possibly not reflecting the true coupling constants accurately [53].

Strikingly, the CCR-based reweighting yielded the best overall improvement among the single-observable ensembles, reducing RMSE for CCR rates by *∼*4 Hz and the number of violations from 23 to 8 while also improving ^3^*J* scalar coupling constants accuracy by RMSE of *∼*0.6 Hz without greatly degrading NOE agreement relative to the unweighted MD ensemble (negligible difference in RMSE and only two new violations). This result suggested that CCR-based refinement not only captured key dynamic features but also indirectly enhanced structural accuracy in a way that aligned with scalar coupling data. Notably, the model for CCR is parameter-free, depending only on the maximum amplitude *v*_max_ and the order parameter *S*^2^.

Reweighting carried out according to all NMR observables yielded an ensemble with intermediate performance across the board. As shown in Supplementary Fig. S20D, the reweighting based on all NMR observables slightly increased NOE error relative to MD and reduced RMSEs relative to the unweighted MD baseline for ^3^*J* coupling constants and CCR rates but did not outperform the best single-observable ensemble for any one measurement. Specifically, compared to the CCR-only reweighted ensemble, the “All” ensemble increased RMSE for CCRs (from 4.3 to 5.3 Hz) and for ^3^*J* coupling constants (from 1.6 to 1.8 Hz) while offering no improvement in NOE agreement. Thus, using all NMR observables in this case produced a more uniform but less optimal agreement across datasets. This reinforces the value of careful selection of observables in ensemble refinement, especially given challenges emerging from satisfying multiple length- and timescales.

Ultimately, these findings highlight the importance of incorporating diverse NMR observables in ensemble refinement and suggest that CCR-based reweighting may provide an effective strategy for improving MD-derived RNA ensembles of dynamic regions such as nonaloops. However, we caution that reweighting to a single observable introduces limitations, potentially biasing the ensemble towards conformations that are overly optimized for a particular dynamical timescale or interaction type. The observed deviations in NOE accuracy between ensembles underscores this point. Nonetheless, for the current study, we conclude that CCR-based reweighting provides the most consistent and interpretable improvement to the MD-derived ensemble, albeit on the basis of fast, local dynamics influencing CCRs.

### Conformational space of s2m nonaloop by CCR-based reweighted ensemble

**Figure 7.**
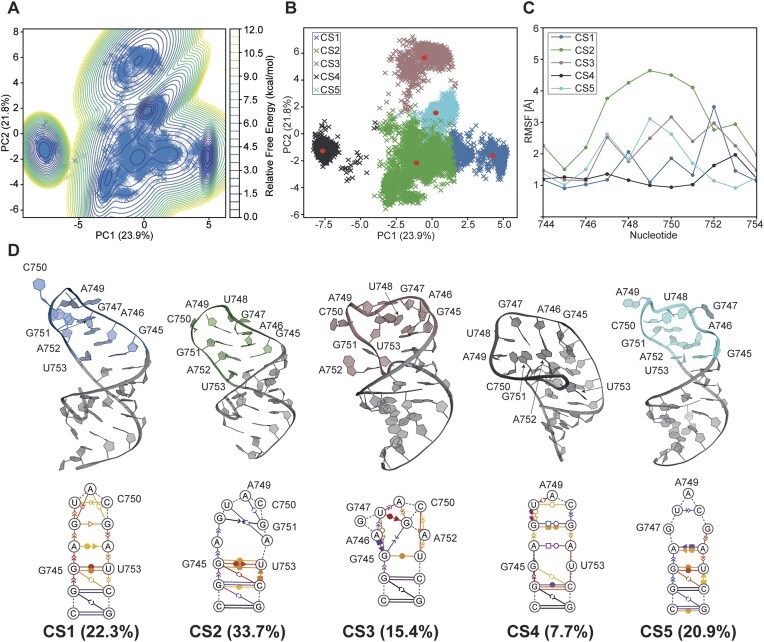
Structure and implied dynamics of the CCR reweighted ensemble. (**A**) PC plot with estimated free energies, revealing approximately six densely sampled regions, informing (**B**) *k*-means clustering into five CSs with respective centroids (red dots) falling as close as possible to densely sampled structures. (**C**) RMSF of the terminal loop nucleotides for each CS. (**D**) Threedimensional structures of CS centroids, proportion of data falling within each CS, and dynamic secondary structures representing the proportion of structures in each CS.

To better understand the structural heterogeneity of the CCR-reweighted ensemble, we performed PCA on the sampled conformations and identified distinct CSs using *k*-means clustering. Immediately, we found that a greater proportion of variance was captured within the first two PCs relative to the aggregated MD simulation data (Supplementary Fig. S21), suggesting that loop dynamics are relatively simple enough to be characterized by combinations of a smaller number of dynamic modes. The free energy landscape projected onto the first two PCs (Fig. 7A) revealed multiple densely populated regions, indicating the presence of several stable conformational states. Clustering analysis (Fig. 7B) further resolved these regions into five major CSs (CS1–CS5), which collectively describe the dominant structures and dynamics of the ensemble.

**Figure 8.**
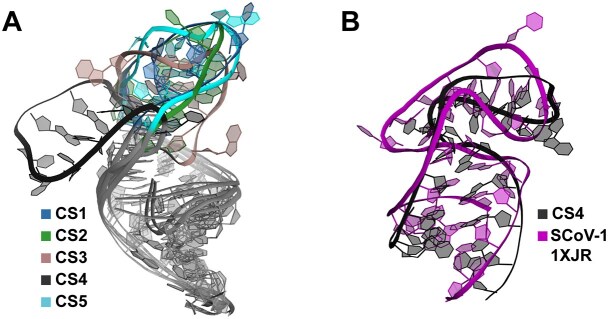
Flipped-back conformation in s2m Delta_short. (**A**) Overlayed centroid structures of the CCR reweighted ensemble (CS1 to CS5). The dominant dynamic in the data is a flipped back terminal loop (CS4) sampled by only one out of the original 20 MD simulations. (**B**) Overlayed centroid structure CS4 with apical GNRA-like pentaloop of SCoV-1 (PDB: 1XJR).

The structural features defining each CS highlighted the flexibility of the s2m Delta_short apical loop. Representative centroid structures for each substate (Fig. 7D) illustrated a range of distinct loop conformations, with varying degrees of nucleobase stacking, flipping, and dynamic interactions between nucleotides. CS1 was a relatively populous (22.3%) substate, characterized by an open loop conformation with relatively fleeting stacking and noncanonical base pairing, except stacking between successive nucleobases, which were observed in a greater proportion of structures. Notably, CS2 emerged as the most populated (33.7%) and dynamic substate according to root-mean-square-fluctuation (RMSF), exhibiting various fleeting noncanonical interactions between nucleotides. This was also rationalized by the presence of multiple stable basins in the free energy plot, suggesting multiple stable structures were encompassed by the single CS. CS3, though less populated (15.4%), represented an alternative state exhibiting a “pinched,” compact loop conformation with persistent nucleobase stacking between G745 and G751. The resultant backbone conformation led to heightened RMSF throughout the loop due to few noncanonical base pairs and fleeting stacking interactions to stabilize the nucleotides. Meanwhile, CS4 (7.7%) captured the “flipped-back” configuration of the apical loop (Fig. 8), a feature that was observed in <5% of the original unweighted MD ensemble but gained prominence following reweighting. Notably, RMSF for CS4 was measured to be consistently low (∼1–2 Å) in all nucleotides relative to its centroid structure, suggestive of significantly stabilizing noncanonical interactions. This was verified by the corresponding dynamic secondary structure plot, revealing high occupancy (dark purple) *trans* Watson–Crick/Hoogsteen base pairs (A746-A752 and G747-G751) and relatively fleeting Watson–Crick/Watson–Crick or base stacking interactions elsewhere. U753, for which the peak RMSF was measured in CS4, was also the only nucleotide that did not make any interactions prominent enough to meet the threshold for plotting. Finally, CS5 sampled a conformation wherein the lower loop nucleotides were constrained by persistent A752-A746 *cis* Hoogsteen/sugar edge base pair, reflected by low RMSF except for peaks at G747 and A749. RMSF in the loop is also slightly hampered by a U748/C750 nucleobase stacking interaction.

Importantly, the reweighting process not only enhanced agreement with experimental data but also revealed new insights into the structural landscape of the s2m Delta_short apical loop. Particularly, the identification of the flipped-back conformation in CS4 as a major contributor to ensemble structure and dynamics suggested the existence of previously underappreciated alternative states that could play a role in RNA function, as they are also present in SCoV-1 (Fig. 8B). This highlights the power of integrating MD simulations with experimental constraints, enabling a more nuanced characterization of RNA dynamics beyond the resolution of conventional structure determination methods.

It is interesting to note that the reweighted ensembles derived from ^3^*J* coupling constants, NOE distances, and all NMR observables simultaneously yielded differing descriptions of the free energy landscape (Supplementary Fig. S22). The ^3^*J* ensemble suggested that the nonaloop dynamics were given by a small collection of densely packed CSs with infrequent, high-energy transitions described by a small number of PCs (Supplementary Fig. S22A and B); this was likely an artefact from BME optimization reweighting a small collection of structures as very probable relative to the remainder of those simulated. This might indicate that the Karplus parameterization used for predicting ^3^*J* coupling constants from MD needed to be further refined for s2m. On the other hand, the NOE ensemble appeared to make only modest changes relative to the uniformly weighted MD ensemble. The screen plot suggested that the underlying loop dynamics were too complex to be described adequately in a low-dimensional linear subspace, with similar proportions of variance captured per PC (Supplementary Fig. S22C). The distribution of points in the PC1–PC2 projection was also similar to the unweighted MD, revealing a large, diffuse basin within the PC1–PC2 space separated from a narrower basin (Supplementary Fig. S22D). Finally, the ensemble combining CCRs, NOEs, and ^3^*J* coupling constants showed qualitative similarities with the ^3^*J* ensemble, though the greater temperature hyperparameter ensured enough diversity to produce three clearly defined basins (Supplementary Fig. S22E and F). Importantly, each ensemble contained a CS which we found still corresponded to the “flipped-back” loop conformation (data not shown), providing roughly equivalent descriptions of the largest magnitude dynamics within the data while adjusting the description of the associated free energy profiles based on the varying timescales of each data set. Ultimately, despite differences in the description of the free energy landscape, we found that BME supports ensembles providing a substantial expansion of dynamics beyond the ARIA starting structures while remaining consistent with diverse experimental observables.

### Pseudorotational analysis of CCR-based ensemble

**Figure 9.**
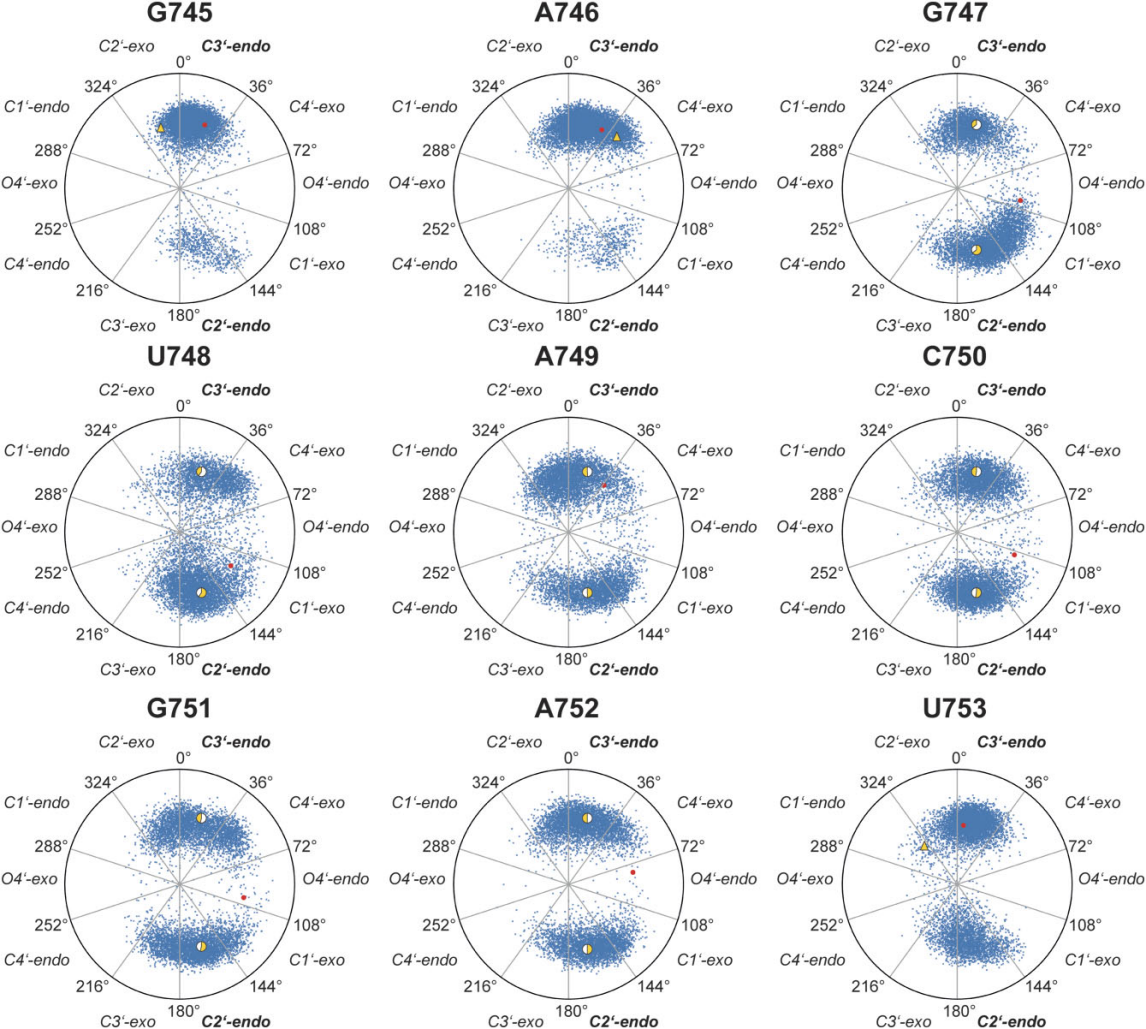
Pseudorotation angles and amplitudes of the CCR reweighted ensemble of loop nucleotide G745-U753. The computed mean is given by a red dot while the experimental derived pseudorotation phase from CCRs is given by a yellow triangle for one-state fits. The filled yellow circle is the derived percentage of population distribution from a two-state model with *P*= 18° for C3’-endo conformation and *P*= 162° for C2’-endo conformation.

The pseudorotation phases of the nucleotide ribose moieties in the apical loop exhibited distinct conformational preferences in the CCR-reweighted ensemble, as illustrated in Fig. 9. Compared to the unweighted MD simulations, which explored a broader range of ribose conformations (Supplementary Fig. S23), reweighting with CCRs significantly refined the sampled populations, bringing them into closer agreement with experimental values. This result validates experimental prediction of average *P*-values.

Despite the extensive sampling present in the original MD trajectories, the raw populations displayed relatively high deviations from experiment, reflecting biases in the force field and sampling limitations. However, the BME reweighting process, which incorporated experimental CCRs, effectively corrected these discrepancies by suppressing unphysical populations and enhancing experimentally supported states. In most cases, the computed mean *P-*values (red dots) aligned well with experimental derived *P-*values for a single state (yellow triangles). Strikingly, for experimental *P-*values fitted to a two-state model, computed mean *P-*values appeared between the fitted experimental states, with the closer distance to the higher populated state. This demonstrates that CCR reweighting successfully captured the underlying conformational equilibria of the ribose conformations. While some minor deviations persisted, particularly in nucleotides where the raw MD data displayed excessive heterogeneity, the overall improvement suggests that CCR-guided reweighting provided a robust correction mechanism. This agreement reinforced the role of ribose conformational dynamics in governing RNA structural dynamics and highlights the utility of CCRs as sensitive reporters of local conformational preferences.

### The ostensible heterogeneity of s2m secondary structure models in literature

**Figure 10.**
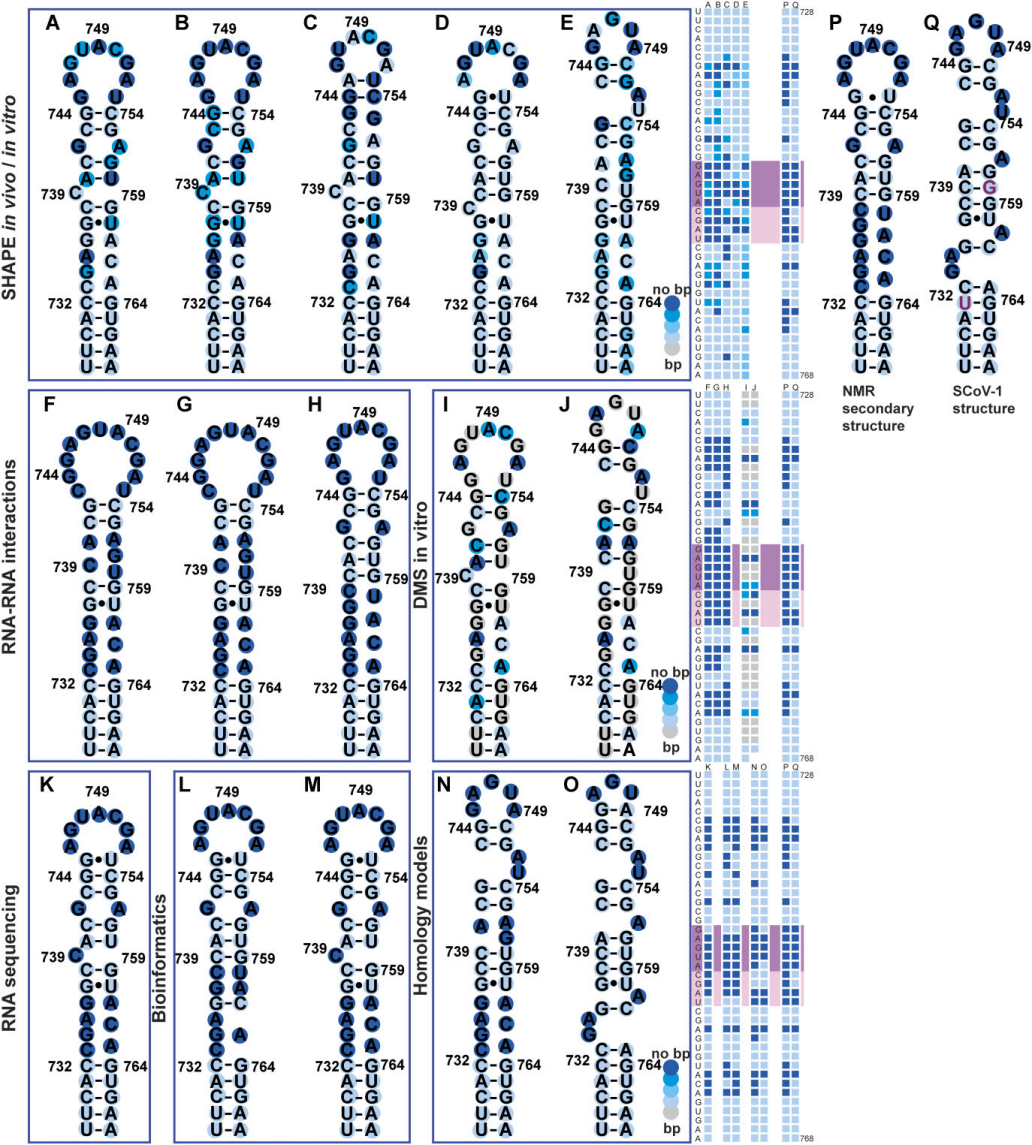
Summary of secondary structures of SCoV-2 Wuhan s2m with experimental depiction of their base pair probability that were reported before. The experimental data include SHAPE *in vivo* or *in vitro*, RNA–RNA interaction profiles, DMS *in vitro*, and RNA sequencing. The structures were combined to be represented using a colour code from light blue (base pair) to dark blue (not base paired) overlayed in their respective secondary structures as well as in table form for better visualization. In dark magenta pentaloop nucleotides are highlighted in the table, light magenta highlights the nonaloop nucleotides. (**A**) Manfredonia *et al. in vitro* [27]. (**B**) Manfredonia *et al. in vivo* [27]. (**C**) Huston *et al. in vivo* [28]. (**D**) Sun *et al. in vivo*/ *in vitro* [29]. (**E**) Lulla *et al. in vitro* [30]. (**F**) Ziv *et al.* [31]. (**G**) Zhang *et al.* A [32]. (**H**) Zhang *et al.* B [32]. (**I**) Manfredonia *et al.* DMS *in vitro* [27]. (**J**) Morandi *et al. in vitro* [33]. (**K**) Cao *et al.* [26]. (**L**) Ahmed *et al.* [25]. (**M**) Rangan *et al.* [21]. (**N**) Tengs *et al.* [14]. (**O**) Aldhumani *et al.* [24]. (**P**) Secondary structure of SCoV-2 Wuhan s2m determined by NMR by Wacker, Weigand *et al.* [9]. (**Q**) Secondary structure of SCoV-1 s2m [16].

A large number of different proposed secondary structures of SCoV-2 Wuhan s2m (wt SCoV-2) have been published to date (Fig. 10) [14, 21, 24–33]. The comparison of s2m secondary structures has been conducted previously, but not yet set in context with new experimental data [20]. We here comprehensively compare the 15 available secondary structures of s2m to our NMR structure and provide evidence for the significance of the proper secondary structure for a process of the viral life cycle.

In several cases, the proposed secondary structure models differed significantly in the annotation of base pairings (Table 1). Homology-based or bioinformatics approaches have frequently used the X-ray structure of SCoV-1 s2m as a basis to determine the influence of the mutations in SCoV-2 and stated no significant change in secondary structure between SCoV-1 and SCoV-2 [14, 24]. These structures predicted a pentaloop like in SCoV-1, which is a rather stable structural motif. By stark contrast, especially the *in vivo* methods showed a more dynamic loop with a higher number of unpaired nucleotides [32].

**Table 1.** Number of nucleotides in loop, internal loop, in Watson–Crick (WC) base pairs or non-WC base pairs (NWC) of published secondary structures of SCoV-2 Wuhan s2m from Fig. 10. (A) Manfredonia *et al. in vitro* [27]. (B) Manfredonia *et al. in vivo* [27]. (C) Huston *et al. in vivo* [28]. (D) Sun *et al. in vivo*/ *in vitro* [29]. (E) Lulla *et al. in vitro* [30]. (F) Ziv *et al.* [31]. (G) Zhang *et al.* A [32]. (H) Zhang *et al.* B [32]. (I) Manfredonia *et al.* DMS *in vitro* [27]. (J) Morandi *et al. in vitro* [33]. (K) Cao *et al.* [26]. (L) Ahmed *et al.* [25]. (M) Rangan *et al.* [21]. (N) Tengs *et al.* [14]. (O) Aldhumani *et al.* [24]. (P) Secondary structure of SCoV-2 Wuhan s2m determined by NMR by Wacker, Weigand *et al.* [9]. (Q) Secondary structure of SCoV-1 s2m [16]. A–E: SHAPE *in vivo*/*in vitro*. F-H: RNA–RNA interactions. I-J: DMS *in vitro*. K: RNA sequencing. L-M: Bioinformatics. N-O: Homology models. P: NMR, Q: SCoV-1 X-ray

Structures	A	B	C	D	E	F	G	H	I	J	K	L	M	N	O	P	Q
*Loop*	*9*	*9*	*6*	*7*	*5*	*11*	*11*	*9*	*9*	*5*	*7*	*7*	*7*	*5*	*3*	*7 (9)*	*5*
*Bulged*	*3*	*3*	*6*	*3*	*7*	*5*	*5*	*2*	*3*	*5*	*3*	*2*	*3*	*5*	*3*	*2*	*3*
*Internal Loop*	*7*	*7*	*7*	*7*	*7*	*7*	*7*	*10*	*7*	*7*	*7*	*8*	*7*	*7*	*3*	*10*	*3*
*WC*	*20*	*20*	*20*	*20*	*20*	*16*	*16*	*20*	*20*	*22*	*20*	*22*	*20*	*22*	*28*	*20*	*28*
*NWC*	*2*	*2*	*2*	*4*	*2*	*2*	*2*	*0*	*2*	*2*	*4*	*2*	*4*	*2*	*4*	*2 (1)*	*2*
*Sum*	*41*	*41*	*41*	*41*	*41*	*41*	*41*	*41*	*41*	*41*	*41*	*41*	*41*	*41*	*41*	*41*	*41*

Most of these experimentally determined secondary structures contained a loop with more than five nucleotides. The SCoV-1 s2m has a stable pentaloop, while s2m of SCoV-2 has a very dynamic nonaloop. The dynamic architecture of the nonaloop was retained for wt SCoV-2 s2m, SCoV-2 s2m Delta, and SCoV-2 s2m Delta_short, as evidenced by the high conservation of aromatic C6H6/C8H8 and ribose C1’H1’ NMR signals that reliably sense changes in RNA structure [111] (Supplementary Fig. S24).

The comparison of secondary structure models depicted in Fig. 10 shows that the hepta-/nonaloop represented with or without transient G-U base pair was present in A), B), D), H), I), K), L), and M), collectively adding up to only 8 out of 15 structure models. Strikingly, all other secondary structures of SCoV-2 s2m suggested a deviating loop size. For example, Lulla *et al.* [30] showed SHAPE activity at nucleotides C750 and G751 but still depicted those nucleotides in base pairs with C743 and G744 (Fig. 10 E). Yet, reactivity data from experimental *in vivo* methods were in strong agreement with our secondary structure, indicating that the discrepancy could not be attributed to the *in vitro* characteristic of the NMR method. In summary, we conclude that the evolutionary differences between the SCoV-1 and SCoV-2 sequences altered the secondary structure of the s2m.

To demonstrate the importance of a correct secondary structure in the context of the interpretation of biological or biochemical data, such as interpreting the accessibility of residues, we investigated Nsp15 cleavage of our Delta s2m constructs.

### Nsp15 cleaves both s2m Delta and s2m Delta_short

**Figure 11.**
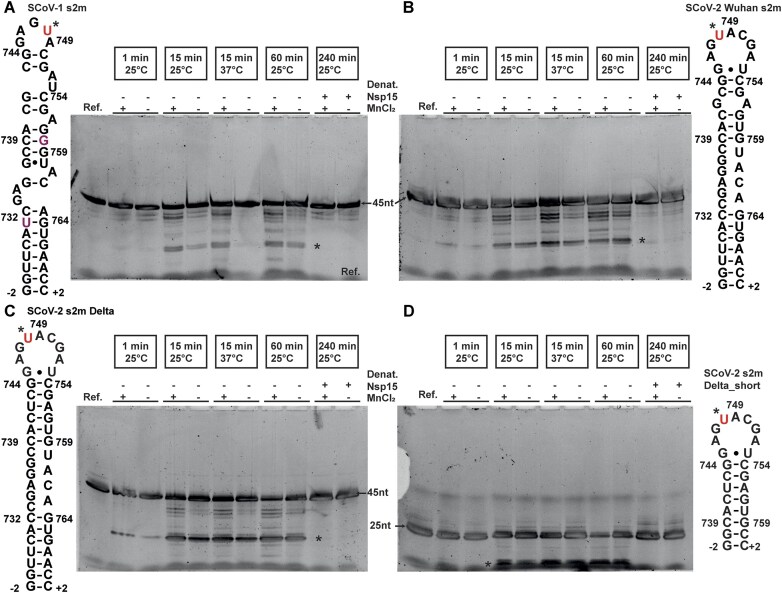
Cleavage assay with Nsp15 and (**A**) SCoV-1 s2m, (**B**) SCoV-2 Wuhan s2m, (**C**) SCoV-2 s2m Delta, and (**D**) SCoV-2 s2m Delta_short. A schematic representation of the secondary structure of each construct can be seen next to the respective PAA gel. Nucleotides marked with red correspond to most probable cleavage sites. *: Cleavage products and position in RNAs.

Nsp15 is a uridine-specific viral endonuclease involved in immune system evasion by cleaving dsRNA at unpaired uridines [112, 113]. Delineation of both base paired and nonbase paired regions of viral RNA is thus crucial to interpret the interaction profile of a target RNA to viral and host factors and understand the role of the conserved and mobile element s2m. While others previously reported Nsp15 cleavage for SCoV-1 s2m [34], no such cleavage assays have yet been reported for Wuhan s2m and s2m Delta. Here, the Nsp15 cleavage pattern of several s2m constructs in comparison to SCoV-1 s2m was investigated via denaturing gel electrophoresis. After reproducing the results for the SCoV-1 s2m construct, we conducted these experiments for SCoV-2 Wuhan s2m [34], s2m Delta, and s2m Delta_short and observed that all RNA constructs underwent cleavage by Nsp15 (Fig. 11). Interestingly, the s2m Delta_short RNA showed one additional fragment in addition to its full-length band, which originated from the cleaved s2m Delta_short at the unpaired U748 position in the loop (*). Our data show that SCoV-2 s2m was indeed cleaved differently than postulated, which is why secondary structure predictions without strong experimental evidence should be viewed with caution.

## Conclusion

We report an integrated NMR and MD study to characterise the structure and dynamic nature of the s2m apical loop in SCoV-2 Delta. The highly dynamic NMR derived structural ensemble of the apical stem loop in s2m Delta (s2m Delta_short) converges in the stem region with fraying at the stem closing base pairs and structural fluctuation in the apical loop. NMR structure determination for such dynamic systems has certain limitations with respect to accurately capturing all representative states of a conformational ensemble. Often, convergence of an ensemble is only achieved by overestimation of certain input constraints. In our case, for instance, all states of the NMR ensemble exhibit the G-U loop-closing base pair as a result of constraining it. While this G-U base pair is also present to some extent in the MD simulations, a significant number of states are partially open or show alternative interactions. Thus, MD simulations resolved artificial convergence forced into NMR structures by potential over-restraining.

With the CSs obtained via this integrative MD and NMR approach, we amplified the structural space of a nonaloop with a transient G-U base pair. In the ribosome and the newly determined ROOL RNA [114] we found highly structured G-N7-U or U-N7-G representatives of nonaloop that seem to adopt a specific structure. However, this shows that the nonaloop is a popular structure-generating motif for higher-order structures and that so far only very structured members of this motif family are available.

The reweighted MD simulation revealed the increased sampling of a flipped back conformation of the nonaloop, which was not as prominent in unweighted MD. Such a highly stabilised flipped back conformation was reported for SCoV-1. Even though the nucleotide mutation from SCoV-1 to SCoV-2 disrupted the secondary structure, the unique tertiary structure of SCoV-1 in the apical loop has been found in the nonaloop of s2m as well, though not exclusively populated as seen by the dynamic features in this RNA from this study, interacting partners might sample such conformations for interactions. The structural diversity is likely to be exploited in molecular recognition events.

The structural dynamics of s2m is impressively demonstrated by the plethora of ambiguous secondary structures published for s2m (Fig. 10). The different reactivity profiles observed for the apical nucleotides can be explained by the dynamic properties exhibited in these nucleotides. Resulting secondary structure models are affected by the bias introduced to chemical probing due to blind spots on the one hand and the dependence on the underlying prediction model on the other hand. In contrast, NMR is unique in detecting both structurally homogeneous and heterogeneous ensembles, and in its ability to clearly distinguish between these cases. Additionally, NMR is a noninvasive method, whereas probing irreversibly influences conformational equilibria (119). MD becomes useful as soon as NMR data predict structural heterogeneity on a fast (∼ns) time scale, then the accuracy of the ensemble can be significantly improved by finding physically meaningful states that fit the experimental data.

In conclusion, the loop of s2m in SCoV-2 does not adopt a single structure but is characterised by a base pair- and stack-reshuffled ensemble via a combination of NMR and MD simulations. The flexibility and dynamic nature of this RNA supported by our hetNOE data could be a hint at the function, as flexible regions are considered to be important regions for interactions with ligands or other biomolecules. Structural flexibility could be a prerequisite for selective recognition, induced fit or the capturing of interaction partners. It can also facilitate a dual function that requires the adoption of distinct structures.

## Supplementary Material

gkaf552_Supplemental_Files

## Data Availability

Plasmid vector cards can be obtained upon request. All data described in this article is available in figures, tables, and Supplementary Data. Chemical shift assignment of s2m Delta_short is available at BMRB-ID: 34919. The unmodified structural ensemble (20 models) can be found in the PDB with the ID: 9FM4. The sequence numbers in the depositions correspond to the position in the viral genome -29000 including the three mutations C738G, U760C, and A761C that are necessary to establish the two G-C closing base pairs. Nucleotides G-2 and G-1 correspond to nucleotides G737 and G738. Nucleotides C + 2 and C + 1 correspond to C760 and C761. SAXS raw data were deposited in the SASBDB at SASDUD9 (1.5 mg/ml) and SASDXB3 (2.5 mg/ml). All raw NMR data as well as analysis of data and the MD centroids of CCR-reweighted CS can be found in the GUDE repository of the Goethe University Frankfurt: DOI: 10.25716/gude.0pdz-ar7h
