# Supplementary material for: Structural heterogeneity and dynamics in the apical stem loop of s2m from SARS-CoV-2 Delta by an integrative NMR spectroscopy and MD simulation approach
Source: Nucleic Acids Res. 2025 Jun 30;53(12):gkaf552. doi: 10.1093/nar/gkaf552 (PMC12207407; doi:10.1093/nar/gkaf552)
Supplement: gkaf552_Supplemental_Files [file gkaf552_supplemental_files.zip › s2m_Delta_nonaloop_Resubmission_SI_final_v2.pdf]

SI

Structural heterogeneity and dynamics in the apical stem loop of s2m from SARS-CoV-2 Delta by an integrative NMR spectroscopy and MD simulation approach

Maria A. Wirtz Martin[1,†], Joseph A. Makowski[2,†], Tobias Matzel[1], Adam H. Kensinger[2], Alexander Herr[1], Christian Richter[1], Hendrik R. A. Jonker[1], Anna Wacker[1,*], Jeffrey D. Evanseck[2,*], and Harald Schwalbe[1,*]

[1]Institute for Organic Chemistry and Chemical Biology, Center for Biomolecular Magnetic Resonance (BMRZ), Goethe University Frankfurt, Max-von-Laue-Str. 7, 60438 Frankfurt, Germany

[2]Department of Chemistry and Biochemistry and Center for Computational Sciences, Duquesne University, Pittsburgh, Pennsylvania 15282, United States.

† Authors contributed equally.

* To whom correspondence should be addressed. Email: schwalbe@nmr.uni-frankfurt.de Correspondence may also be addressed to: wacker@nmr.uni-frankfurt.de, evanseck@duq.edu

Keywords: s2m, Covid-19, NMR Assignment, RNA structure, NMR dynamics, Molecular dynamics simulation

SUPPLEMENTARY FIGURES

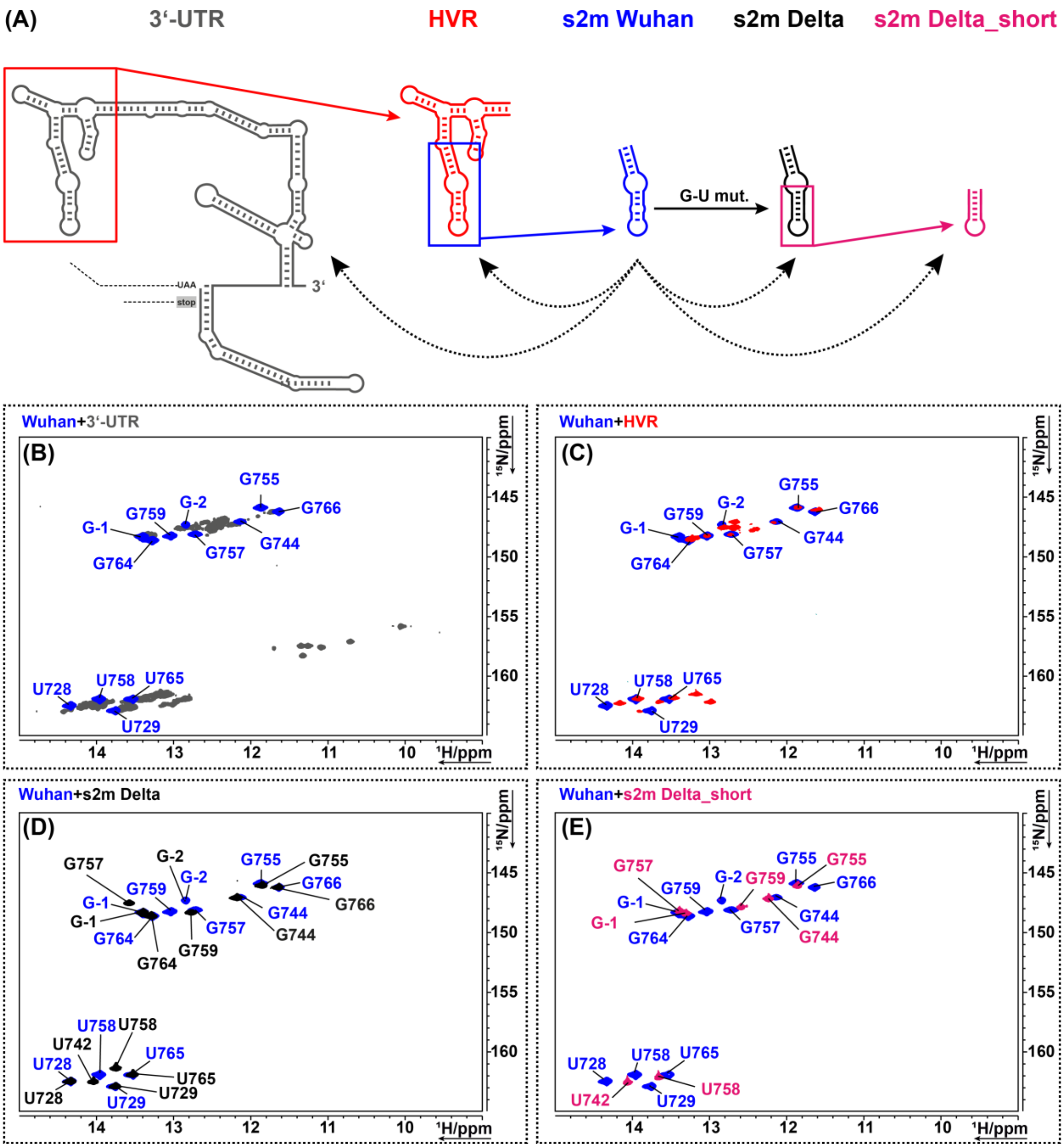

Supplementary Figure 1. Overlay of imino resonance signals of Wuhan s2m with 3′-UTR, HVR, s2m Delta and s2m Delta_short. (A) Schematic depiction of Wuhan s2m (blue) in SCoV-2 in the context of 3′-UTR (grey), HVR (hypervariable region; red) and transition from Wuhan s2m to s2m Delta (black) and s2m Delta_short (magenta). (B) ^{1}H, ^{15}N BEST-TROSY overlay of imino signal regions of Wuhan s2m (blue, sample#1) and 3′-UTR (grey, sample#8). (C) ^{1}H, ^{15}N BEST-TROSY overlay of imino signal regions of Wuhan s2m (blue, sample#1) and HVR (red, sample#9). (D) ^{1}H, ^{15}N BEST-TROSY overlay of imino signal regions of Wuhan s2m (blue, sample#1) and s2m Delta (black, sample#2). (E) ^{1}H, ^{15}N BEST-TROSY overlay of imino signal regions of Wuhan s2m (blue, sample#1) and s2m Delta_short (magenta, sample#3).

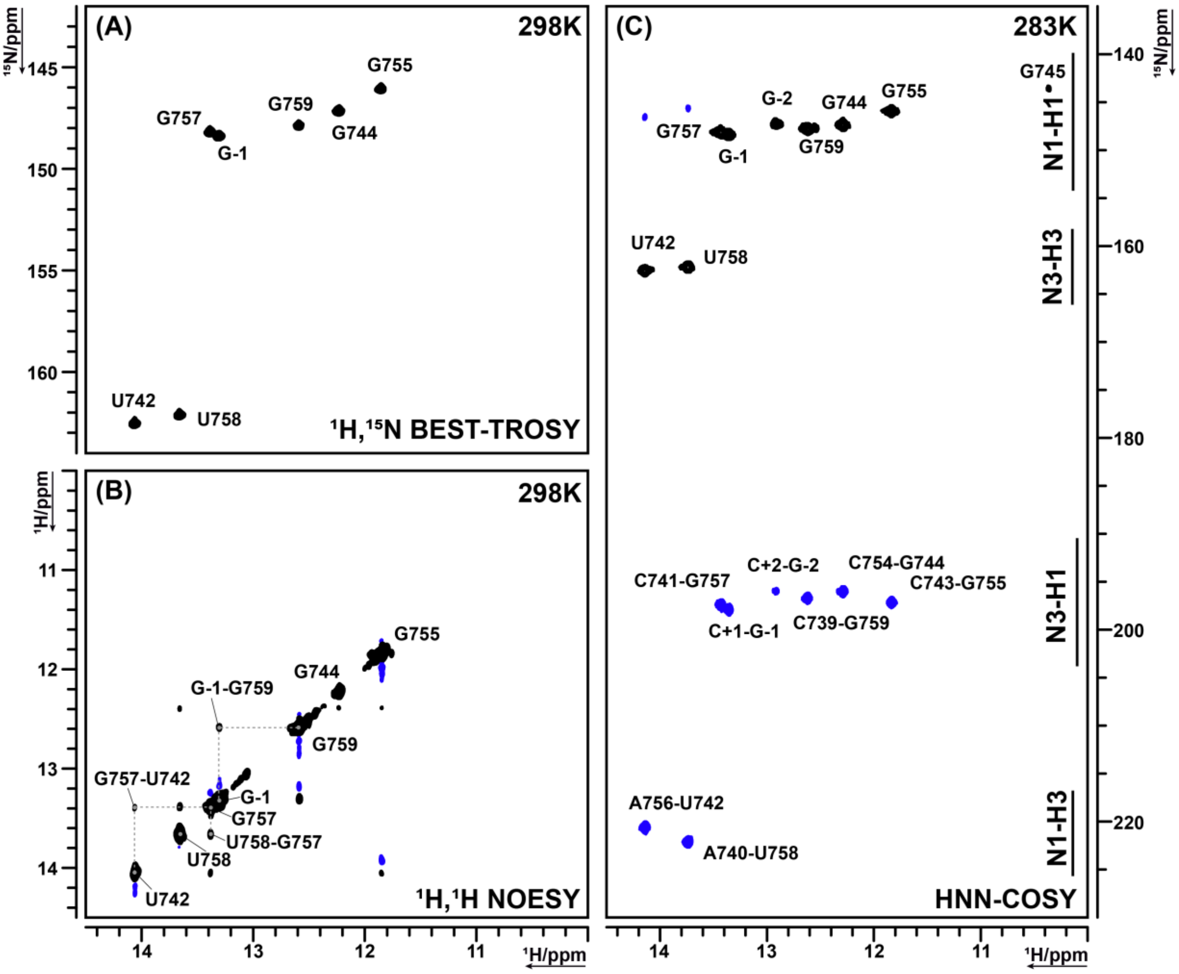

Supplementary Figure 2. Imino proton assignment of s2m Delta_short with ¹H, ¹H NOESY, ¹H, ¹⁵N BEST-TROSY at 298 K and HNN-COSY at 283 K. (A) ¹H, ¹⁵N BEST-TROSY of imino signal region s2m Delta_short. (B) ¹H, ¹H NOESY spectrum of the imino proton region. Peaks with negative intensity coloured in blue. (C) HNN-COSY spectrum of s2m Delta_short at 283 K. All spectra recorded with sample#3.

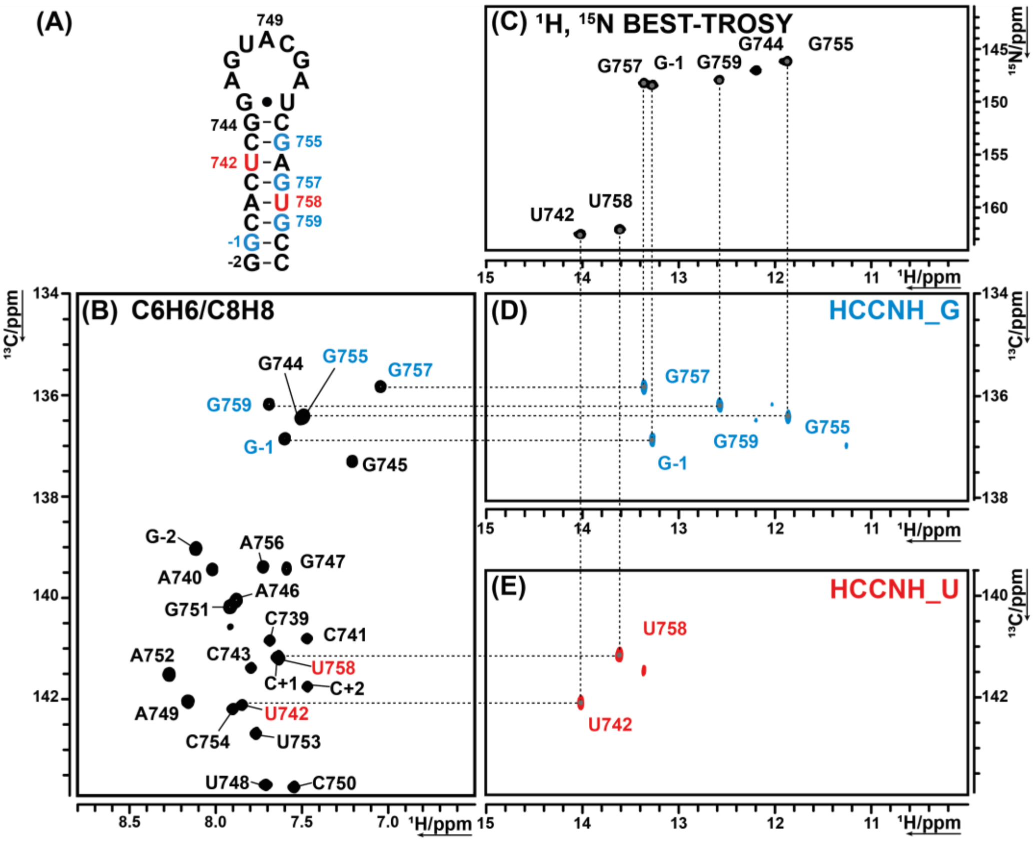

Supplementary Figure 3. HCCNH assignment of s2m Delta_short with ^{1}H, ^{13}C-HSQC at 308 K. (A) Secondary structure of s2m Delta_short RNA. Assigned nucleotides in the stem region marked in blue (G) and red (U). (B) ^{1}H, ^{13}C HSQC spectrum of aromatic protons (C6H6/C8H8). (C) ^{1}H, ^{15}N BEST-TROSY of imino signal region of s2m Delta_short. (D) HCCNH spectrum of s2m Delta_short referenced for G nucleotides ($1J_{CH}$(aro)= 216 Hz) $1J_{CH}/2$ was added to ^{13}C chemical shift (+108 Hz). (E) HCCNH spectrum of s2m Delta_short referenced for U nucleotides ($1J_{CH}$(aro)= 184 Hz) $1J_{CH}/2$ was added to ^{13}C chemical shift (+92 Hz). All spectra were recorded with sample#3.

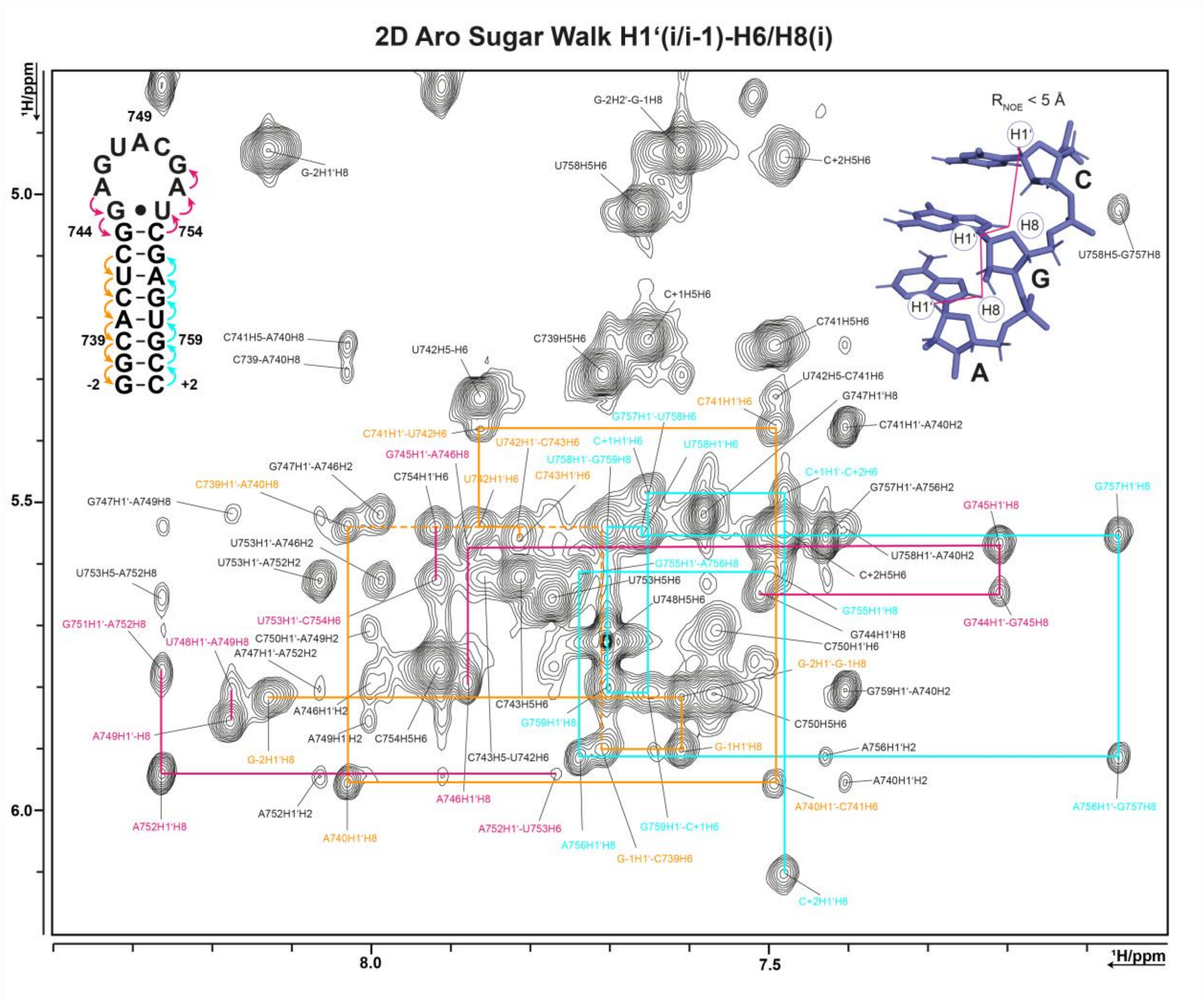

Supplementary Figure 4. 2D ¹H, ¹H NOESY of s2m Delta_short (sample#4.5) at 308 K. The sequential H1′ to H8 walk from C+2- G755 is highlighted in light blue, from C743-G-2 is highlighted with orange lines and in pink lines are NOE connectivities highlighted between H1′ and H8 protons of loop nucleotides. Severe signal overlap in the region of w1=8.0-7.5 ppm and w2=5.0-6.0 ppm prevented the further assignment of NOE connectivities in the loop region.

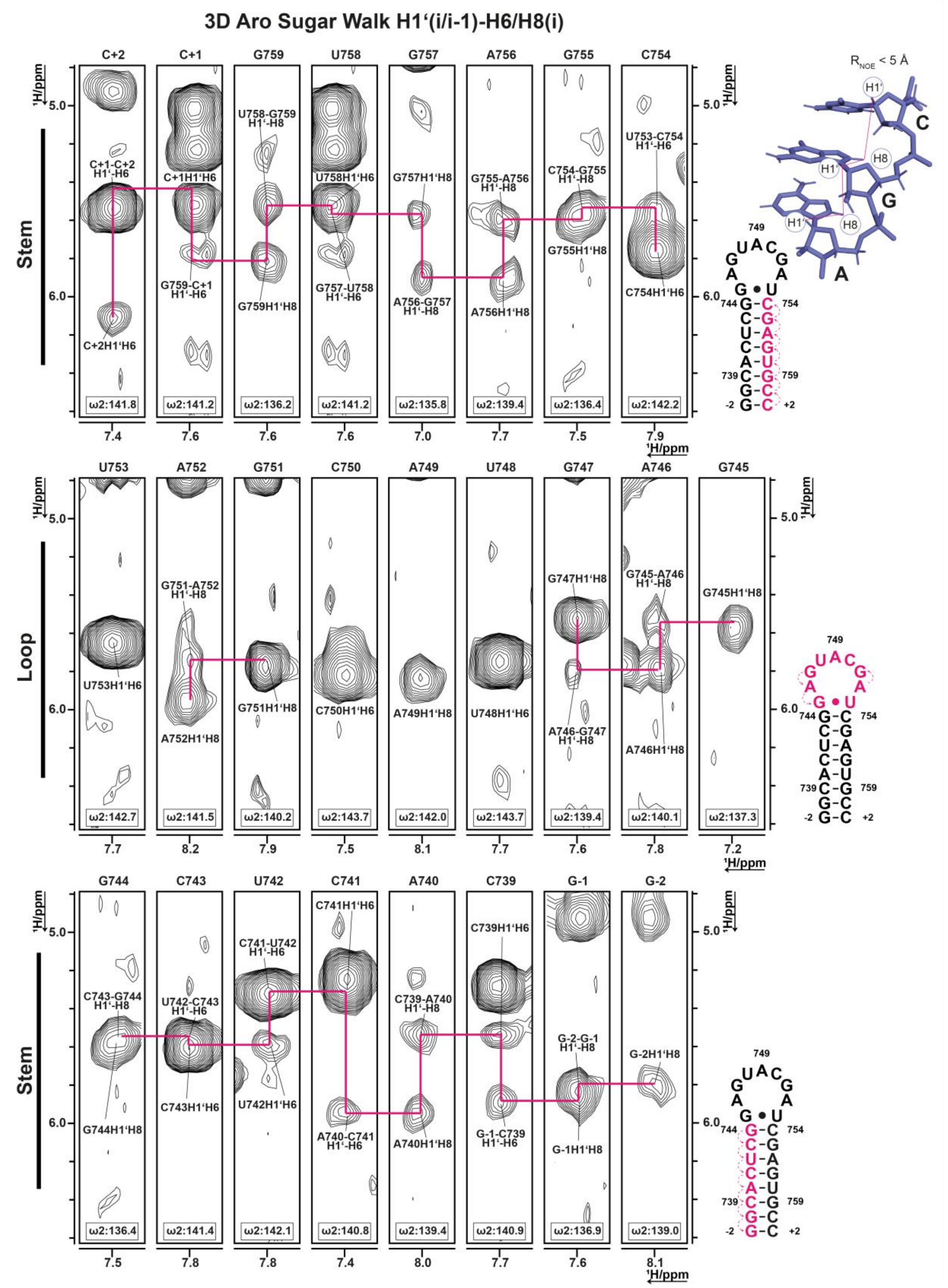

Supplementary Figure 5. **3D ¹H, ¹H NOESY-HSQC of s2m Delta_short (sample#3) at 308 K.** For each nucleotide the ¹H, ¹H strip is depicted. Low signal resolution and signal overlap resulted in no visible cross peaks between nucleotides in the loop region and lead to an interruption in the sequential walk.

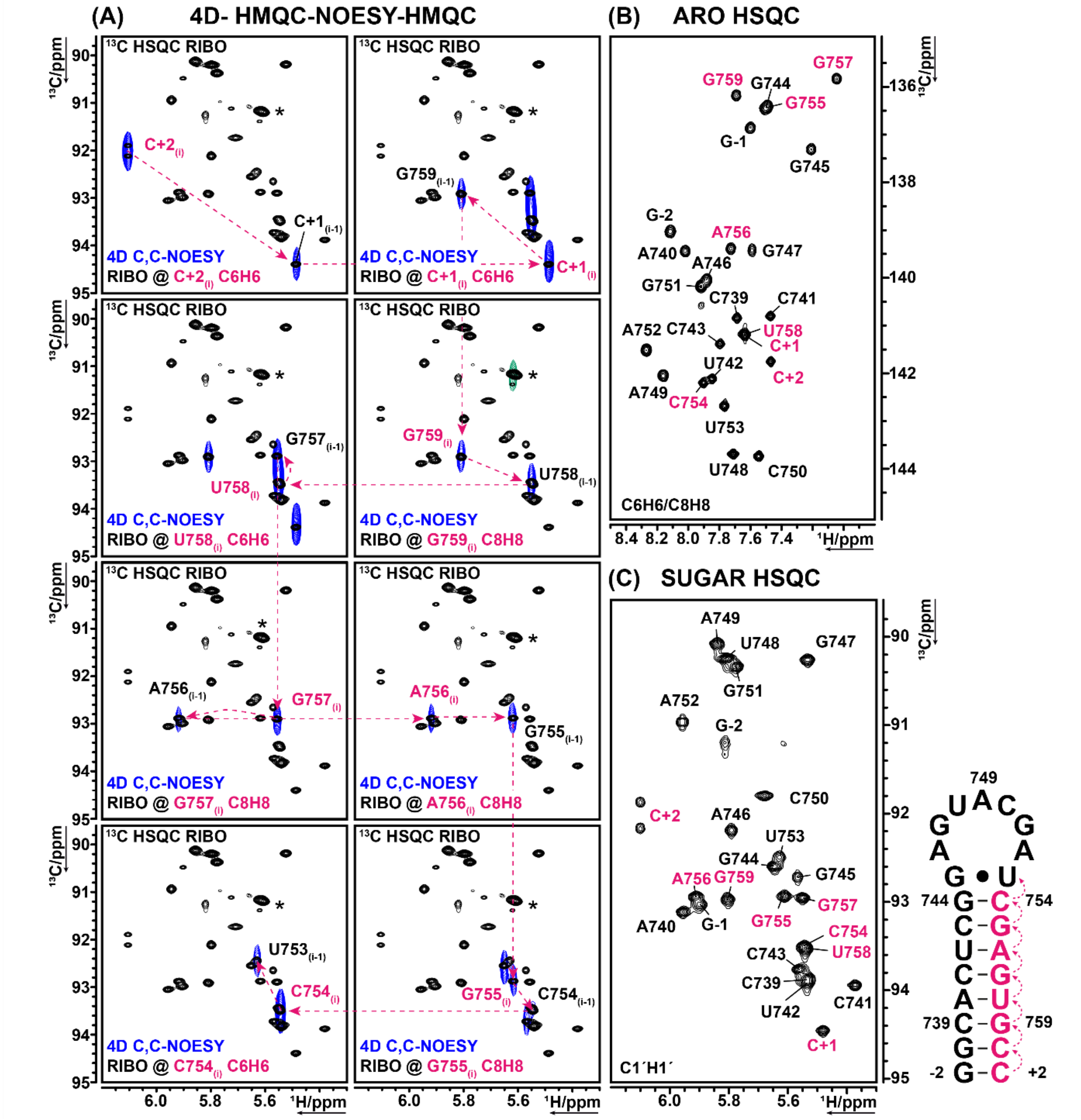

Supplementary Figure 6. 4D HMQC-NOESY-HMQC planes of nucleotides C754-C+2 (pink) in s2m Delta_short. (A) Overlay of the 4D HMQC-NOESY-HMQC spectrum (blue, sample#4, Supplementary Table 1) and ^{1}H, ^{13}C-HSQC spectrum (black, sample#4, Supplementary Table 1). By sequentially sorting the 2D planes of the 4D in the C1′H1′ dimension a NOESY walk is possible. Starting from an aromatic signal of a specific nucleotide (i) peaks are visible for the C1′H1′ resonance corresponding to the same nucleotide and to the previous nucleotide (i-1). By arranging the planes from 3′ to 5′ direction, a sequential assignment was achieved. Both spectra were recorded at 308 K. The resonance assignment of all nucleotides is annotated and the C6H6/C8H8 aromatic chemical shift leads to the shown planes. The respective nucleotide for that plane (pink) is annotated in the lower left corner of each spectrum. Degradation peaks are visible of s2m Delta_short from sample#4 (*). (B) Aromatic ^{1}H,^{13}C-HSQC at 308 K (sample#3, Supplementary Table 1). (C) C1′H1′ region of s2m Delta_short at 308 K (sample#3, Supplementary Table 1).

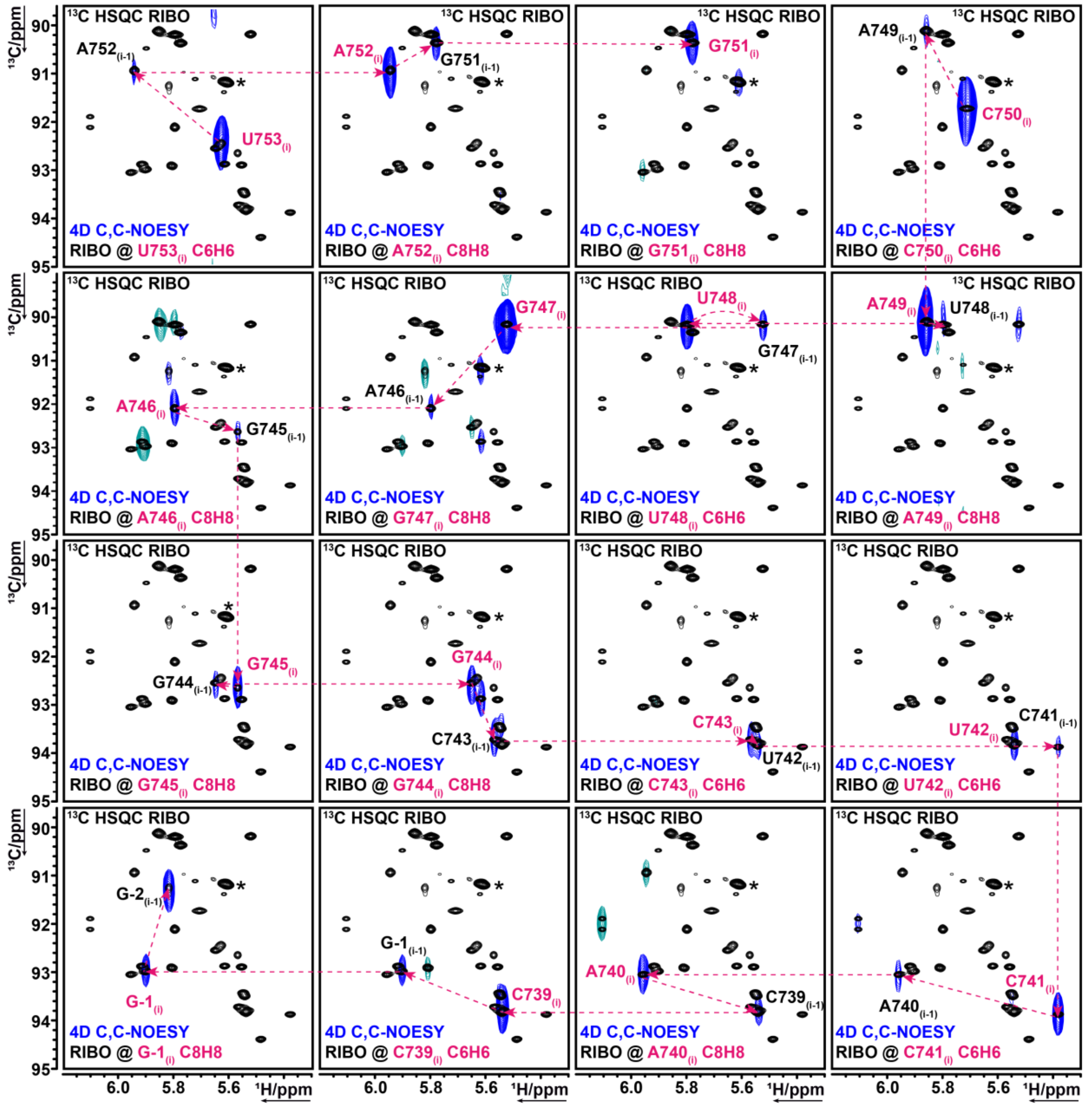

Supplementary Figure 7. 4D HMQC-NOESY-HMQC planes of nucleotides G-2-U753 in s2m Delta_short RNA. Overlay of the 4D HMQC-NOESY-HMQC spectrum (blue, sample#4, Supplementary Table 1) and ^{1}H, ^{13}C-HSQC spectrum (black, sample#4, Supplementary Table 1). By sequentially sorting the 2D planes of the 4D in the C1′H1′ dimension a NOESY walk is possible. Starting from an aromatic signal of a specific nucleotide (i) peaks are visible for the C1′H1′ resonance corresponding to the same nucleotide and to the previous nucleotide (i-1). By arranging the planes from 3′ to 5′ direction, a sequential assignment was achieved.Both spectra were recorded at 308 K. The resonance assignment of all nucleotides is annotated and the C6H6/C8H8 aromatic chemical shift leads to the shown planes. The respective nucleotide for that plane (pink) is annotated in the lower left corner of each spectrum. Degradation peaks are visible of s2m Delta_short from sample#4 (*).

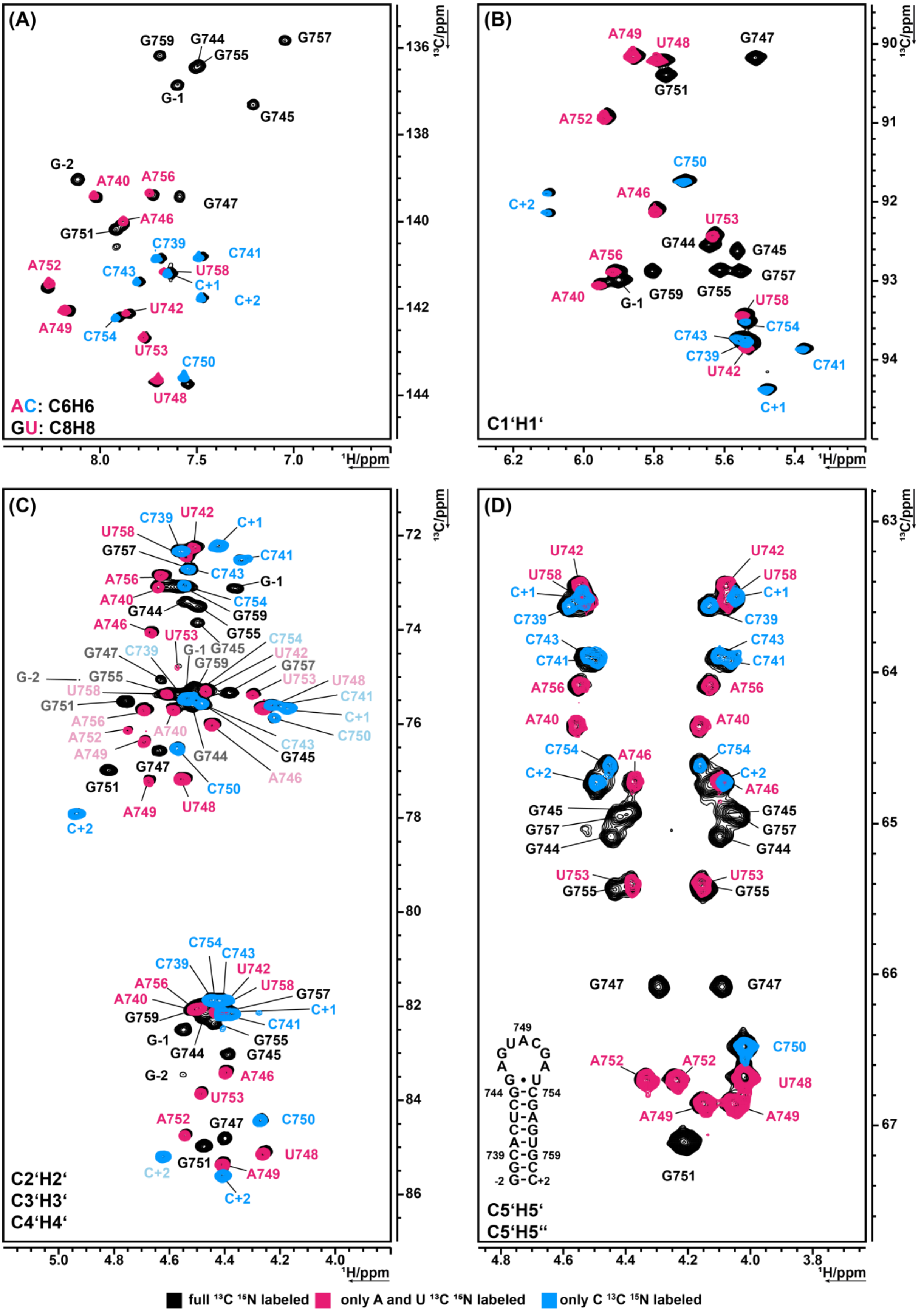

Supplementary Figure 8. Chemical shifts of C1'H1'-C5'-H5'' of s2m Delta_short RNA. . (A) Assignment of aromatic chemical shifts (AG: C8H8 and UC: C6H6) of sample#4.5, sample#5, sample#6. (B) Assignment of C1'H1' chemical shifts of sample#4.5, sample#5, sample#6. (C) Assignment of C2'H2' (lighter colour) and C3'H3' (saturated colour) chemical shifts of sample#4.5, sample#5, sample#6. (D) Assignment of C5'H5' and C5'H5''chemical shifts of sample#4.5, sample#5, sample#6. All spectra were recorded at 308 K

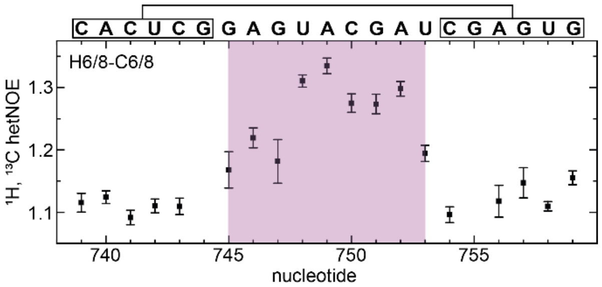

Supplementary Figure 9. ^{1}H, ^{13}C heteronuclear NOE of s2m Delta_short for aromatic C6H6/C8H8 resonances (sample#4.5). The RNA sequence is given on the top of the plot with squares indicating base-paired regions. Loop nucleotides are highlighted with a pink box. HetNOE values were calculated based on signal intensities and errors were calculated based on signal/noise ratios for each peak. Spectra with and without NOE were recorded interleaved as pseudo 3Ds, with a relaxation delay of 5 s and a presaturation delay of 3 s. For temperature compensation an off-resonance pulse was applied at -1000 ppm in the reference experiment.

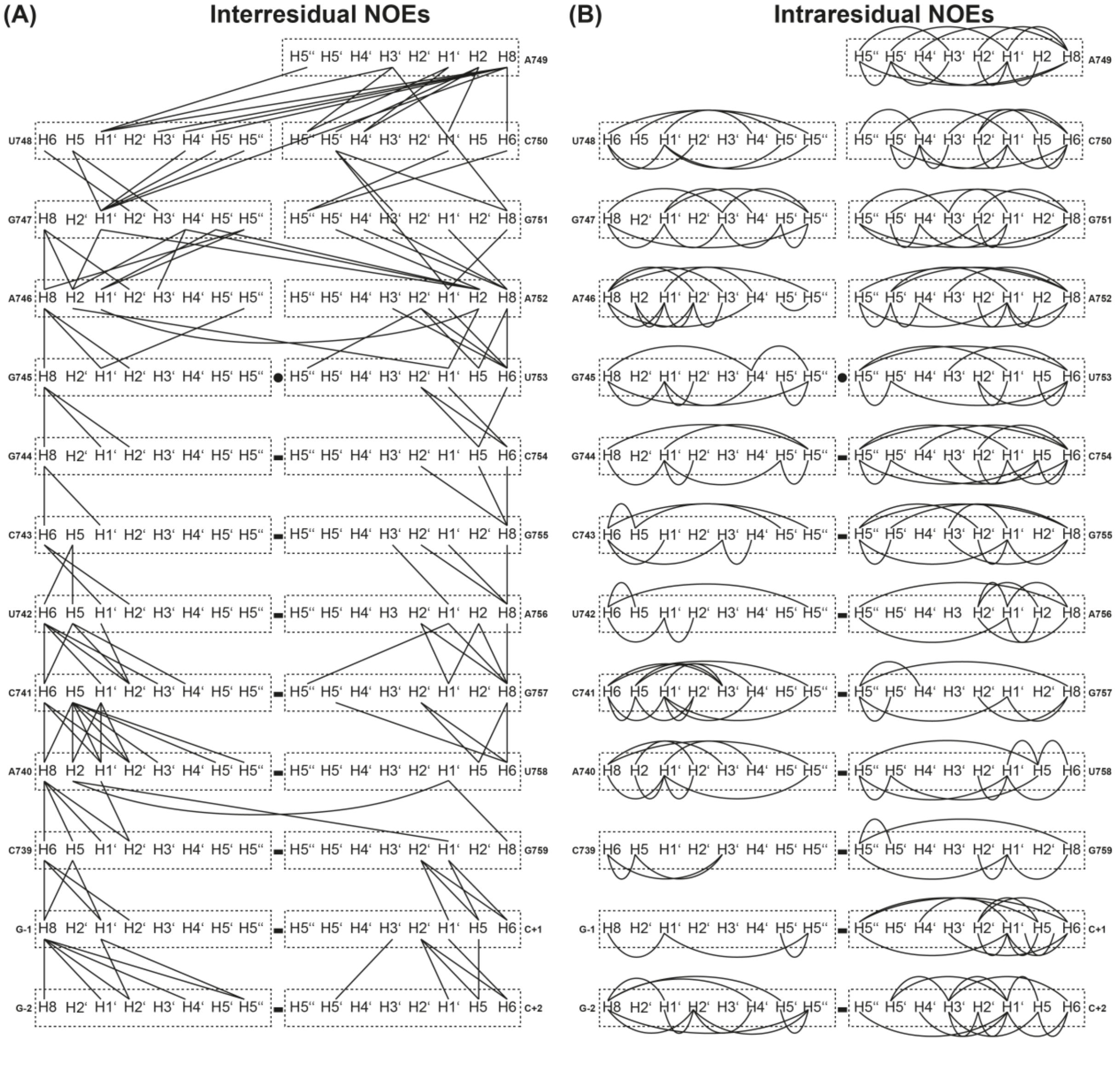

Supplementary Figure 10. (A) Inter-and (B) intraresidual NOE cross peaks from NOESY ^{1}H, ^{1}H spectrum at 150 ms mixing time.

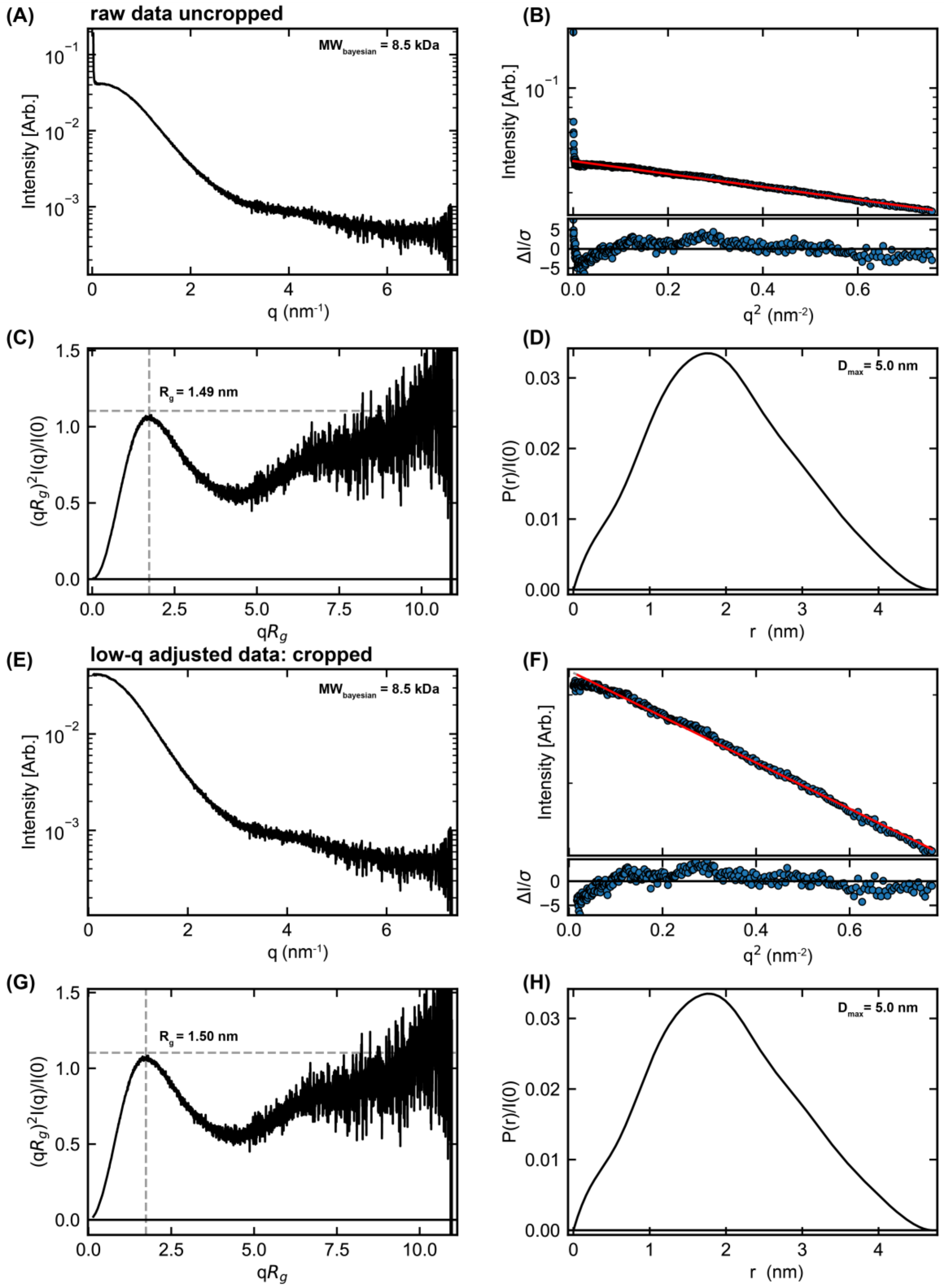

Supplementary Figure 11. Analysis of SAXS data for s2m Delta_short (1.5 mg/mL). (A-G) raw data, (E-H) low q adjusted data (cropped). (A, E) Scattering profiles on a log-lin scale. (B, F) Guinier fit (top) and fit residuals (bottom). (C, G) Dimensionless Kratky plot. Dashed lines show where a globular system would peak. (D, H) P(r) function normalised by I(0). Analysis was performed with BioXTAS RAW software.

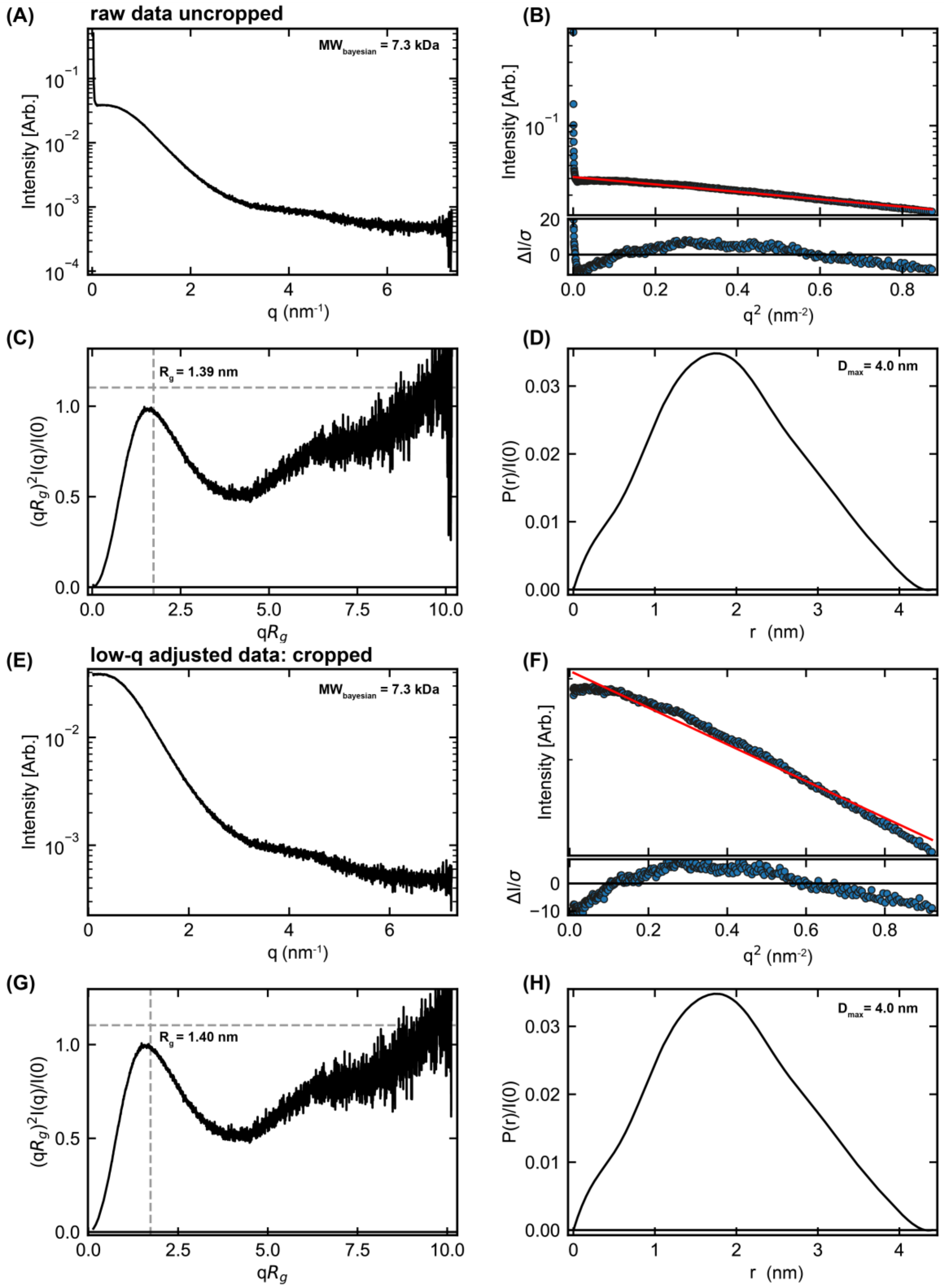

Supplementary Figure 12. Analysis of SAXS data for s2m Delta_short (2.5 mg/mL). (A-G) raw data, (E-H) low q adjusted data (cropped). (A, E) Scattering profiles on a log-lin scale. (B, F) Guinier fit (top) and fit residuals (bottom). (C, G) Dimensionless Kratky plot. Dashed lines show where a globular system would peak. (D, H) P(r) function normalised by I(0). Analysis was performed with BioXTAS RAW software.

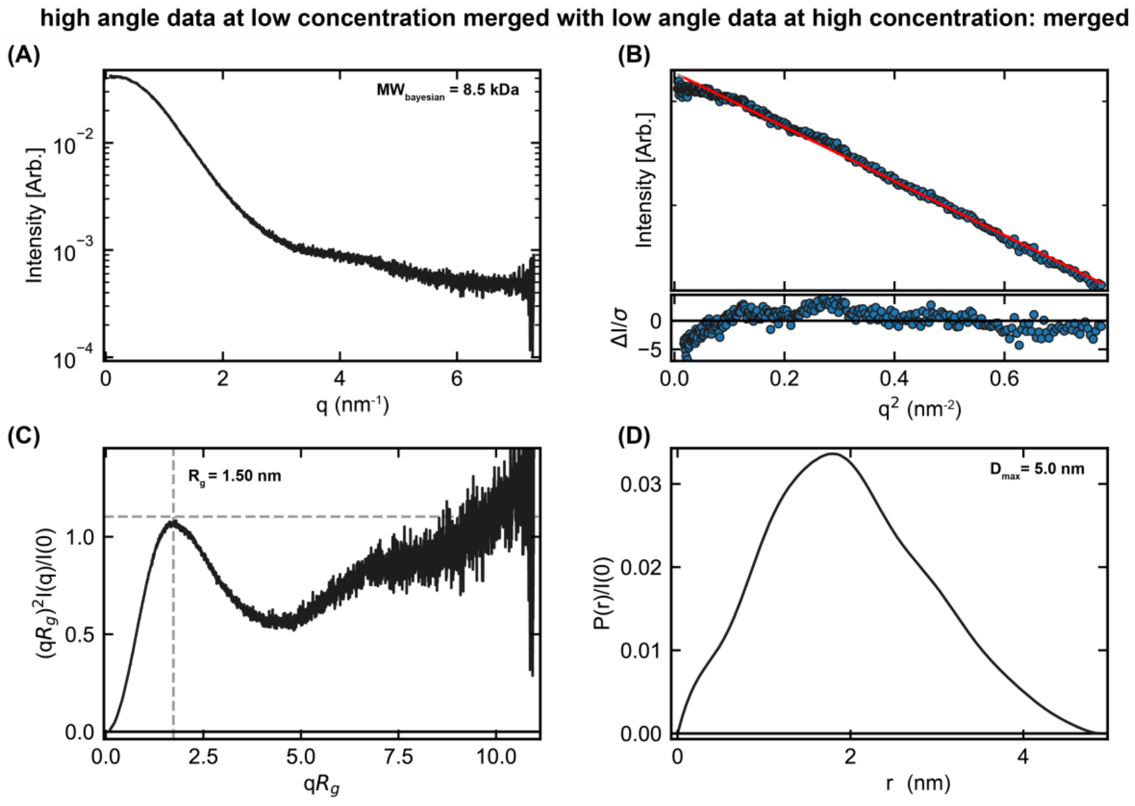

Supplementary Figure 13. Analysis of SAXS data for s2m Delta_short (merged). The first 500 points of high angle data at low concentration (1.5 mg/mL) were merged with low angle data at high concentration (2.5 mg/mL). (A) Scattering profiles on a log-lin scale. (B) Guinier fit (top) and fit residuals (bottom). (C) Dimensionless Kratky plot. Dashed lines show where a globular system would peak. (D) P(r) function normalised by I(0). Analysis was performed with BioXTAS RAW 2.3.0 software.

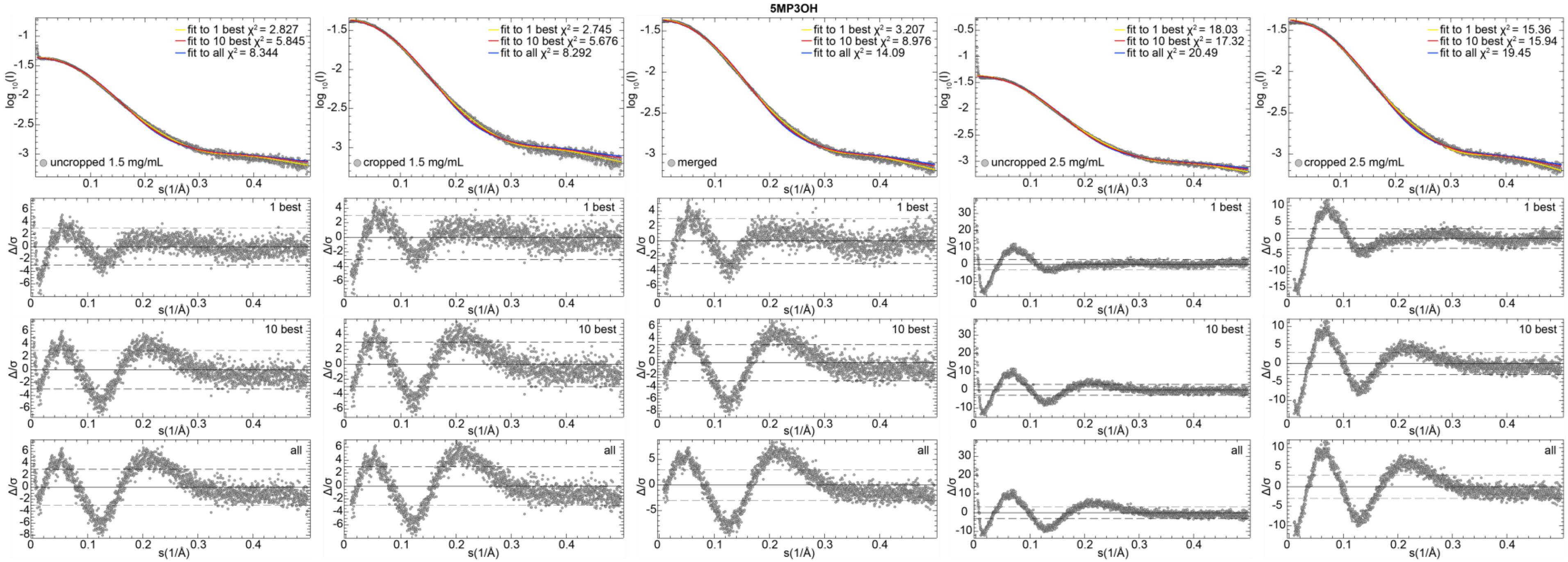

Supplementary Figure 14. CRYSOL fits and χ^2 values of SAXS data with unmodified structure models (5MP3OH) from ARIA NMR ensemble. (Top) Scattering profiles of SAXS data (grey dots). Solid lines show back calculated theoretical scattering curves from ARIA NMR ensemble models (yellow: fit to best model) (red: fit to 10 best ensemble (average)) (blue: fit to all (average). (Bottom) Individual residual fits of back calculated scattering data of models or ensembles to experimental SAXS data. Analysis was performed with ATSAS 4.0.0-2.

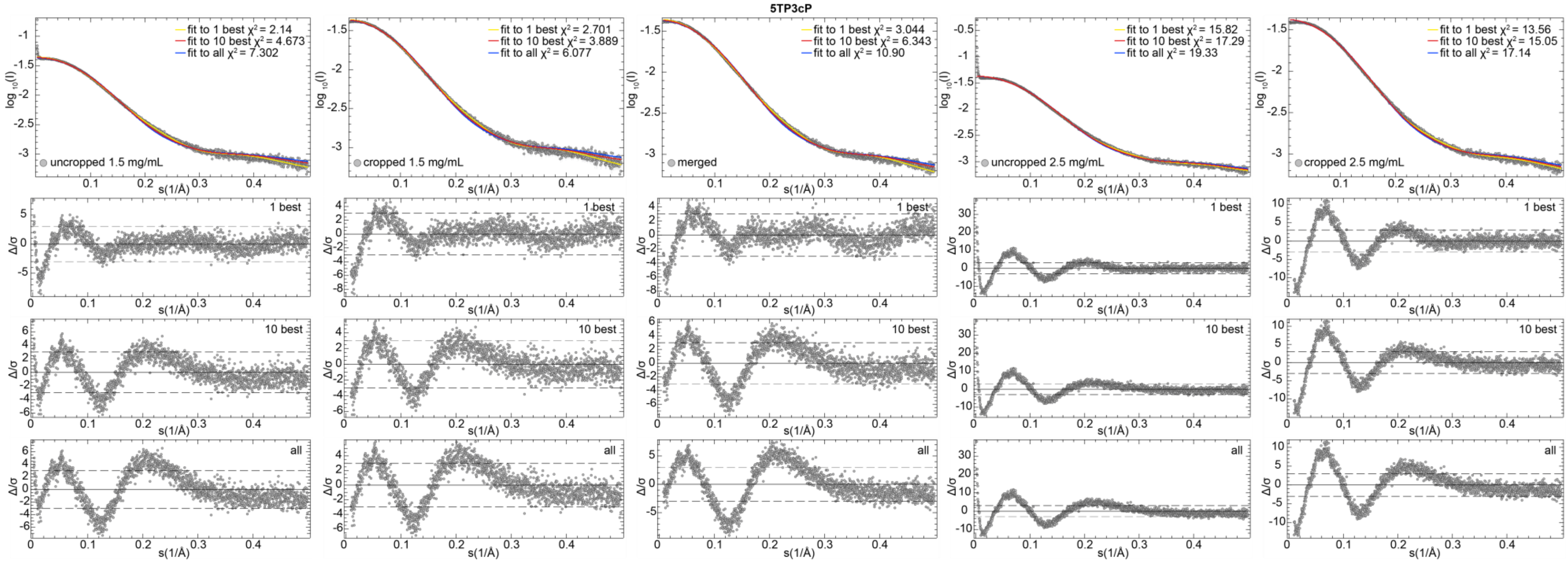

Supplementary Figure 15. CRYSOL fits and χ^2 values of SAXS data with modified structure models (5TP3cP) from ARIA NMR ensemble. (Top) Scattering profiles of SAXS data (grey dots). Solid lines show back calculated theoretical scattering curves from ARIA NMR ensemble models (yellow: fit to best model) (red: fit to 10 best ensemble (average)) (blue: fit to all (average). (Bottom) Individual residual fits of back calculated scattering data of models or ensembles to experimental SAXS data. Analysis was performed with ATSAS 4.0.0-2.

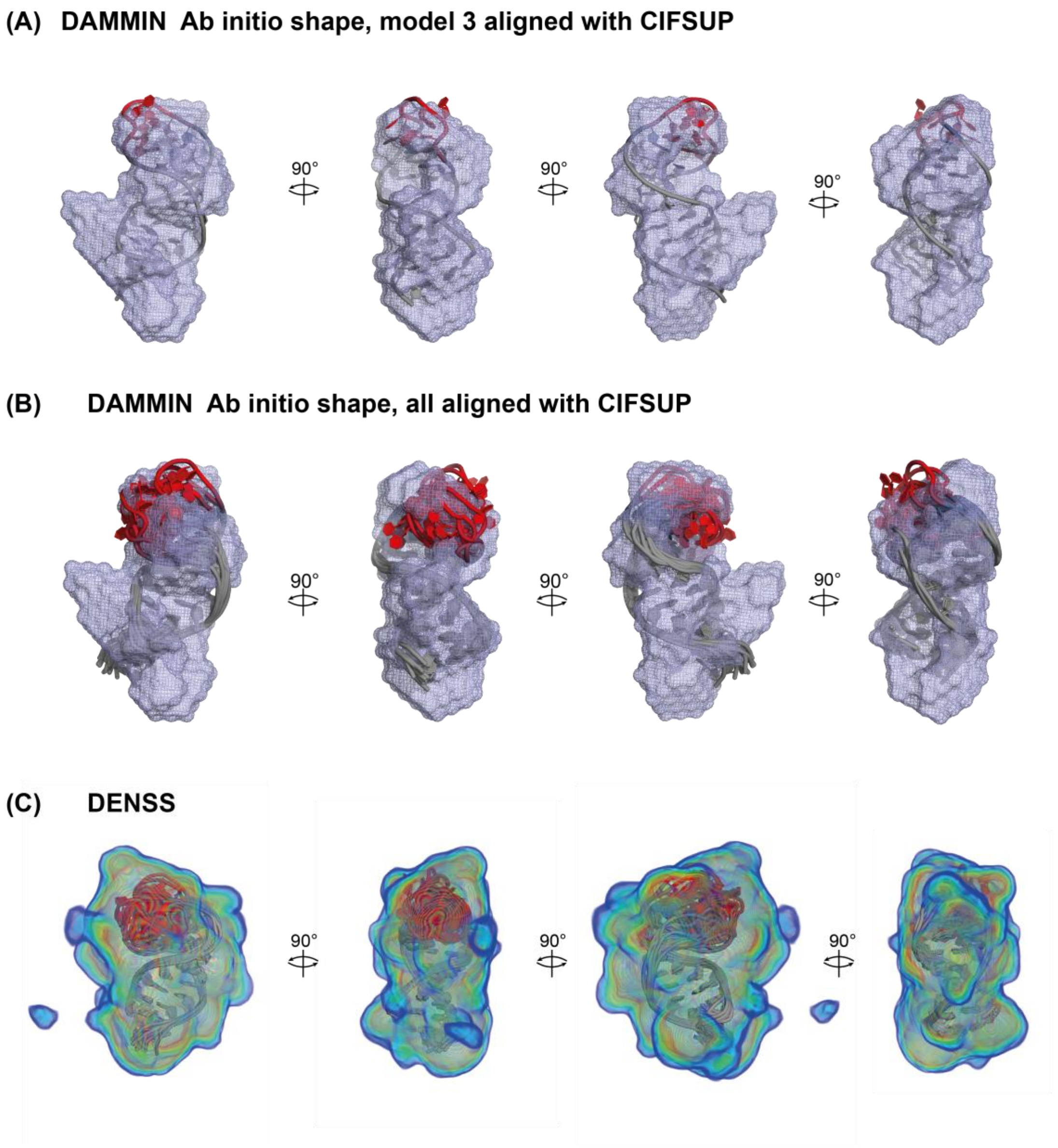

Supplementary Figure 16. Ab- initio models of s2m Delta_short from 1.5 mg/mL SAXS scattering data. (A) DAMMIN model (light blue) with model 3 coloured in hetNOE-derived heatmap colouring. Model building was performed with ATSAS 4.0.0.-2 software and aligned with CIFSUP from BioXTAS RAW 2.3.0 software. (B) DAMMIN modelling (light blue) with NMR ensemble (top 10) coloured in hetNOE derived heatmap coloring. Model building was performed with ATSAS 4.0.0.-2 software. Alignment of ensemble was performed with CIFSUP from BioXTAS RAW 2.3.0 software. (C) Density from solution scattering (DENSS) (heatmap coloring) with NMR ensemble (top 10) coloured in hetNOE derived heatmap coloring. Model building was performed with BioXTAS RAW 2.3.0 software.

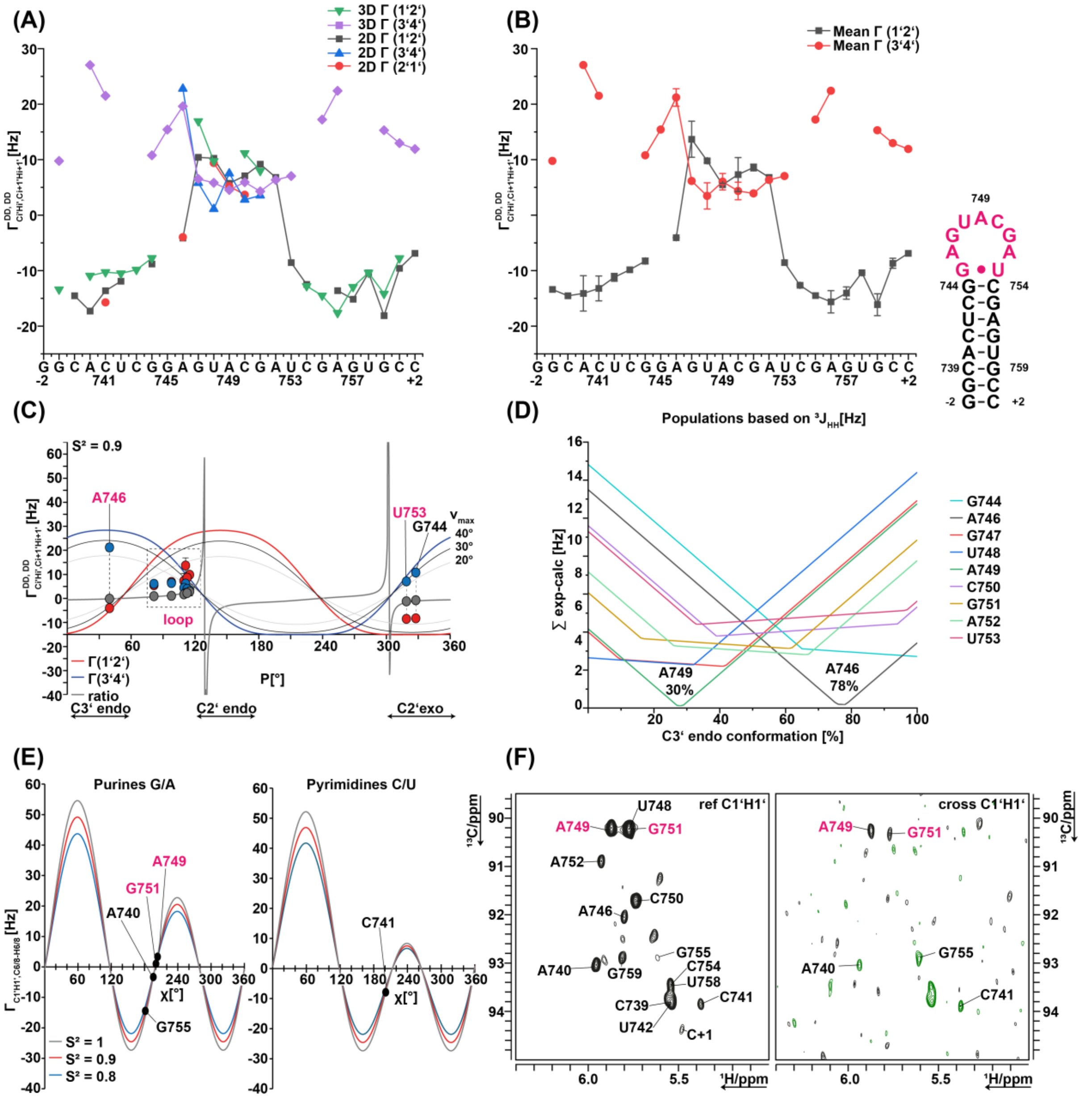

Supplementary Figure 17. CCR rates, ^{3}J coupling constants and χ angle determination of s2m Delta_short at 308 K. (A) Comparison of CCR rates from quantitative 2D Γ-HCCH and 3D *fw*-Γ-HCCH experiment. (B) Mean CCR rates from quantitative 2D Γ-HCCH and 3D *fw*-Γ-HCCH experiment. Errors are the standard deviation from the two experiments. (C) Determination of pseudorotation phase P from mean $\Gamma^{DD,DD}_{C1'H1', C2'H2'}$ and $\Gamma^{DD,DD}_{C3'H3', C4'H4'}$ values with varying ν_{max} of dynamic (G744, A746, U753) and loop nucleotides G747-A752. (D) C3′ endo population distribution of loop nucleotides from ^{3}J coupling constants. (E) χ angle determination with $\Gamma^{DD,DD}_{C1'H1'C6H6}$ and $\Gamma^{DD,DD}_{C1'H1'C8H8}$ CCR rates obtained from quantitative Γ-HCN experiment (F).

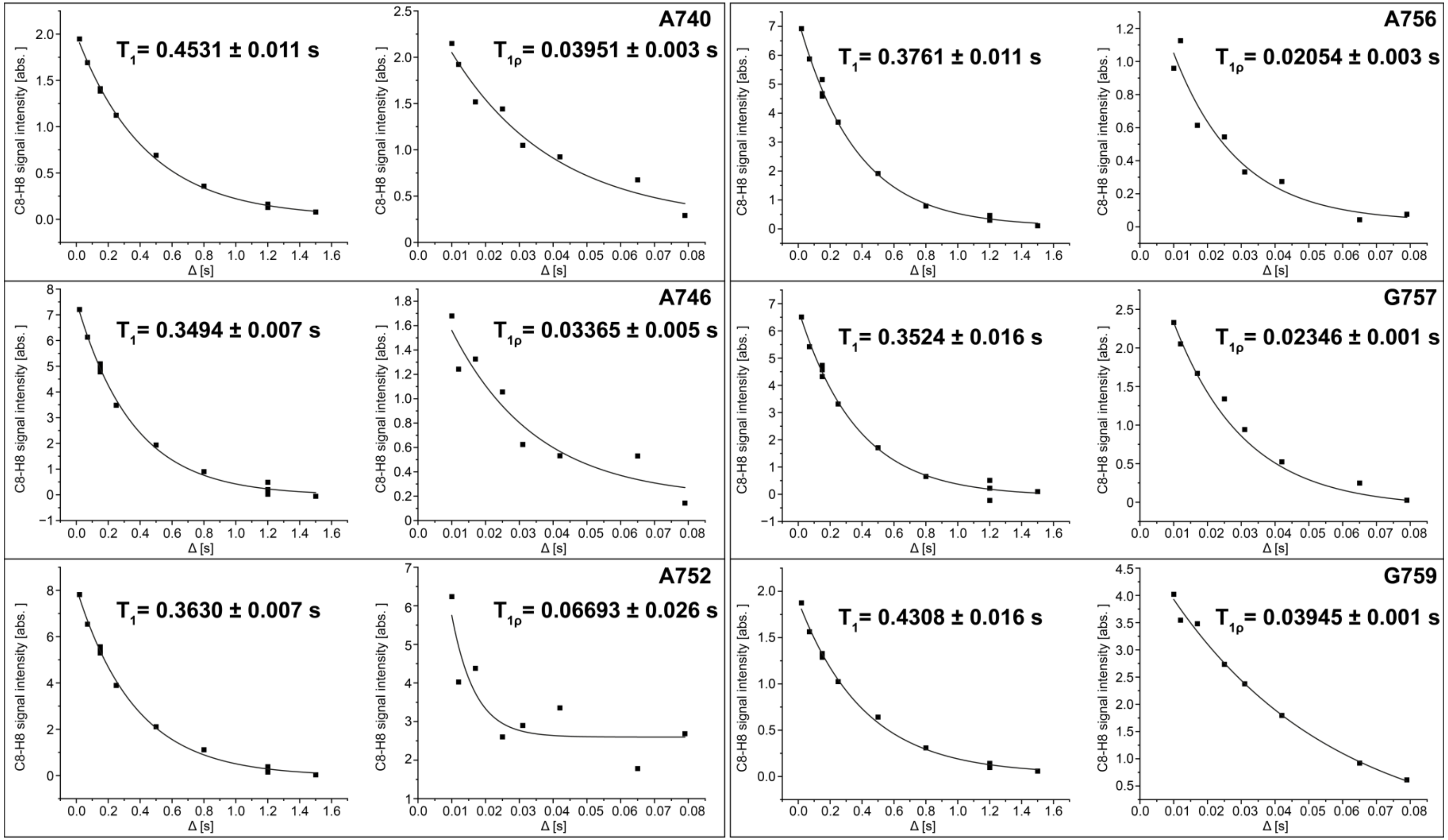

Supplementary Figure 18. Monoexponential fits of T_1 and $T_{1\rho}$ data for given nucleotides analysed with NMRFAM-SPARKY (1)**.**

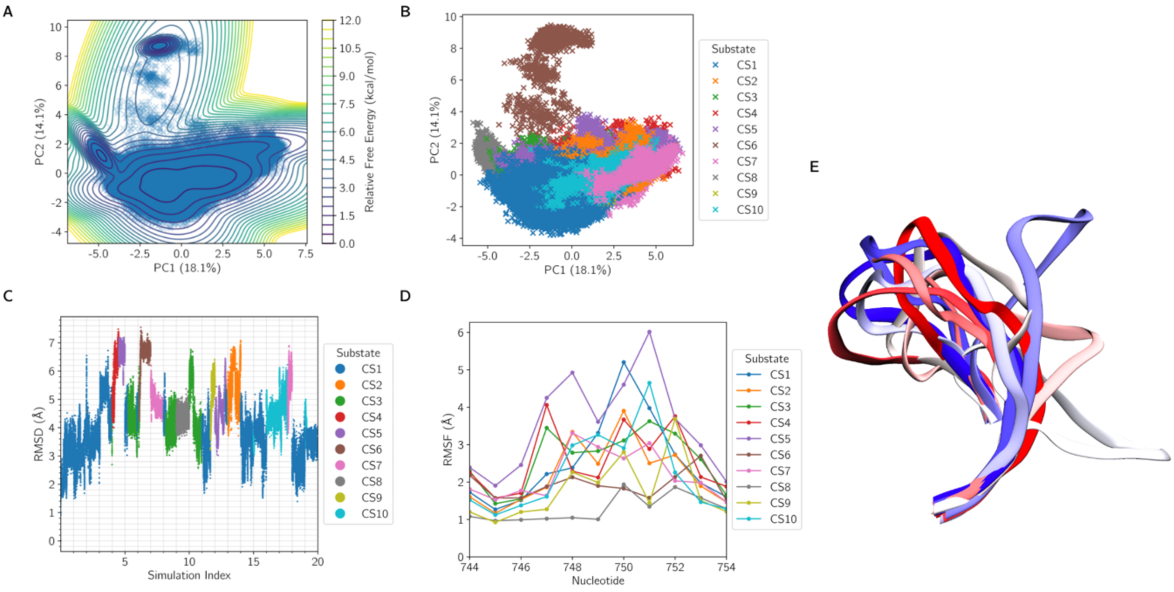

Supplementary Figure 19. Terminal loop PCA of 20 MD simulations. (A) Marginal estimate of the free energy landscape over the first two principal components. Due to the low proportion of variance captured in each component, it is difficult to resolve distinct conformational substates in just two components. (B) Clustering into 10 conformational substates with recurrence over distinct simulations given by (C) an RMSD plot colored by CS. (D) RMSF of nucleotides relative to centroid structure for each CS. (E) Centroid structures aligned to nucleotides 744 and 754 (CS1 = red to CS10 = blue).

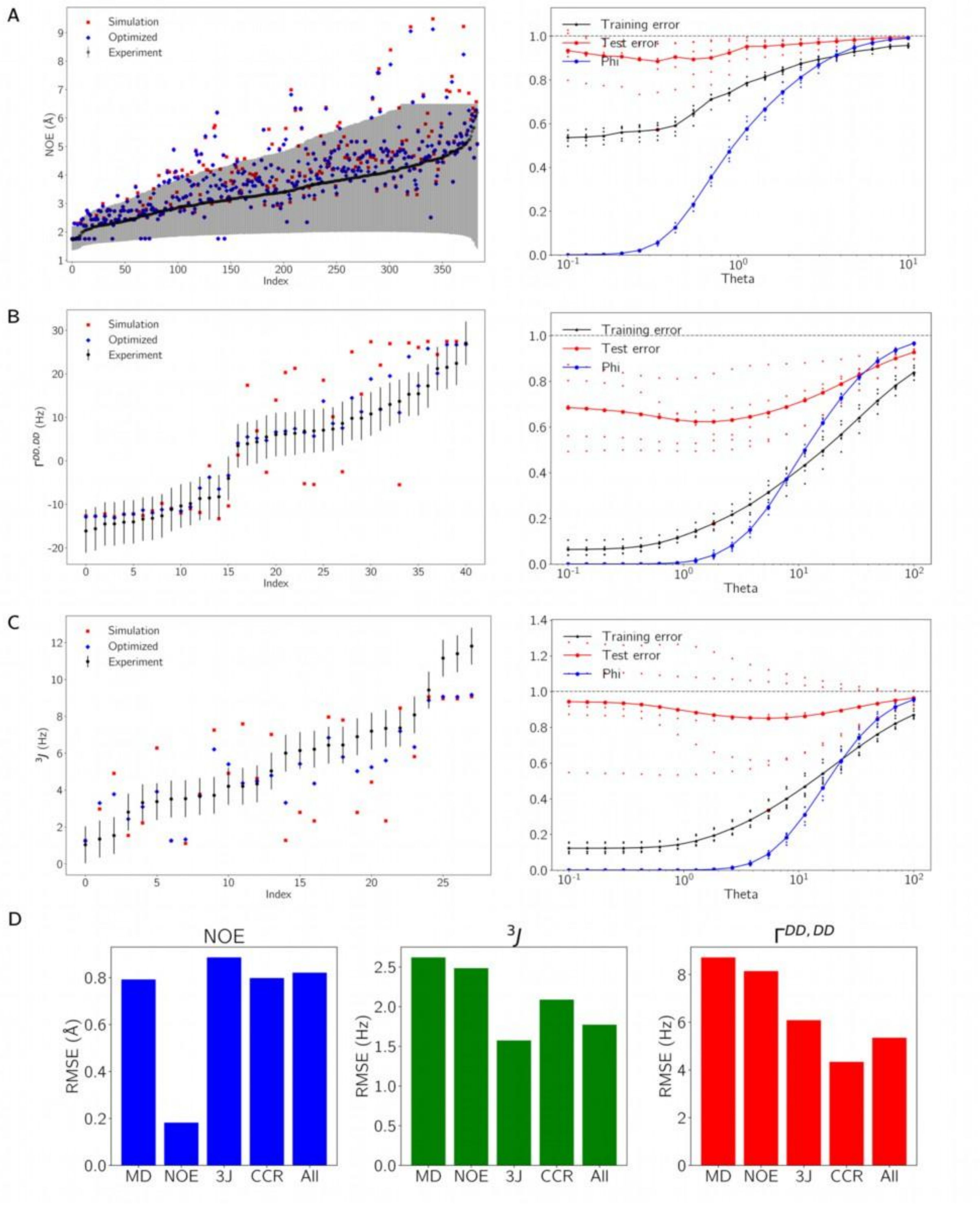

Supplementary Figure 20. Bayesian maximum entropy reweighting. Error with experimental means (left) and cross-validation (right) using (A) NOE, (B) CCR, or (C) ^3J means from experiment. (D) Root mean squared error of uniformly weighted MD simulations relative to reweighting based on NOE, CCR, or 3J for each of the three observables or all three simultaneously (All).

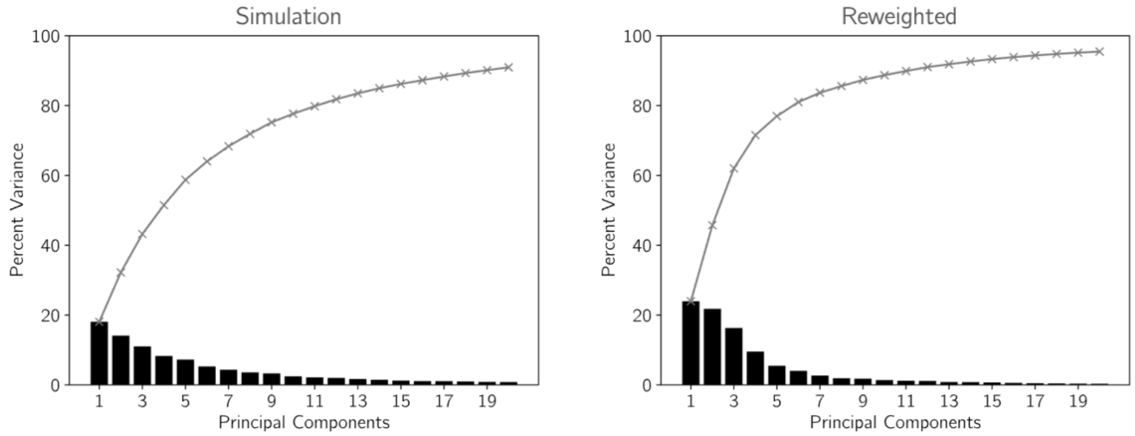

Supplementary Figure 21. Variance captured in terminal loop PCA. Distribution of variance across principal components for original 20 MD simulations (left) versus the CCR reweighted ensemble (right). Greater cumulative variance (gray line) is captured in fewer principal components by the reweighted ensemble, representing a smaller number of dynamical modes being identified as prominent in the data.

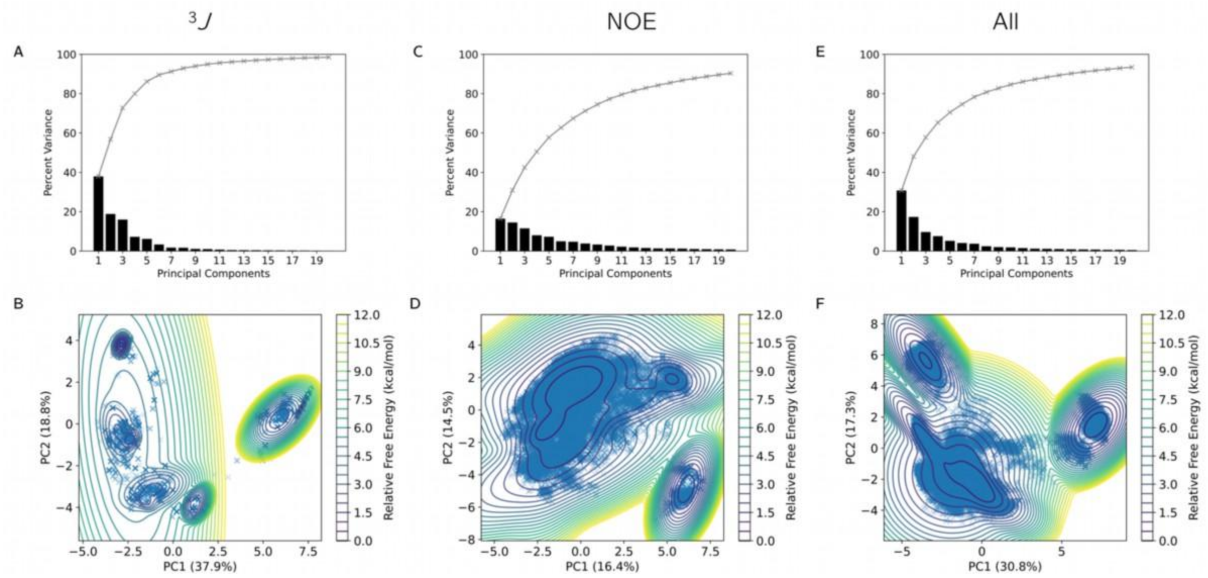

Supplementary Figure 22. Terminal loop PCA from ^3J and NOE reweighted ensembles. Principal component analysis of the ^{3}J coupling-based reweighted ensemble given by (A) the scree plot and (B) projection of structures onto the first two principal components. Likewise, principal component analysis of the NOE-based reweighted ensemble given by (C) the scree plot and (D) projection of structures onto the first two principal components and the scree plot (E) and projections (F) of the reweighted ensemble based on all three NMR observables simultaneously.

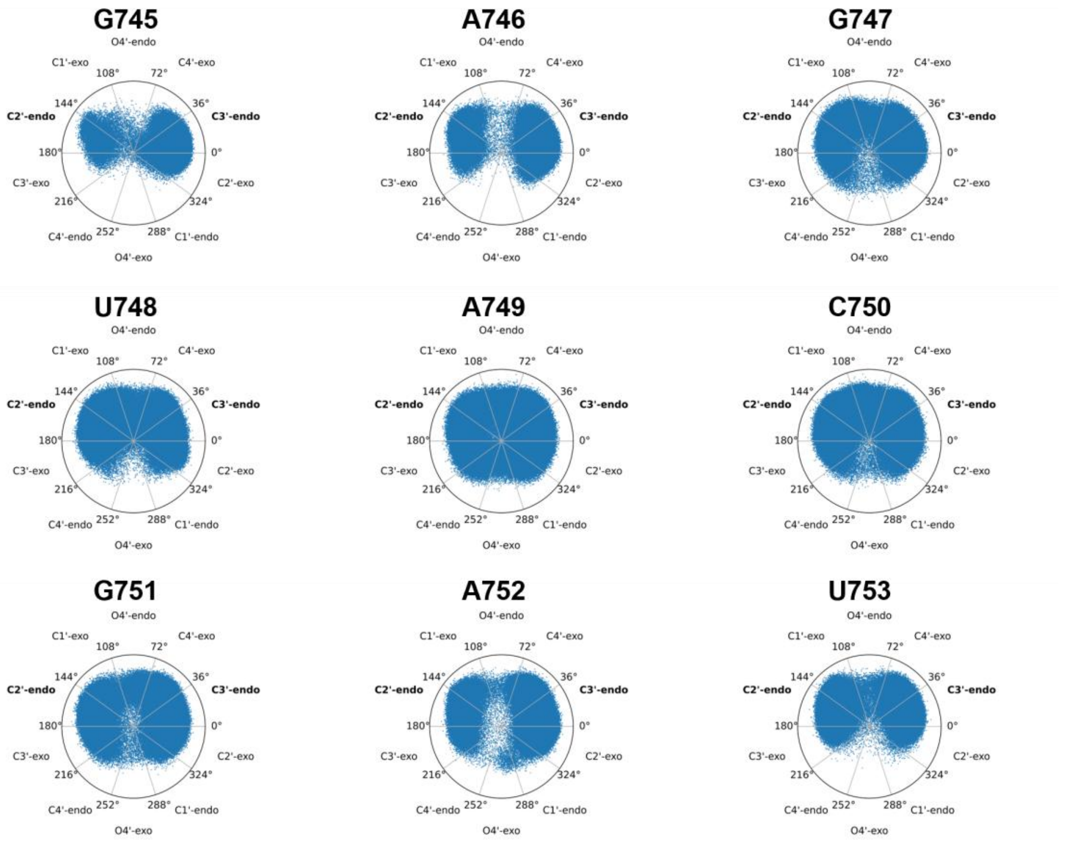

Supplementary Figure 23. Pseudorotation angles and amplitudes of the unweighted ensemble of loop nucleotide G745-U753.

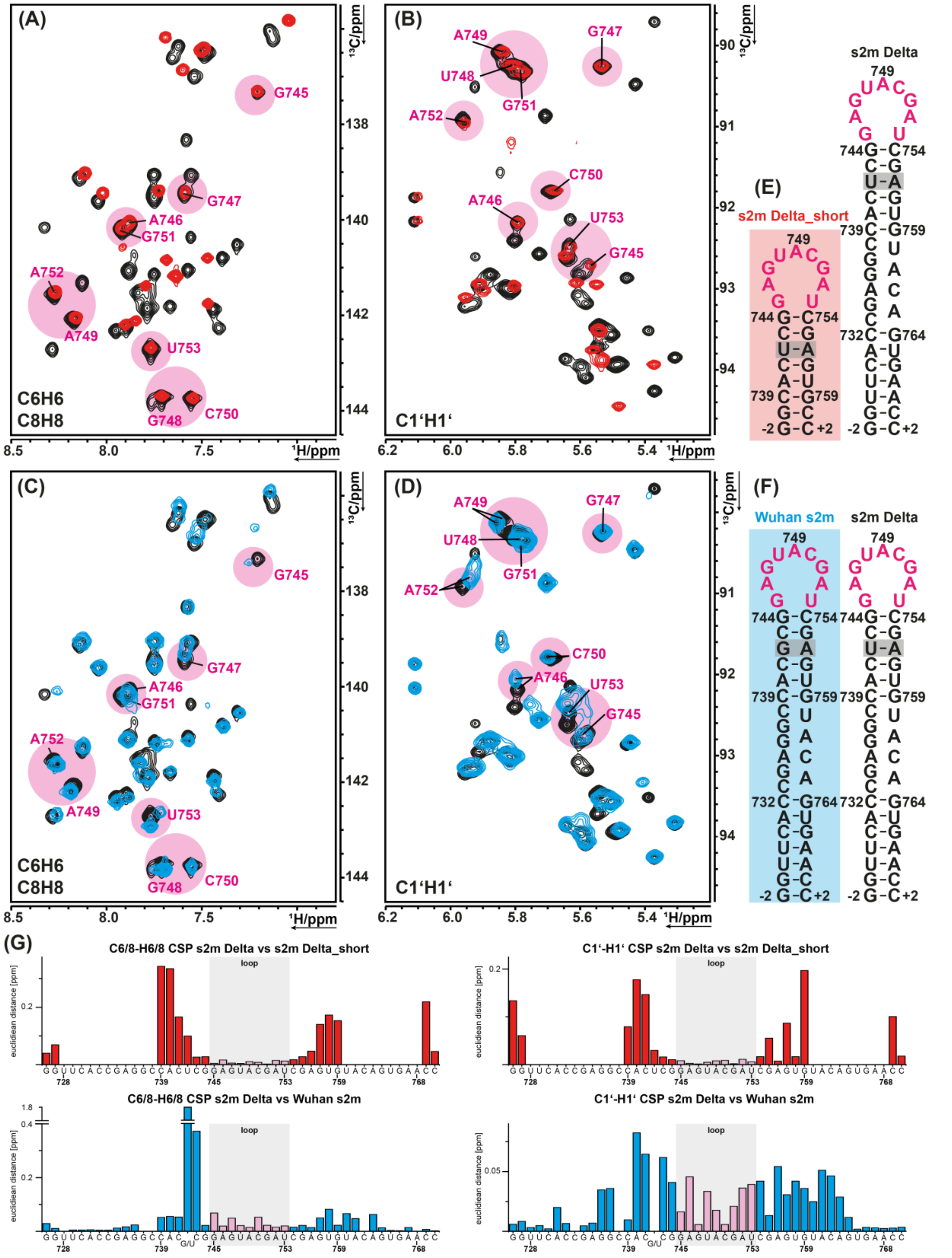

Supplementary Figure 24. Chemical shifts of loop signals in the aromatic and sugar region of Wuhan s2m, s2m Delta and s2m Delta short from SCoV-2. (A) Aromatic C6H6/C8H8 region of s2m Delta (black, sample#2) and s2m Delta_short (red, sample#3). (B) Sugar region C1'H1' of s2m Delta (black, sample#2) and s2m Delta_short (red, sample#3). (C) Aromatic C6H6/C8H8 region of s2m Delta (black, sample#2) and Wuhan s2m (light blue, sample#1). (D) Sugar region C1'H1' of s2m Delta (black, sample#2) and Wuhan s2m (light blue, sample#1). (E) Secondary structures of s2m Delta and s2m Delta_short. (F) Secondary structures of Wuhan s2m and s2m Delta. Assigned loop nucleotides are noted in magenta also highlighted with regions marked in light red. (G) Euclidian distance analysis of aromatic C6/8-H6/8 and aromatic C1'H1' chemical shifts using the formula as described before in ((2)).

SUPPLEMENTARY TABLES

Supplementary Table 1. S2m RNA sequences. Nucleotides marked in red are additional GC base pairs not part of the RNA genome. Bold nucleotides are mutations from SCoV-1 to SCoV-2. Nucleotides marked in green are mutations from SCoV-2 Wuhan s2m to SCoV-2 Delta.

Origin	RNA sequence	Position
**SCoV-1 s2m**	**GG**UUCA**U**CGAGGCCACGCGGAGUACGAUCGAG**G**GUACAGUGAA**CC**	29,728-29,768
**SCoV-2 Wuhan s2m**	**GG**UUCA**C**CGAGGCCAC**G**CGGAGUACGAUCGAG**U**GUACAGUGAA**CC**	29,728-29,768
**SCoV-2 s2m Delta**	**GG**UUCA**C**CGAGGCCAC**U**CGGAGUACGAUCGAG**U**GUACAGUGAA**CC**	29,728-29,768
**SCoV-2 s2m Delta_short**	**GG**CACU**C**GGAGUACGA**U**CGAGUG**CC**	29,739-29,759

Supplementary Table 2. RNA samples for NMR measurements. All RNA samples for NMR measurements were rebuffered in 25 mM K_2HPO_4/KH_2PO_4, 50 mM KCl, pH 6.2. Sample#4 had to be diluted to sample#4.5 after 4D-HMQC-NOESY-HMQC measurement because of degradation.

Sample number	RNA	Labelling	Solvent for locking	concentration
**1**	SCoV-2 Wuhan s2m	^{13}C, ^{15}N	5% D_2O, 95% H_2O	600 µM
**2**	SCoV-2 s2m Delta	^{13}C, ^{15}N	5% D_2O, 95% H_2O	370 µM
**3**	SCoV-2 s2m Delta_short	^{13}C, ^{15}N	5% D_2O, 95% H_2O	250 µM
**4**	SCoV-2 s2m Delta_short	^{13}C, ^{15}N	100% D_2O	1.7 mM
**4.5**	SCoV-2 s2m Delta_short	^{13}C, ^{15}N	100% D_2O	155 µM
**5**	SCoV-2 s2m Delta_short	^{13}C, ^{15}N (AU)	100% D_2O	150 µM
**6**	SCoV-2 s2m Delta_short	^{13}C, ^{15}N (C)	100% D_2O	200 µM
**7**	SCoV-2 s2m Delta_short	unlabelled	100% D_2O	700 µM
**8**	SCoV-2 3'-UTR	^{15}N	5% D_2O, 95% H_2O	180 µM
**9**	SCoV-2 HVR	^{13}C, ^{15}N (GU)	5% D_2O, 95% H_2O	841 µM
**10**	SCoV-2 s2m Delta_short	^{13}C, ^{15}N	100% D_2O	600 µM

Supplementary Table 3. RNA samples for Nsp15 endoribonuclease assay. All RNA samples for Nsp15 endoribonuclease assay were rebuffered in 25 mM NaPi pH 7.5; 300 mM NaCl; 5 mM $MgCl_2$; 5 mM DTT; 2 mM $MnCl_2$. Nucleotides marked in red are additional GC base pairs not part of the RNA genome.

Number	RNA	Sequence
**1**	SCoV-1 s2m	**GG**UUCAUCGAGGCCACGCGGAGUACGAUCGAGGGUACAGUGAA**CC**
**2**	SCoV-2 Wuhan s2m	**GG**UUCACCGAGGCCACGCGGAGUACGAUCGAGUGUACAGUGAA**CC**
**3**	SCoV-2 s2m Delta	**GG**UUCACCGAGGCCACUCGGAGUACGAUCGAGUGUACAGUGAA**CC**
**4**	SCoV-2 s2m Delta_short	**GG**CACUCGGAGUACGAUCGAGUG**CC**

Supplementary Table 4. NMR experiments and parameters measured at 283 K.

Experiment	Parameters
**^{1}H, ^{1}H NOESY (jump return)** (3, 4)	600 MHz, ns: 144, sw(f2): 21 ppm, sw(f1): 11.9 ppm, TD(f2): 2048, TD(f1): 448, aq (f2): 82 ms, o1p (^{1}H): 4.7 ppm, d1: 1s, frequency jump to 8.75 ppm, mixing time: 150 ms, ca. 23 h, sample#3
**^{1}H, ^{15}N BEST-TROSY** (5–8)	600 MHz, ns: 16, sw(f2): 21 ppm, sw(f1): 25 ppm, TD(f2): 2018, TD(f1): 512, aq (f2): 80 ms, o1p (^{1}H): 4.7 ppm, o2p (^{13}C): 101 ppm, o3p(^{15}N): 153 ppm, d1: 0.3 s, ca. 1 h, sample#3
**HNN-COSY** (9, 10)	600 MHz, ns: 32, sw(f3): 20 ppm, sw(f2): 30 ppm, sw(f1): 100 ppm, TD(f3): 2500, TD(f2): 1, TD(f1): 384, aq (f3): 105 ms, o1p (^{1}H): 4.7 ppm, o2p: (^{13}C): 105 ppm, o3p(^{15}N): 183 ppm, d1: 0.3 s, 1 h 43 min, sample#3
**HNCO** (5–8)	800 MHz, ns: 256, sw(f3): 21 ppm, sw(f2): 25 ppm, sw(f1): 31 ppm, TD(f3): 1582, TD(f2): 1, TD(f1): 256, aq (f3): 50 ms, o1p (^{1}H): 4.7 ppm, o2p (^{13}C): 157 ppm, o3p(^{15}N): 153 ppm, d1: 0.3 s, 8 h 13 min, sample#3
**^{1}H, ^{13}C HSQC** (11, 12)	*aromatic region*:<br>700 MHz, ns: 4, sw(f2): 8.3 ppm, sw(f1): 24 ppm, TD(f2): 1024, TD(f1): 384, aq (f2): 87 ms, o1p (^{1}H): 4.7 ppm, o2p (^{13}C): 143 ppm, d1: 1 s, INEPT transfer time: 2.5 ms, 30 min, sample#3<br>*C1′ region:*<br>700 MHz, ns: 4, sw(f2): 8.3 ppm, sw(f1): 12 ppm, TD(f2): 1024, TD(f1): 384, aq (f2): 87 ms, o1p (^{1}H): 4.7 ppm, o2p (^{13}C): 90 ppm, d1: 1 s, INEPT transfer time: 2.94 ms, 30 min, sample#3

Supplementary Table 5. NMR experiments and parameters measured at 298 K.

Experiment	Parameters
**^{1}H, ^{1}H NOESY (jump return)**<br>(3, 4)	800 MHz, ns: 256, sw(f2): 21 ppm, sw(f1): 11.9 ppm, TD(f2): 2048, TD(f3): 384, aq (F2): 60 ms, o1p (^{1}H): 4.7 ppm, d1: 1s, frequency jump to 8.75 ppm, mixing time: 150 ms, 34 h, sample#3
**^{1}H, ^{15}N BEST-TROSY**<br>(5–8)	600 MHz, ns: 16, sw(f2): 21 ppm, sw(f1): 25 ppm, TD(f2): 2018, TD(f1): 512, aq (f2): 80 ms, o1p (^{1}H): 4.7 ppm, o2p (^{13}C): 101 ppm, o3p (^{15}N): 153 ppm, d1: 0.3 s, ca. 1 h, sample#3<br>sample#8<br>sample#9<br>sample#1<br>sample#2
**^{1}H, ^{15}N- HSQC (amino)**<br>(13)	600 MHz, ns: 16, sw(f2): 10 ppm, sw(f1): 30 ppm, TD(f2): 1024, TD(f1): 256, aq (f2): 85 ms, o1p (^{1}H): 4.7 ppm, o2p (^{13}C): 101 ppm, o3p (^{15}N): 86.5 ppm, d1: 0.8 s, ca. 1 h, sample#3
**^{1}H, ^{15}N CPMG NOESY**<br>(14, 15)	600 MHz, ns: 144, sw(f2): 21 ppm, sw(f1): 103 ppm, TD(f2): 2048, TD(f1): 256, aq (f2): 81 ms, o1p (^{1}H): 4.7 ppm, o2p (^{13}C): 101 ppm, o3p (^{15}N): 116 ppm, d1: 1 s, mixing time: 150 ms, ca. 13 h, sample#3
**HNCO**<br>(5–8)	800 MHz, ns: 256, sw(f3): 21 ppm, sw(f2): 25 ppm, sw(f1): 31 ppm, TD(f3): 1582, TD(f2): 1, TD(f1): 256, aq (f3): 50 ms, o1p (^{1}H): 4.7 ppm, o2p (^{13}C): 157 ppm, o3p(^{15}N): 153 ppm, d1: 0.3 s, 8 h 13 min, sample#3
**^{1}H,^{13}C HSQC**<br>(11, 12)	*aromatic region*:<br>600 MHz, ns: 4, sw(f2): 8.3 ppm, sw(f1): 24 ppm, TD(f2): 1024, TD(f1): 384, aq (f2): 102 ms, o1p (^{1}H): 4.7 ppm, o2p (^{13}C): 143 ppm, d1: 1 s, INEPT transfer time: 2.5 ms, 30 min, sample#3<br>*C1′ region:*<br>600 MHz, ns: 4, sw(f2): 8.3 ppm, sw(f1): 12 ppm, TD(f2): 1024, TD(f1): 384, aq (f2): 102 ms, o1p (^{1}H): 4.7 ppm, o2p (^{13}C): 90 ppm, d1: 1 s, INEPT transfer time: 2.94 ms, 30 min, sample#3
**^{1}H, ^{15}N- HSQC (2J)**<br>(13)	600 MHz, ns: 64, sw(f2): 10 ppm, sw(f1): 85 ppm, TD(f2): 1024, TD(f1): 256, aq (f2): 85 ms, o1p (^{1}H): 4.7 ppm, o2p (^{13}C): 101 ppm, o3p (^{15}N): 201 ppm, d1: 1 s, ca. 5 h 12 min, sample#3
**LR-HSQC**<br>(16)	800 MHz, ns: 256, sw(f2): 21 ppm, sw(f1): 10 ppm, TD(f2): 2300, TD(f1): 144, aq (f2): 70 ms, o1p (^{1}H): 4.7 ppm, o2p (^{13}C): 117 ppm, o3p (^{15}N): 150 ppm, d1: 1 s, ca. 12 h, sample#3

Supplementary Table 6. NMR experiments and parameters measured at 308 K.

Experiment	Parameters
**^{1}H, ^{1}H NOESY for structure determination** (17)	700 MHz, ns: 112, sw(f2): 14 ppm, sw(f1): 8 ppm, TD(f2): 2048, TD(f1): 512, aq (f2): 104 ms, o1p (^{1}H): 4.7 ppm, d1: 2 s, frequency jump to 8.75 ppm, mixing time: 150 ms, 100 ms, 50 ms ca. 1 d 12 h, sample#7
**^{1}H, ^{15}N BEST-TROSY** (5–8)	800 MHz, ns: 16, sw(f2): 21 ppm, sw(f1): 25 ppm, TD(f2): 2018, TD(f1): 280, aq (f2): 60 ms, o1p (^{1}H): 4.7 ppm, o2p (^{13}C): 101 ppm, o3p (^{15}N): 153 ppm, d1: 0.3 s, ca. 31 min, sample#3
**HNCO** (5–8)	800 MHz, ns: 256, sw(f3): 21 ppm, sw(f2): 25 ppm, sw(f1): 31 ppm, TD(f3): 1582, TD(f2): 1, TD(f1): 256, aq (f3): 50 ms, o1p (^{1}H): 4.7 ppm, o2p (^{13}C): 157 ppm, o3p(^{15}N): 153 ppm, d1: 0.3 s, 8 h 13 min, sample#3
**LR-HSQC** (16)	800 MHz, ns: 256, sw(f2): 21 ppm, sw(f1): 10 ppm, TD(f2): 2300, TD(f1): 144, aq (f2): 70 ms, o1p (^{1}H): 4.7 ppm, o2p (^{13}C): 117 ppm, o3p (^{15}N): 150 ppm, d1: 1 s, ca. 12 h, sample#3
**^{1}H,^{13}C HSQC** (11, 12)	*aromatic region*:<br>At 600 MHz spectrometer:<br>ns: 4, sw(f2): 8.3 ppm, sw(f1): 24 ppm, TD(f2): 1024, TD(f1): 384, aq (f2): 102 ms, o1p (^{1}H): 4.7 ppm, o2p (^{13}C): 143 ppm, d1: 1 s, INEPT transfer time: 2.5 ms, 30 min, sample#3<br><br>At 900 MHz spectrometer:<br>ns: 4, sw(f2): 8.3 ppm, sw(f1): 24 ppm, TD(f2): 1024, TD(f1): 384, aq (f2): 70 ms, o1p (^{1}H): 4.7 ppm, o2p (^{13}C): 143 ppm, d1: 1 s, INEPT transfer time: 2.5 ms, 30 min, sample#4<br><br>At 700 MHz spectrometer:<br>ns: 8, sw(f2): 8.3 ppm, sw(f1): 24 ppm, TD(f2): 1024, TD(f1): 384, aq (f2): 87 ms, o1p (^{1}H): 4.7 ppm, o2p (^{13}C): 143 ppm, d1: 1 s, INEPT transfer time: 2.5 ms, 1 h, sample#4.5<br><br>^{13}C, ^{15}N (AU) labelled sample:<br>800 MHz, ns: 4, sw(f2): 8.3 ppm, sw(f1): 24 ppm, TD(f2): 1024, TD(f1): 384, aq (f2): 76 ms, o1p (^{1}H): 4.7 ppm, o2p (^{13}C): 143 ppm, d1: 1 s, INEPT transfer time: 2.5 ms, 30 min, sample#5<br><br>^{13}C, ^{15}N (C) labelled sample:<br>800 MHz, ns: 4, sw(f2): 8.3 ppm, sw(f1): 24 ppm, TD(f2): 1024, TD(f1): 384, aq (f2): 76 ms, o1p (^{1}H): 4.7 ppm, o2p (^{13}C): 143 ppm, d1: 1 s, INEPT transfer time: 2.5 ms, 30 min, sample#6<br><br>*C1' region:*<br>At 600 MHz spectrometer:<br>ns: 8, sw(f2): 8.3 ppm, sw(f1): 12 ppm, TD(f2): 1024, TD(f1): 384, aq (f2): 102 ms, o1p (^{1}H): 4.7 ppm, o2p (^{13}C): 90 ppm, d1: 1 s, INEPT transfer time: 2.94 ms, 1 h, sample#3<br><br>At 900 MHz spectrometer:<br>ns: 2, sw(f2): 8.3 ppm, sw(f1): 12 ppm, TD(f2): 1024, TD(f1): 384, aq (f2): 70 ms, o1p (^{1}H): 4.7 ppm, o2p (^{13}C): 90 ppm, d1: 1 s, INEPT transfer time: 2.94 ms, 14 min, sample#4<br><br>At 800 MHz spectrometer:

ns: 4, sw(f2): 8.3 ppm, sw(f1): 12 ppm, TD(f2): 1024, TD(f1): 384, aq (f2): 76 ms, o1p (^{1}H): 4.7 ppm, o2p (^{13}C): 90 ppm, d1: 1 s, INEPT transfer time: 2.94 ms, 30 min, sample#4.5

^{13}C, ^{15}N (AU) labelled sample:
800 MHz, ns: 4, sw(f2): 8.3 ppm, sw(f1): 12 ppm, TD(f2): 1024, TD(f1): 384, aq (f2): 76 ms, o1p (^{1}H): 4.7 ppm, o2p (^{13}C): 90 ppm, d1: 1 s, INEPT transfer time: 2.94 ms, 30 min, sample#5

^{13}C, ^{15}N (C) labelled sample:
800 MHz, ns: 4, sw(f2): 8.3 ppm, sw(f1): 12 ppm, TD(f2): 1024, TD(f1): 384, aq (f2): 76 ms, o1p (^{1}H): 4.7 ppm, o2p (^{13}C): 90 ppm, d1: 1 s, INEPT transfer time: 2.94 ms, 30 min, sample#6

CT HSQC ribose C1′ to C5′:
700 MHz, ns: 8, sw(f2): 8.3 ppm, sw(f1): 38 ppm, TD(f2): 1024, TD(f1): 256, aq (f2): 88 ms, o1p (^{1}H): 4.7 ppm, o2p (^{13}C): 77 ppm, d1: 1 s, constant time delay: 12.5 ms, 40 min, sample#4.5

^{13}C, ^{15}N (AU) labelled sample:
800 MHz, ns: 4, sw(f2): 8.3 ppm, sw(f1): 38 ppm, TD(f2): 1024, TD(f1): 256, aq (f2): 76 ms, o1p (^{1}H): 4.7 ppm, o2p (^{13}C): 77 ppm, d1: 1 s, constant time delay: 12.5 ms, 20 min, sample#5

^{13}C, ^{15}N (C) labelled sample:
800 MHz, ns: 8, sw(f2): 8.3 ppm, sw(f1): 38 ppm, TD(f2): 1024, TD(f1): 256, aq (f2): 88 ms, o1p (^{1}H): 4.7 ppm, o2p (^{13}C): 77 ppm, d1: 1 s, constant time delay: 12.5 ms, 40 min, sample#4.5

C5′-H5′′ HSQC:
At 800 MHz spectrometer:
ns: 4, sw(f2): 8.3 ppm, sw(f1): 12 ppm, TD(f2): 1024, TD(f1): 384, aq (f2): 76 ms, o1p (^{1}H): 4.7 ppm, o2p (^{13}C): 62 ppm, d1: 1 s, INEPT transfer time: 3.32 ms, 30 min, sample#4

At 600 MHz spectrometer:
ns: 8, sw(f2): 8.3 ppm, sw(f1): 12 ppm, TD(f2): 1024, TD(f1): 384, aq (f2): 76 ms, o1p (^{1}H): 4.7 ppm, o2p (^{13}C): 62 ppm, d1: 1 s, INEPT transfer time: 3.32 ms, 1 h, sample#4.5

^{13}C, ^{15}N (AU) labelled sample:
600 MHz, ns: 4, sw(f2): 8.3 ppm, sw(f1): 12 ppm, TD(f2): 1024, TD(f1): 384, aq (f2): 76 ms, o1p (^{1}H): 4.7 ppm, o2p (^{13}C): 62 ppm, d1: 1 s, INEPT transfer time: 3.32 ms, 30 min, sample#5

^{13}C, ^{15}N (C) labelled sample:
800 MHz, ns: 4, sw(f2): 8.3 ppm, sw(f1): 12 ppm, TD(f2): 1024, TD(f1): 384, aq (f2): 76 ms, o1p (^{1}H): 4.7 ppm, o2p (^{13}C): 62 ppm, d1: 1 s, INEPT transfer time: 3.32 ms, 30 min, sample#6

C5-H5 HSQC:
600 MHz, ns: 8, sw(f2): 8.3 ppm, sw(f1): 16 ppm, TD(f2): 1024, TD(f1): 384, aq (f2): 102 ms, o1p (^{1}H): 4.7 ppm, o2p (^{13}C): 100 ppm, d1: 1 s, INEPT transfer time: 3.32 ms, 1 h, sample#4.5

**3D HCN**<br>(18)	*^1H detected:*<br><br>800 MHz, ns: 24, sw(f3): 9 ppm, sw(f2): 22 ppm, sw(f1): 33 ppm, TD(f3): 1024, TD(f2): 128, TD(f1): 168, aq (f3): 70 ms, o1p (^1H): 4.7 ppm, o2p (^{13}C): 115 ppm, o3p(^{15}N): 158 ppm, d1: 1 s, INEPT transfer time: 2.8 ms (HC) and 34 ms(CN), recorded with 30% NUS, 2 d 3 h, sample#3
**HCCNH**<br>(19, 20)	600 MHz, ns: 200, sw(f3): 21 ppm, sw(f2): 10 ppm, sw(f1): 30 ppm, TD(f3): 2254, TD(f2): 128, TD(f1): 1, aq(f3): 90 ms, o1p(^1H): 4.7 ppm, o2p(^{13}C): 137 ppm, o3p(^{15}N): 154 ppm, d1: 1.8 s, C6/8-N1/9 transfer time: 24 ms, TOCSY mixing time (H6/8)C6/8(CC)NH: 28 ms,<br>15 h, sample#3
**TROSY-HCCH-COSY**<br>(21)	600 MHz, ns: 16, sw(f3): 8.7 ppm, sw(f2): 22 ppm, sw(f1): 58 ppm TD(f3): 1024, TD(f2): 64, TD(f1):128, aq (f3): 97 ms, o1p (^1H): 4.7 ppm, o2p (^{13}C):143 ppm, d1: 1s, 1 d 20 h, sample#4
**3D NOESY**<br>(22)	800 MHz, ns: 16, sw(f3): 8.7 ppm, sw(f2): 21 ppm, sw(f1): 6.3 ppm TD(f3): 1024, TD(f2): 88, TD(f1):168, aq (f3): 73 ms, o1p (^1H): 4.7 ppm, o2p (^{13}C):143 ppm, d1: 1s, mixing time: 300 ms, 1 d 20 h, sample#3
**4D HMQC-NOESY-HMQC**<br>(23)	900 MHz, ns: 8, sw(f4): 9.1 ppm, sw(f3): 19 ppm, sw(f2): 22 ppm, sw(f1): 2.8 ppm TD(f4): 1146, TD(f3): 48, TD(f2): 48, TD(f1): 128, aq(f4): 69 ms, o1p(^1H): 4.7 ppm, o2p(^{13}C, f3): 143 ppm, o2p(^{13}C, f2): 137 ppm, o3p(^{15}N): 157 ppm, d1: 1.5 s, INEPT transfer times: 2.5 ms (aromatics) and 3.1 ms (H1'C1'), NOESY mixing time: 150 ms, recorded with 14% NUS, 6 d 19 h, sample#4
**3D HCC(H)-TOCSY**<br>(24)	800 MHz, ns: 8, sw(f3): 8.5 ppm, sw(f2): 9.5 ppm, sw(f1): 35.5 ppm TD(f3): 1024, TD(f2): 48, TD(f1): 160 aq(f3): 74 ms, o1p(^1H): 4.7 ppm, o2p(^{13}C): 76.5 ppm, o3p(^{15}N): 153 ppm, d1: 0.97 s, INEPT transfer times: 3.2 ms, TOCSY mixing time: 16 ms, 20 h, sample#4
**3D *fw*-HCCH-TOCSY**<br>(24–27)	600 MHz, ns: 8, sw(f3): 15 ppm, sw(f2): 35 ppm, sw(f1): 3.4 ppm TD(f3): 1440, TD(f2): 80, TD(f1): 304 aq(f3): 80 ms, o1p(^1H): 4.7 ppm, o2p(^{13}C): 77 ppm, o3p(^{15}N): 155 ppm, d1: 1.05 s, INEPT transfer times: 3.4 ms, TOCSY mixing time: 12 ms, constant time delay: 8.3 ms, 2 d 19 h, sample#4<br><br>^{13}C, ^{15}N (AU) labelled sample:<br>800 MHz, ns: 16, sw(f3): 15 ppm, sw(f2): 35 ppm, sw(f1): 3.5 ppm TD(f3): 2048, TD(f2): 72, TD(f1): 128 aq(f3): 85 ms, o1p(^1H): 4.7 ppm, o2p(^{13}C): 77 ppm, o3p(^{15}N): 155 ppm, d1: 1.5 s, INEPT transfer times: 3.4 ms, TOCSY mixing time: 12 ms, constant time delay: 8.3 ms, 2 d 21 h,<br>sample#5<br><br>^{13}C, ^{15}N (C) labelled sample:<br>600 MHz, ns: 16, sw(f3): 15 ppm, sw(f2): 35 ppm, sw(f1): 3.4 ppm TD(f3): 1440, TD(f2): 72, TD(f1): 128 aq(f3): 80 ms, o1p(^1H): 4.7 ppm, o2p(^{13}C): 77 ppm, o3p(^{15}N): 155 ppm, d1: 1.35 s, INEPT transfer times: 3.4 ms, TOCSY mixing time: 12 ms, constant time delay: 8.3 ms, 2 d 14 h,<br>sample#6
**$^{13}C^{15}N$-HSQC Amino**<br>(28)	800 MHz, ns: 32, sw(f2): 50 ppm, sw(f1): 43 ppm, TD(f2): 1024, TD(f1): 76, aq (f2): 50 ms, o1p (^1H): 4.7 ppm, o2p (^{13}C):160 ppm, o3p(^{15}N): 86.5 ppm, d1: 2.5 s, 3 h 30 min, sample#4
**$^{13}C^{15}N$-HDQC Amino**<br>(28)	800 MHz, ns: 216, sw(f2): 50 ppm, sw(f1): 2.8 ppm, TD(f2): 2014, TD(f1): 80, aq (f2): 100 ms, o1p (^1H): 7.2 ppm, o2p (^{13}C):155 ppm, o3p(^{15}N): 90 ppm, d1: 2.5 s, 1 d 1 h, sample#4
**$^{13}C^{15}N$-HSQC**<br>(29)	800 MHz, ns: 512, sw(f2): 50 ppm, sw(f1): 35 ppm, TD(f2): 1024, TD(f1): 104, aq (f2): 50 ms, o1p (^{13}C):160 ppm, o2p(^{15}N): 157 ppm, d1: 1 s, 17 h,<br>Delay during INEPT: 0 ms, 15 ms, 34.7 ms, sample#10
**^1H, ^{13}C Heteronuclear NOE**<br>(30, 31)	*aromatic region:*<br><br>800 MHz, ns: 48, sw(f3): 9 ppm, sw(f2): 10 ppm, TD(f3): 1024, TD(f2): 310, aq(f3): 71 ms, o1p(^1H): 4.7 ppm, o2p(^{13}C): 137 ppm, o3p(^{15}N): 153 ppm, d1: 5 s, presaturation delay: 3 s, INEPT transfer time: 2.7 ms, 2 d 19 h, sample#4.5

	600 MHz, ns: 32, sw(f3): 9 ppm, sw(f2): 10 ppm, TD(f3): 1024, TD(f2): 256, aq(f3): 87ms, o1p(^1H): 4.7 ppm, o2p(^{13}C): 137 ppm, o3p(^{15}N): 153 ppm, d1: 5 s, presaturation delay: 3 s, INEPT transfer time: 2.7 ms, 1 d 13 h, sample#10<br><br>*c1' region:*<br><br>800 MHz, ns: 48, sw(f3): 9 ppm, sw(f2): 12 ppm, TD(f3): 1024, TD(f2): 336, aq(f3): 71 ms, o1p(^1H): 4.7 ppm, o2p(^{13}C): 90 ppm, o3p(^{15}N): 153 ppm, d1: 5 s, presaturation delay: 3 s, INEPT transfer time: 3.0 ms, 3 d 1 h, (308 K), sample#4.5<br><br>*NOE/noNOE experiments were recorded as pseudo 3D. An off resonant pulse at -1000 ppm was used for temperature compensation in the reference experiment.*
**3D *fw*-HCC-TOCSY-CCH-E.COSY**<br>(26, 27)	700 MHz, ns: 24, sw(f3): 10 ppm, sw(f2): 35 ppm, sw(f1): 4 ppm TD(f3): 1024, TD(f2): 72, TD(f1): 96, aq (f3): 70 ms, o1p (^1H): 4.7 ppm, o2p(^{13}C): 77 ppm, o3p(^{15}N): 4.7 ppm, d1: 1.1 s, 2d 10 h, sample#10
**2D Γ-HCCH**<br>(32, 33)	*Ref:*<br>700 MHz, ns: 64, sw(f2): 16 ppm, sw(f1): 35 ppm, TD(f2): 2048, TD(f1): 192, aq(f2): 90 ms, o1p(^1H): 4.7 ppm, o2p(^{13}C): 76.5 ppm, d1: 1.5 s, 6 h, sample#10<br><br>*Cross:*<br>700 MHz, ns: 512, sw(f2): 16 ppm, sw(f1): 35 ppm, TD(f2): 2048, TD(f1): 192, aq(f2): 90 ms, o1p(^1H): 4.7 ppm, o2p(^{13}C): 76.5 ppm, d1: 1.5 s, 2 d, sample#10
**3D *fw*-Γ-HCCH**<br>(32, 33)	*Ref:*<br>700 MHz, ns: 8, sw(f3): 8.5 ppm, sw(f2): 37 ppm, sw(f1): 4 ppm TD(f3): 1024, TD(f2): 80, TD(f1): 96, aq (f3): 85 ms, o1p (^1H): 4.7 ppm, o2p(^{13}C): 77 ppm, o3p(^{15}N): 4.7 ppm, d1: 1.0 s, 20 h, sample#10<br><br>*Cross:*<br>700 MHz, ns: 16, sw(f3): 8.5 ppm, sw(f2): 37 ppm, sw(f1): 4 ppm TD(f3): 1024, TD(f2): 80, TD(f1): 96, aq (f3): 85 ms, o1p (^1H): 4.7 ppm, o2p(^{13}C): 77 ppm, o3p(^{15}N): 4.7 ppm, d1: 1.0 s, 20 h, sample#10
**2D Γ-HCN**<br>(34)	*Ref:*<br>600 MHz, ns: 512, sw(f2): 10 ppm, sw(f1): 8 ppm, TD(f2): 2048, TD(f1): 72, aq(f2): 170 ms, o1p(^1H): 4.7 ppm, o2p(^{13}C): 89 ppm, d1: 1.4 s, 18 h, sample#10<br><br>*Cross:*<br>600 MHz, ns: 2048, sw(f2): 10 ppm, sw(f1): 8 ppm, TD(f2): 2048, TD(f1): 72, aq(f2): 170 ms, o1p(^1H): 4.7 ppm, o2p(^{13}C): 89 ppm, d1: 1.4 s, 3d 1h, sample#10
**T_1**<br>(30, 31, 35)	600 MHz, ns: 32, sw(f3): 14 ppm, sw(f2): 10 ppm, TD(f3): 1024, TD(f2): 64, aq (f3): 60 ms, o1p (^1H): 4.7 ppm, o2p(^{13}C): 137.5 ppm, d1: 2.6 s, 1d 4 h, sample#10<br><br>Delays: 20 ms, 70 ms, 150 ms (3x), 250 ms, 500 ms, 800 ms, 1200 ms (3x), 1500 ms
**$T_{1\rho}$**<br>(30, 36–38)	600 MHz, ns: 104, sw(f3): 16 ppm, sw(f2): 29 ppm, TD(f3): 1024, TD(f2): 128, aq (f3): 100 ms, o1p (^1H): 4.7 ppm, o2p(^{13}C): 137.5 ppm, d1: 1 s, 1d 12 h, sample#10<br><br>Delays: 10 ms, 12 ms, 17 ms (3x), 25 ms, 31 ms, 42 ms, 65 ms (3x), 79 ms

Supplementary Table 7. Dihedral angle restraints of the NMR solution structure of s2m Delta_short RNA (weighting 1 for all nucleobases). Residues G-2, G-1, C+2 and C+1 had to be sequentially numbered in ARIA structure calculations. C+2 corresponds to C761, C+1 to C760, G-1 to G738 and G-2 to G737.

Residue	seq	v0	v1	v2	v3	v4	χ	ß	ε	α	ζ	γ
**G**	737											
**G**	738	3 ± 22	-25 ± 20	36 ± 14	-35 ± 15	20 ± 23	200 ± 40	175 ± 45	210 ± 40	295 ± 35	290 ± 45	55 ± 35
**C**	739	3 ± 23	-25 ± 20	36 ± 14	-35 ± 15	20 ± 23	200 ± 40	175 ± 45	210 ± 40	295 ± 35	290 ± 45	55 ± 35
**A**	740	3 ± 24	-25 ± 20	36 ± 14	-35 ± 15	20 ± 23	200 ± 40	175 ± 45	210 ± 40	295 ± 35	290 ± 45	55 ± 35
**C**	741	3 ± 25	-25 ± 20	36 ± 14	-35 ± 15	20 ± 23	200 ± 40	175 ± 45	210 ± 40	295 ± 35	290 ± 45	55 ± 35
**U**	742	3 ± 26	-25 ± 20	36 ± 14	-35 ± 15	20 ± 23	200 ± 40	175 ± 45	210 ± 40	295 ± 35	290 ± 45	55 ± 35
**C**	743	3 ± 27	-25 ± 20	36 ± 14	-35 ± 15	20 ± 23	200 ± 40	175 ± 45	210 ± 40	295 ± 35	290 ± 45	55 ± 35
**G**	744											
**G**	745											
**A**	746											
**G**	747											
**U**	748											
**A**	749											
**C**	750											
**G**	751											
**A**	752											
**U**	753											
**C**	754											
**G**	755	3 ± 22	-25 ± 20	36 ± 14	-35 ± 15	20 ± 23	200 ± 40	175 ± 45	210 ± 40	295 ± 35	290 ± 45	55 ± 35
**A**	756	3 ± 23	-25 ± 20	36 ± 14	-35 ± 15	20 ± 23	200 ± 40	175 ± 45	210 ± 40	295 ± 35	290 ± 45	55 ± 35
**G**	757	3 ± 24	-25 ± 20	36 ± 14	-35 ± 15	20 ± 23	200 ± 40	175 ± 45	210 ± 40	295 ± 35	290 ± 45	55 ± 35
**U**	758	3 ± 25	-25 ± 20	36 ± 14	-35 ± 15	20 ± 23	200 ± 40	175 ± 45	210 ± 40	295 ± 35	290 ± 45	55 ± 35
**G**	759	3 ± 26	-25 ± 20	36 ± 14	-35 ± 15	20 ± 23	200 ± 40	175 ± 45	210 ± 40	295 ± 35	290 ± 45	55 ± 35
**C**	760	3 ± 27	-25 ± 20	36 ± 14	-35 ± 15	20 ± 23	200 ± 40	175 ± 45	210 ± 40	295 ± 35	290 ± 45	55 ± 35
**C**	761											

Supplementary Table 8. Hydrogen bond restraints of the NMR solution structure of s2m Delta_short RNA (CNS format). Residues G-2, G-1, C+2 and C+1 had to be sequentially numbered in ARIA structure calculations. C+2 corresponds to C761, C+1 to C760, G-1 to G738 and G-2 to G737. Listed are the distances and errors.

Base pair	Assignment Hydrogen bonds	Base pair	Assignment Hydrogen bonds
**G-2(737)-C+2(761)**	assign (resid 761 and name N3)(resid 737 and name N1) 2.90 0.6 0.5	**U742-A756**	assign (resid 756 and name N1)(resid 742 and name N3) 2.90 0.5 0.4
	assign (resid 761 and name N3)(resid 737 and name H1) 1.90 0.5 0.5		assign (resid 756 and name N1)(resid 742 and name H3) 1.90 0.4 0.4
	assign (resid 761 and name N4)(resid 737 and name O6) 2.90 0.6 0.5		assign (resid 756 and name N6)(resid 742 and name O4) 2.90 0.5 0.4
	assign (resid 761 and name H42)(resid 737 and name O6) 1.90 0.5 0.5		assign (resid 756 and name H62)(resid 742 and name O4) 1.90 0.4 0.4
	assign (resid 761 and name O2)(resid 737 and name N2) 2.90 0.6 0.5		
	assign (resid 761 and name O2)(resid 737 and name H22) 1.90 0.5 0.5		
**G-1(738)-C+1(760)**	assign (resid 760 and name N3)(resid 738 and name N1) 2.90 0.5 0.4	**C743-G755**	assign (resid 743 and name N3)(resid 755 and name N1) 2.90 0.5 0.4
	assign (resid 760 and name N3)(resid 738 and name H1) 1.90 0.4 0.4		assign (resid 743 and name N3)(resid 755 and name H1) 1.90 0.4 0.4
	assign (resid 760 and name N4)(resid 738 and name O6) 2.90 0.5 0.4		assign (resid 743 and name N4)(resid 755 and name O6) 2.90 0.5 0.4
	assign (resid 760 and name H42)(resid 738 and name O6) 1.90 0.4 0.4		assign (resid 743 and name H42)(resid 755 and name O6) 1.90 0.4 0.4
	assign (resid 760 and name O2)(resid 738 and name N2) 2.90 0.5 0.4		assign (resid 743 and name O2)(resid 755 and name N2) 2.90 0.5 0.4
	assign (resid 760 and name O2)(resid 738 and name H22) 1.90 0.4 0.4		assign (resid 743 and name O2)(resid 755 and name H22) 1.90 0.4 0.4
**C739-G759**	assign (resid 739 and name N3)(resid 759 and name N1) 2.90 0.5 0.4	**G744-C754**	assign (resid 754 and name N3)(resid 744 and name N1) 2.90 0.6 0.5
	assign (resid 739 and name N3)(resid 759 and name H1) 1.90 0.4 0.4		assign (resid 754 and name N3)(resid 744 and name H1) 1.90 0.5 0.5
	assign (resid 739 and name N4)(resid 759 and name O6) 2.90 0.5 0.4		assign (resid 754 and name N4)(resid 744 and name O6) 2.90 0.6 0.5
	assign (resid 739 and name H42)(resid 759 and name O6) 1.90 0.4 0.4		assign (resid 754 and name H42)(resid 744 and name O6) 1.90 0.5 0.5
	assign (resid 739 and name O2)(resid 759 and name N2) 2.90 0.5 0.4		assign (resid 754 and name O2)(resid 744 and name N2) 2.90 0.6 0.5
	assign (resid 739 and name O2)(resid 759 and name H22) 1.90 0.4 0.4		assign (resid 754 and name O2)(resid 744 and name H22) 1.90 0.5 0.5
**A740-U758**	assign (resid 740 and name N1)(resid 758 and name N3) 2.90 0.5 0.4	**G745-U753**	assign (resid 753 and name N3)(resid 745 and name O6) 2.90 0.6 0.5
	assign (resid 740 and name N1)(resid 758 and name H3) 1.90 0.4 0.4		assign (resid 753 and name H3)(resid 745 and name O6) 1.90 0.5 0.5
	assign (resid 740 and name N6)(resid 758 and name O4) 2.90 0.5 0.4		assign (resid 753 and name O2)(resid 745 and name N1) 2.90 0.6 0.5
	assign (resid 740 and name H62)(resid 758 and name O4) 1.90 0.4 0.4		assign (resid 753 and name O2)(resid 745 and name H1) 1.90 0.5 0.5
**C741-G757**	assign (resid 741 and name N3)(resid 757 and name N1) 2.90 0.5 0.4		
	assign (resid 741 and name N3)(resid 757 and name H1) 1.90 0.4 0.4		
	assign (resid 741 and name N4)(resid 757 and name O6) 2.90 0.5 0.4		
	assign (resid 741 and name H42)(resid 757 and name O6) 1.90 0.4 0.4		
	assign (resid 741 and name O2)(resid 757 and name N2) 2.90 0.5 0.4		
	assign (resid 741 and name O2)(resid 757 and name H22) 1.90 0.4 0.4		

Supplementary Table 9. Planarity restraints of the NMR solution structure of s2m Delta_short RNA (CNS format). Residues G-2, G-1, C+2 and C+1 had to be sequentially numbered in ARIA structure calculations. C+2 corresponds to C761, C+1 to C760, G-1 to G738 and G-2 to G737. Listed are the distances and errors.

Base pair	selection	weight
**G-2(737)-C+2(761)**	((resid 737 and name N1) or (resid 737 and name N3) or (resid 737 and name C5) or (resid 761 and name N1) or (resid 761 and name N3) or (resid 761 and name C5))	10
**G-1(738)-C+1(760)**	((resid 738 and name N1) or (resid 738 and name N3) or (resid 738 and name C5) or (resid 760 and name N1) or (resid 760 and name N3) or (resid 760 and name C5))	10
**C739-G759**	((resid 739 and name N1) or (resid 739 and name N3) or (resid 739 and name C5) or (resid 759 and name N1) or (resid 759 and name N3) or (resid 759 and name C5))	25
**A740-U758**	((resid 740 and name N1) or (resid 740 and name N3) or (resid 740 and name C5) or (resid 758 and name N1) or (resid 758 and name N3) or (resid 758 and name C5))	25
**C741-G757**	((resid 741 and name N1) or (resid 741 and name N3) or (resid 741 and name C5) or (resid 757 and name N1) or (resid 757 and name N3) or (resid 757 and name C5))	25
**U742-A756**	((resid 742 and name N1) or (resid 742 and name N3) or (resid 742 and name C5) or (resid 756 and name N1) or (resid 756 and name N3) or (resid 756 and name C5))	25
**C743-G755**	((resid 743 and name N1) or (resid 743 and name N3) or (resid 743 and name C5) or (resid 755 and name N1) or (resid 755 and name N3) or (resid 755 and name C5))	10
**G744-C754**	((resid 744 and name N1) or (resid 744 and name N3) or (resid 744 and name C5) or (resid 754 and name N1) or (resid 754 and name N3) or (resid 754 and name C5))	10
**G745-U753**	((resid 745 and name N1) or (resid 745 and name N3) or (resid 745 and name C5) or (resid 753 and name N1) or (resid 753 and name N3) or (resid 753 and name C5))	10

Supplementary Table 10. SAS sample details, data collection, analysis, and 3D modelling details for s2m Delta_short RNA in solution**.**

**(a) Sample details**		
**Organism**	severe acute respiratory syndrome coronavirus 2	severe acute respiratory syndrome coronavirus 2
**Source (Catalogue No. or reference)**	Rfam: RF00164	Rfam: RF00164
**Scattering particle composition**		
**RNA**	s2m Delta_short	s2m Delta_short
**Sample environment/configuration**		
**Solvent composition**	50 mM BisTris, 25 mM NaCl, pH 6.2	50 mM BisTris, 25 mM NaCl, pH 6.2
**Sample temperature (°C)**	25	25
**Batch measurements**		
**Sample concentration, mg/ml**	1.5	2.5
**(b) SAS data collection**		
**Data acquisition/reduction software**	Beamline Meta Server	
**Source/instrument description or reference**	EMBL P12 BioSAXs beamline at the PETRA III storage ring (DESY, Hamburg, Germany) using a Pilatus 6 M 2D photon	
**Measured q-range ($q_{min} – q_{max}$, nm^{-1})**	0.0223 to 7.31	
**Method for scaling intensities**	arbitrary units	
**Exposure time, number of exposures**	3.135 s (33 × 95 ms)	
**Additional relevant details**	sample-detector distance of 3.0 m at wavelength of λ = 0.124 nm	
**(c) SAS-derived structural parameters**		
**Methods/Software**	BioXTAS RAW	
**Guinier Analysis**		
**I(0) ± σ (a.u)**	0.04 ± 2.69e-5	0.04 ± 7.85e-5
**R_g ± σ (nm)**	1.49 ± 2.22e-3	1.39 ± 0.05
**min < qR_g < max limit (data point range)**	0.0223 to 0.8697	0.0335 to 0.9338
**Linear fit assessment (r^2)**	0.775	0.902
**PDDF/P(r) analysis**		
**I(0) ± σ (a.u.)**	0.04 +/- 2.85e-5	0.04 +/- 1.79e-5
**R_g ± σ (nm)**	1.55 +/- 1.1e-3	1.49 +/- 6.06e-4
**d_{max} (nm)**	5.0	4.0
**q-range (nm^{-1})**	0.0223 to 7.3144e+0	0.0335 to 7.3144e+0
**P(r) fit assessment (χ^2)**	2.0	9.6
**(d) Scattering particle size**		
**Methods/Software**	BioXTAS RAW	
**Volume estimates**		
**Porod volume, V_p (Å^3)**	8.93e+3	8.18e+3
**Molecular weight (M) estimates (kDa)**		
**From chemical composition**	8.272	8.272
**From SAS (Bayesian interference, range with % confidence)**	6.7 to 9.1 (100%)	6.7 to 9.1 (99.1%)
**Bayes, Probability**	8.5 (56.6%)	7.3 (99.1%)
**From SAS-independent measure**	n.a.	n.a.

**(e) Modelling**		
**Data**	s2m Delta_short<br>1.5 mg/mL	s2m Delta_short<br>1.5 mg/mL cropped data
**Software**	Dummy atom (DAMMIN) (ATSAS)	DENSS
**q-range for fit ($q_{min} – q_{max}$, nm^{-1})**	0.0482-0.5139	0.0323 to 0.7314
**Symmetry/anisotropy assumptions**	P1 symmetry	False
**Number of individual model reconstructions**	10	20
**Resolution**	1.043	21.1 +/- 4.7
**(f) Data and model deposition**		
	Sample 1	Sample 2
**SASBDB IDs**	SASDUD9	SASDXB3

Supplementary Table 11. χ² values of s2m Delta_short from back calculated scattering curves. 5MP3OH: ARIA structure ensemble models without modifications. 5TP3cP: Ensemble models from ARIA structure calculation were modified to each contain a 5′ triphosphate and 3′ cyclic phosphate. SAXS scattering curves at two different concentrations (1.5 mg/mL and 2.5 mg/mL). The scattering curves were cropped in the first 50 points (cropped). Furthermore, the low angle data (first 500 points) measured at low concentration was merged with the high angle data at higher concentration (Merge). Top 10 χ² values from each condition are marked in green.

	5MP3OH					5TP3cP				
	uncropped		cropped			uncropped		cropped		
**model**	1.5 mg/mL	2.5 mg/mL	1.5 mg/mL	2.5 mg/mL	Merge	1.5 mg/mL	2.5 mg/mL	1.5 mg/mL	2.5 mg/mL	Merge
**1**	9.726	18.12	7.458	16.98	11.40	5.583	17.28	4.628	15.05	7.940
**2**	8.138	20.90	7.745	18.86	12.65	6.154	19.18	6.184	16.97	9.220
**3**	2.827	18.03	2.745	15.36	3.207	2.814	20.98	2.701	18.63	3.044
**4**	2.902	18.21	2.871	15.52	3.367	3.021	18.91	2.829	16.59	3.253
**5**	5.856	17.59	6.223	15.73	9.154	5.070	17.18	4.345	14.93	6.915
**6**	6.766	17.39	6.930	16.93	11.97	4.705	15.82	3.366	13.56	6.408
**7**	13.52	28.10	13.64	28.12	24.62	13.80	28.11	11.77	26.09	22.31
**8**	7.211	18.62	7.668	16.77	11.51	5.516	16.78	4.489	14.54	7.885
**9**	7.309	20.79	7.270	18.58	11.64	6.332	19.88	5.949	17.67	9.612
**10**	7.720	19.37	8.381	17.89	12.4	6.940	18.76	5.653	16.57	10.18
**11**	10.25	59.19	10.54	22.48	18.62	10.44	22.23	8.494	20.12	15.88
**12**	13.39	28.07	14.37	27.13	23.32	12.24	25.74	10.94	23.68	19.38
**13**	11.09	24.15	11.14	22.53	18.32	9.414	21.77	8.437	19.64	14.88
**14**	13.89	27.62	14.53	27.92	24.69	13.64	27.11	11.47	25.08	21.70
**15**	8.069	19.73	8.515	19.66	15.02	6.456	17.87	4.432	15.66	9.317
**16**	13.75	28.87	14.12	28.89	24.54	14.33	31.43	14.03	29.46	25.00
**17**	8.140	19.91	8.473	19.57	14.45	6.662	18.81	5.293	16.61	9.994
**18**	11.08	25.97	11.28	24.69	19.59	10.02	23.72	7.71	21.61	15.86
**19**	8.497	19.22	7.654	18.41	13.67	7.622	18.56	5.635	16.37	11.00
**20**	17.23	34.52	18.08	34.56	31.18	15.58	31.77	12.96	29.81	26.00
**All**	8.344	20.49	8.292	19.45	14.09	7.302	19.33	6.077	17.14	10.90
**10 best**	5.845	17.32	5.676	15.94	8.976	4.673	17.29	3.889	15.05	6.343

Supplementary Table 12. ^{13}C Assignment of s2m Delta_short at 308 K. The completeness of the assignment is given in the last row. Units in ppm.

	C1′	C2′	C3′	C4′	C5′	C8	C5	C4	C6	C2
**C+2**	92.01	85.23	77.89	85.51	65.68		99.15	168.2	141.8	-
**C+1**	94.39	75.66	72.19	82.13	64.47		97.02	167.6	141.2	-
**G759**	92.92	75.39	72.87	82.12	65.74	136.2	118.4	151.3	160.7	155.7
**U758**	93.46	75.37	72.41	82.12	64.50		103.0	168.3	141.2	151.9
**G757**	92.92	75.33	72.71	82.12	65.95	135.8	118.6	151.2	160.9	156.0
**A756**	92.89	75.69	72.82	82.04	65.06	139.4	120.5	148.0	157.2	153.3
**G755**	92.88	75.30	73.44	82.22	66.46	136.4	118.1	-	160.3	154.7
**C754**	93.47	75.43	73.06	81.88	65.58		98.18	167.9	142.2	-
**U753**	92.45	75.36	74.79	83.76	66.38		104.8	-	142.6	-
**A752**	90.94	76.12	76.87	84.74	67.70	141.5	120.4	150.0	156.9	155.1
**G751**	90.37	75.52	76.95	84.95	68.09	140.2	118.2	-	-	-
**C750**	91.75	75.81	76.42	84.44	67.45		98.84	-	143.7	-
**A749**	90.13	76.34	77.13	85.32	67.84	142.0	120.3	150.5	157.0	155.1
**U748**	90.22	75.65	77.12	85.15	67.66		105.3	-	143.7	-
**G747**	90.21	74.96	76.59	84.80	67.07	142.1	117.6	-	-	-
**A746**	92.14	75.96	74.05	83.41	65.73	140.0	120.1	148.8	156.8	155.1
**G745**	92.69	73.84	75.56	82.99	65.88	137.3	117.8	151.4	-	154.4
**G744**	92.57	75.60	73.38	82.28	66.05	136.4	-	-	160.5	154.8
**C743**	93.72	75.53	72.60	81.81	64.84		97.81	167.8	141.4	-
**U742**	93.83	75.31	72.32	82.13	64.42		103.2	168.6	142.1	152.1
**C741**	93.90	75.59	72.48	81.96	64.88		97.21	167.4	140.8	-
**A740**	93.06	75.64	73.03	81.99	65.34	139.4	120.5	148.8	157.4	153.3
**C739**	93.82	75.43	72.34	81.88	64.52		97.44	167.9	140.8	-
**G-1**	92.98	75.39	73.03	82.33	66.27	136.8	118.3	151.5	161.0	156.3
**G-2**	91.24	74.93	-	83.41	67.34	139.0	118.4	152.3	-	155.6
**Total**	**100%**	**100%**	**96%**	**100%**	**100%**	**100%**	**96%**	**72%**	**84%**	**56%**

Supplementary Table 13. ^{1}H Assignment of s2m Delta_short at 308 K. The completeness of the assignment is given in the last row. Units in ppm.

	**H1′**	**H2′**	**H3′**	**H4′**	**H5′**	**H5′′**	**H8**	**H2**	**H6**	**H5**	**H1**	**H3**
**C+2**	6.104	4.625	4.938	4.408	4.500	4.108			7.473	5.536		
**C+1**	5.481	4.176	4.427	4.378	4.541	4.067			7.646	5.243		
**G759**	5.808	4.530	4.545	4.512	4.496	4.140	7.691				12.57	
**U758**	5.541	4.610	4.540	4.419	4.533	4.082			7.648	5.031		13.62
**G757**	5.554	4.385	4.532	4.416	4.424	4.034	7.058				13.36	
**A756**	5.913	4.699	4.632	4.496	4.556	4.136	7.735	7.420				
**G755**	5.614	4.610	4.494	4.434	4.434	4.140	7.500				11.86	
**C754**	5.548	4.534	4.547	4.419	4.458	4.165			7.908	5.767		
**U753**	5.632	4.297	4.569	4.485	4.385	4.162			7.773	5.658		-
**A752**	5.946	4.748	4.755	4.512	4.339	4.244	8.267	8.056				
**G751**	5.774	4.766	4.823	4.480	4.215	4.215	7.913				-	
**C750**	5.703	4.218	4.573	4.271	4.022	4.023			7.556	5.800		
**A749**	5.853	4.683	4.672	4.407	4.153	4.058	8.174	8.009				
**U748**	5.795	4.261	4.554	4.261	4.025	4.026			7.704	5.719		-
**G747**	5.524	4.638	4.643	4.401	4.297	4.094	7.584				-	
**A746**	5.797	4.444	4.669	4.396	4.379	4.103	7.880	7.999				
**G745**	5.569	4.508	4.490	4.393	4.394	4.072	7.213				-	
**G744**	5.648	4.519	4.526	4.493	4.449	4.101	7.504				12.19	
**C743**	5.562	4.486	4.530	4.420	4.517	4.106			7.802	5.620		
**U742**	5.544	4.470	4.509	4.419	4.553	4.082			7.857	5.330		14.02
**C741**	5.373	4.230	4.345	4.415	4.513	4.069			7.482	5.248		
**A740**	5.957	4.590	4.637	4.507	4.564	4.170	8.026	7.412				
**C739**	5.538	4.534	4.561	4.419	4.587	4.137			7.695	5.293		
**G-1**	5.899	4.542	4.361	4.548	4.510	4.282	7.606				13.27	
**G-2**	5.814	4.928	-	4.557	4.415	4.280	8.121				-	
**Total**	**100%**	**100%**	**96%**	**100%**	**100%**	**100%**	**100%**	**100%**	**100%**	**100%**	**55%**	**50%**

Supplementary Table 14. ^{15}N Assignment of s2m Delta_short at 283 K, 298 K and 308 K. The completeness of the assignment is given in the last row. Units in ppm.

	N1			N3			N7	N9		N4/N6	
**Temp.**	**283**	**298**	**308**	**283**	**298**	**308**	**298**	**298**	**308**	**298**	**308**
**C+2**			150.3	196.0						99.21	99.05
**C+1**			151.4	198.0						99.18	98.97
**G759**	147.8	147.9	147.9	-	-	-	234.7	169.4	169.4		74.90
**U758**			145.7	162.2	162.2	162.1					
**G757**	148.1	148.2	148.3	-	-	-	234.9	168.9	169.0		74.40
**A756**	220.7	220.9			212.6		229.8	170.6	170.7		82.56
**G755**	145.9	146.1	146.2	-	-	-	235.0	168.7	168.9		73.85
**C754**			151.3	196.1						97.13	96.77
**U753**			144.8	-	-	-					
**A752**		227.4			216.7		231.1	169.4	169.0		80.19
**G751**	-	-	-	-	-	-	235.6	167.7	167.8	-	-
**C750**			151.3	-	-	-				-	-
**A749**		224.3			217.4		232.2	168.6	168.5		78.66
**U748**			143.7	-	-	-					
**G747**	-	-	-	-	-	-	234.5	166.0	166.5	-	-
**A746**		224.2			215.2		231.3	170.0	169.8		80.87
**G745**	143.6	144.2		-	-	-	233.5	168.8			72.51
**G744**	147.4	147.2	147.0	-	-	-	234.5	168.7	168.8		73.17
**C743**			150.9	197.2						97.21	97.19
**U742**			146.5	162.6	162.6	162.5					
**C741**			151.0	197.5						98.51	98.39
**A740**	222.2	222.4			213.2		230.5	170.7	170.7		83.88
**C739**			150.9	196.8						98.15	98.01
**G-1**	148.4	148.4	148.4	-	-	-	233.6	169.3	169.4		74.69
**G-2**	147.3	147.2		-	-	-	232.2	168.6			75.22
**Total**		**92%**			**52%**		**100%**	**100%**		**84%**	

Supplementary Table 15. Number of experimental restraints used in ARIA structure calculation.

(a) RNA	s2m Delta_short
Number of nucleotides	25
(b) NMR experimental restraints	
Total no. experimental restraints	571
Average no. of restraints per nucleotide	22.84
Average no. of NOE-derived restraints per nucleotide	15.28
(c) NOE-derived restraints	382
Intraresidue	226
Inter-residue	156
Sequential |i-j| = 1	144
Non-sequential |i-j| >1	12
(d) Dihedral restraints	132
(e) Hydrogen-bonding restraints	48
(f) Planarity restraints	9
(g) Structure analysis of 10-member ensemble	
χ^2 to SAXS data	3.889
All atom r.m.s.d. from mean structure, Å	3.31 ± 0.77
(h) Structure analysis best model	
χ^2 to SAXS data	2.701

Supplementary Table 16. R_g and D_{max} values from ARIA structure calculation ensemble models derived from CRYSOL. 5MP3OH: ARIA structure ensemble models without modifications. 5TP3cP: Ensemble models from ARIA structure calculation were modified to each contain a 5′ triphosphate and 3′ cyclic phosphate.

model	5MP3OH		5TP3cP	
	R_g [nm]	D_{max} [nm]	R_g [nm]	D_{max} [nm]
1	1.339	4.284	1.355	4.429
2	1.366	4.457	1.380	4.631
3	1.426	4.699	1.443	4.852
4	1.436	4.932	1.434	5.068
5	1.351	4.411	1.353	4.584
6	1.311	4.097	1.333	4.360
7	1.291	4.024	1.292	4.193
8	1.349	4.183	1.367	4.369
9	1.382	4.570	1.396	4.760
10	1.349	4.351	1.351	4.501
11	1.316	4.059	1.320	4.059
12	1.303	4.052	1.304	4.209
13	1.331	4.268	1.347	4.454
14	1.283	3.792	1.288	3.878
15	1.315	4.224	1.334	4.411
16	1.328	4.268	1.337	4.393
17	1.343	4.263	1.360	4.419
18	1.333	4.139	1.338	4.167
19	1.329	4.142	1.336	4.256
20	1.282	3.990	1.292	4.159
Mean all	1.338	4.260	1.348	4.408
Mean 10 best	1.355	4.359	1.367	4.525

Supplementary Table 17. ^3J (H1′H2′; H2′H3′; H3′H4′) of s2m Delta_short RNA from *fw*-HCC-TOCSY-CCH-E.COSY experiment (26, 27)**.** Values were determined using three different methods (visually, manually and ecosy2 python script (39). Errors are the standard deviation. For ^3J (H3′H4′) coupling in stem nucleotides an error of 1.5 Hz was set. (*) Percentage (x) given for P=18°. For P=162° (1-x%)

**Nuc.**	**^3J (H1′H2′) [Hz]**			**^3J (H3′H4′) [Hz]**			**Mean ^3J (H1′H2′) [Hz]**	**^3J (H2′H3′) Script**	**Mean ^3J (H3′H4′) [Hz]**	**P [°]**	**P [°] Two-state model 18°**
	script	manually	visually	script	manually	visually					
**G-2**											
**G-1**											
**C739**											
**A740**					11.90	10.89			11.40 ± 1.5	74.93	
**C741**		1.25	0.84		12.08	11.54	1.04 ± 0.21	4.34	11.81 ± 1.5	28.36	
**U742**											
**C743**											
**G744**	4.06	3.46	3.10	9.62	9.72	8.95	3.54 ± 0.40		9.43 ± 1.5	57.20	
**G745**		7.70	4.33				6.01 ± 1.69	7.20		82.51	
**A746**	2.57	2.81	3.05	6.84	7.28	7.99	2.81 ± 0.20		7.37 ± 0.47	53.57	78%*
**G747**	6.38	7.29	8.38	4.92	4.90	2.80	7.35 ± 0.82		4.21 ± 0.99	121.03	10-40%*
**U748**	8.55	7.85	7.84	3.42	3.84	3.72	8.08 ± 0.33		3.66 ± 0.18	127.14	0-30%*
**A749**	8.98	5.44	4.03	3.42	3.35	3.38	6.15 ± 2.08		3.38 ± 0.03	126.93	30%*
**C750**	2.14	0.91	1.58	5.77	4.79	2.07	1.54 ± 0.50		4.21 ± 1.56	310.14	40-95%*
**G751**	5.56	7.27	7.87	6.20	4.41	4.52	6.90 ± 0.98		5.04 ± 0.82	112.46	15-60%*
**A752**	6.63	6.10	5.93	7.69	7.44	4.23	6.22 ± 0.30		6.45 ± 1.58	96.90	25-70%*
**U753**	2.99	0.35	0.72	1.28	2.88	6.99	1.35 ± 1.17		3.72 ± 2.41	305.61	10-40%*
**C754**											
**G755**											
**A756**											
**G757**											
**U758**											
**G759**											
**C+1**		3.95	3.09		11.67	10.65	3.52 ± 0.43		11.16 ± 1.5	53.74	
**C+2**		3.79	2.86	8.12	6.53	4.68	3.32 ± 0.47		6.44 ± 1.5	58.06	

Supplementary Table 18. Dipole (^1H,^{13}C)-dipole (^1H,^{13}C)-CCR $\Gamma^{DD,DD}_{C1'H1'C2'H2'}$ and $\Gamma^{DD,DD}_{C3'H3'C4'H4'}$ of s2m Delta_short RNA from quantitative 2D Γ-HCCH and 3D *fw*-Γ-HCCH experiments (32)**.** Errors are the standard deviation between 2 experiments.

Nuc.	2D			3D		Mean	Mean	Ratio	P [°]	P [°] Two-state model 18°(X%)/162°(1-X%)
	$\Gamma^{DD.DD}_{C1'H1'C2'H2'}$	$\Gamma^{DD.DD}_{C2'H2'C1'H1'}$	$\Gamma^{DD.DD}_{C3'H3'C4'H4'}$	$\Gamma^{DD.DD}_{C1'H1'C2'H2'}$	$\Gamma^{DD.DD}_{C3'H3'C4'H4'}$	$\Gamma^{DD.DD}_{C1'H1'C2'H2'}$	$\Gamma^{DD.DD}_{C3'H3'C4'H4'}$			
**G-2**										
**G-1**				-13.40	9.77	-13.40	9.77	-1.37	319.9	
**C739**	-14.52					-14.52			0.65	
**A740**	-17.29			-10.91	27.04	-14.10 ± 3.19	27.04	-0.52	8.4	
**C741**	-13.63	-15.72		-10.28	21.50	-13.21 ± 2.24	21.50	-0.61	345.8	
**U742**	-11.91			-10.49		-11.20 ± 0.71			22.3	
**C743**				-9.84		-9.84			27.1	
**G744**	-8.80			-7.74	10.78	-8.27 ± 0.53	10.78	-0.77	327.8	
**G745**					15.42		15.42		330.9	
**A746**	-4.11	-3.98	22.78		19.62	-4.04 ± 0.07	21.20 ± 1.58	-0.19	39.6	
**G747**	10.42		5.80	16.92	6.51	13.67 ± 3.25	6.16 ± 0.35	2.22	-	25-50%/50-75%
**U748**	10.25	9.40	1.09	9.74	5.83	9.80 ± 0.35	3.46 ± 2.37	2.83	-	40%/60%
**A749**	5.73	5.22	7.51		4.54	5.48 ± 0.25	6.03 ± 1.49	0.91	-	51 %/49%
**C750**	7.09	3.67	2.78	11.17	5.94	7.31 ± 3.07	4.36 ± 1.58	1.68	-	46%/54%
**G751**	9.19		3.54	7.98	4.29	8.59 ± 0.60	3.92 ± 0.37	2.19	-	42%/58%
**A752**	6.81				6.34	6.81	6.34	1.07	-	50%/50%
**U753**		-8.55			7.04	-8.55	7.04	-1.21	318.8	
**C754**	-12.49			-12.79		-12.64 ± 0.15			15.8	
**G755**				-14.50	17.22	-14.50	17.22	-0.84	334.8	
**A756**	-13.61			-17.62	22.41	-15.62 ± 2.01	22.41	-0.70	348.3	
**G757**	-15.15			-12.93		-14.04 ± 1.11			6.2	
**U758**	-10.39			-10.37		-10.38 ± 0.01			25.3	
**G759**	-18.10			-14.16	15.30	-16.13 ± 1.97	15.30	-1.05	330.6	
**C+1**	-9.57			-7.77	12.97	-8.67 ± 0.90	12.97	-0.67	326.0	
**C+2**	-6.87				11.91	-6.87	11.91	-0.58	324.0	

Supplementary Table 19. T_1, $T_{1\rho}$ and hetNOE data used for fitting of spin specific global correlation time τ_C with Modelfree Relax software. The mean value τ_C = 3.898 ± 0.56 ns is the global correlation time of s2m Delta_short RNA.

Nuc.	*T_1 [s]*	*$T_{1\rho}$ [s]*	*HetNoe 600 MHz*	*HetNoe 800 MHz*	*R_1 [1/s]*	*$R_{1\rho}$ [1/s]*	*R_2 [1/s]*	*τ_c [ns]*	*Modelfree tm*
A740	0.4531 ± 0.0107	0.03951 ± 0.00317	1.124 ± 0.011	1.124 ± 0.01	2.21 ± 0.05	25.31 ± 2.03	37.62 ± 3.11	4.697	tm9
A746	0.3494 ± 0.00747	0.03365 ± 0.00459	1.176 ± 0.032	1.219 ± 0.02	2.86 ± 0.06	29.31 ± 2.03	45.84 ± 6.49	3.087	tm4
A752	0.363 ± 0.00677	0.06693 ± 0.0263	1.337 ± 0.043	1.298 ± 0.01	2.75 ± 0.05	14.94 ± 5.87	24.38 ± 10.4	3.94	tm2
A756	0.3761 ± 0.0114	0.0254 ± 0.00307	1.088 ± 0.019	1.118 ± 0.02	2.66 ± 0.08	48.69 ± 7.28	72.89 ± 11.1	3.826	tm9
G757	0.3524 ± 0.0114	0.02346 ± 0.000882	1.093 ± 0.018	1.147 ± 0.03	2.84 ± 0.13	42.63 ± 1.60	51.19 ± 1.95	3.555	tm9
G759	0.4308 ± 0.0156	0.03945 ± 0.000723	1.126 ± 0.011	1.155 ± 0.01	2.32 ± 0.08	25.35 ± 0.46	30.81 ± 0.58	4.283	tm4

Supplementary Table 20. χ angle values derived from Γ-HCN. S^2 value for stem nucleotides was set to 1 and for loop nucleotides 0.9.

	**nucleotide**	**CCR theoretical [Hz]**	**CCR exp [Hz]**	**χ [°]**	**difference**
**Purines**	**A740**	-3.33	-3.33	195.86	5.47E-04
	**A749**	3.36	3.36	203.43	1.37E-05
	**G755**	-14.46	-14.46	181.58	6.21E-05
	**G751**	1.02	1.017	200.61	4.86E-04
**Pyrimidines**	**C741**	-7.94	-7.940	201.07	-3.24678E-05

REFERENCES

1. Lee,W., Tonelli,M. and Markley,J.L. (2015) NMRFAM-SPARKY: enhanced software for biomolecular NMR spectroscopy. *Bioinformatics*, **31**, 1325–1327.

2. Matzel,T., Wirtz Martin,M., Herr,A., Wacker,A., Richter,C., Sreeramulu,S. and Schwalbe,H. (2024) NMR characterization and ligand binding site of the stem loop 2 motif (s2m) from the Delta variant of SARS-CoV-2. *RNA*, 10.1261/rna.079902.123.

3. Sklenar,V. (1995) Suppression of Radiation Damping in Multidimensional NMR Experiments Using Magnetic Field Gradients. *J Magn Reson A*, **114**, 132–135.

4. Sklenář,V. and Bax,A. (1987) Spin-echo water suppression for the generation of pure-phase two-dimensional NMR spectra. *Journal of Magnetic Resonance (1969)*, **74**, 469–479.

5. Schulte-Herbrüggen,T. and Sorensen,O.W. (2000) Clean TROSY: compensation for relaxation-induced artifacts. *J Magn Reson*, **144**, 123–128.

6. Favier,A. and Brutscher,B. (2011) Recovering lost magnetization: polarization enhancement in biomolecular NMR. *J Biomol NMR*, **49**, 9–15.

7. Solyom,Z., Schwarten,M., Geist,L., Konrat,R., Willbold,D. and Brutscher,B. (2013) BEST-TROSY experiments for time-efficient sequential resonance assignment of large disordered proteins. *J Biomol NMR*, **55**, 311–321.

8. Lescop,E., Schanda,P. and Brutscher,B. (2007) A set of BEST triple-resonance experiments for time-optimized protein resonance assignment. *J Magn Reson*, **187**, 163–169.

9. Lescop,E., Kern,T. and Brutscher,B. (2010) Guidelines for the use of band-selective radiofrequency pulses in hetero-nuclear NMR: Example of longitudinal-relaxation-enhanced BEST-type 1H–15N correlation experiments. *J Magn Reson*, **203**, 190–198.

10. Dingley,A.J. and Grzesiek,S. (1998) Direct Observation of Hydrogen Bonds in Nucleic Acid Base Pairs by Internucleotide 2JNN Couplings. *J Am Chem Soc*, **120**, 8293–8297.

11. Bodenhausen,G. and Ruben,D.J. (1980) Natural abundance nitrogen-15 NMR by enhanced heteronuclear spectroscopy. *Chem Phys Lett*, **69**, 185–189.

12. Vuister,G.W. and Bax,A. (1992) Resolution enhancement and spectral editing of uniformly 13C-enriched proteins by homonuclear broadband 13C decoupling. *Journal of Magnetic Resonance (1969)*, **98**, 428–435.

13. Mori,S., Abeygunawardana,C., Johnson,M.O. and van Zijl,P.C. (1995) Improved sensitivity of HSQC spectra of exchanging protons at short interscan delays using a new fast HSQC (FHSQC) detection scheme that avoids water saturation. *J Magn Reson B*, **108**, 94–98.

14. Mulder,F.A., Spronk,C.A., Slijper,M., Kaptein,R. and Boelens,R. (1996) Improved HSQC experiments for the observation of exchange broadened signals. *J Biomol NMR*, **8**, 223–228.

15. Mueller,L., Legault,P. and Pardi,A. (1995) Improved RNA Structure Determination by Detection of NOE Contacts to Exchange-Broadened Amino Protons. *J Am Chem Soc*, **117**, 11043–11048.

16. Sklenár,V., Peterson,R.D., Rejante,M.R. and Feigon,J. (1994) Correlation of nucleotide base and sugar protons in a 15N-labeled HIV-1 RNA oligonucleotide by 1H-15N HSQC experiments. *J Biomol NMR*, **4**, 117–122.

17. Hwang,T.L. and Shaka,A.J. (1995) Water Suppression That Works. Excitation Sculpting Using Arbitrary Wave-Forms and Pulsed-Field Gradients. *J Magn Reson A*, **112**, 275–279.

18. Sklenár,V., Peterson,R.D., Rejante,M.R. and Feigon,J. (1993) Two- and three-dimensional HCN experiments for correlating base and sugar resonances in 15N,13C-labeled RNA oligonucleotides. *J Biomol NMR*, **3**, 721–727.

19. Piotto,M., Saudek,V. and Sklenár,V. (1992) Gradient-tailored excitation for single-quantum NMR spectroscopy of aqueous solutions. *J Biomol NMR*, **2**, 661–665.

20. Sklenár,V., Dieckmann,T., Butcher,S.E. and Feigon,J. (1996) Through-bond correlation of imino and aromatic resonances in 13C-, 15N-labeled RNA via heteronuclear TOCSY. *J Biomol NMR*, **7**, 83–87.

21. Simon,B., Zanier,K. and Sattler,M. (2001) A TROSY relayed HCCH-COSY experiment for correlating adenine H2/H8 resonances in uniformly 13C-labeled RNA molecules. *J Biomol NMR*, **20**, 173–176.

22. Zhang,O., Kay,L.E., Olivier,J.P. and Forman-Kay,J.D. (1994) Backbone 1H and 15N resonance assignments of the N-terminal SH3 domain of drk in folded and unfolded states using enhanced-sensitivity pulsed field gradient NMR techniques. *J Biomol NMR*, **4**, 845–858.

23. Stanek,J., Podbevšek,P., Koźmiński,W., Plavec,J. and Cevec,M. (2013) 4D Non-uniformly sampled C,C-NOESY experiment for sequential assignment of 13C, 15N-labeled RNAs. *J Biomol NMR*, **57**, 1–9.

24. Kay,L.E., Xu,G.Y., Singer,A.U., Muhandiram,D.R. and Formankay,J.D. (1993) A Gradient-Enhanced HCCH-TOCSY Experiment for Recording Side-Chain 1H and 13C Correlations in H2O Samples of Proteins. *J Magn Reson B*, **101**, 333–337.

25. Marino,J.P., Schwalbe,H., Glaser,S.J. and Griesinger,C. (1996) Determination of g and Stereospecific Assignment of H5 Protons by Measurement of 2J and 3J Coupling Constants in Uniformly 13 C Labeled RNA. *J Am Chem Soc*, **118**, 4388–4395.

26. Schwalbe,H., Marino,J.P., Glaser,S.J., Griesinger,C. and Marino,J.P. (1995) Measurement of H,H-Coupling Constants Associated with ν1, ν2, and ν3 in Uniformly 13C-Labeled RNA by HCC-TOCSY-CCH-E.COSY. *J Am Chem Soc*, **117**, 7251–7252.

27. Glaser,S.J., Schwalbe,H., Marino,J.P. and Griesinger,C. (1996) Directed TOCSY, a method for selection of directed correlations by optimal combinations of isotropic and longitudinal mixing. *J Magn Reson B*, **112**, 160–180.

28. Schnieders,R., Wolter,A.C., Richter,C., Wöhnert,J., Schwalbe,H. and Fürtig,B. (2019) Novel 13 C-detected NMR Experiments for the Precise Detection of RNA Structure. *Angew Chem Int Ed Engl*, **58**, 9140–9144.

29. Fürtig,B., Schnieders,R., Richter,C., Zetzsche,H., Keyhani,S., Helmling,C., Kovacs,H. and Schwalbe,H. (2016) Direct 13C-detected NMR experiments for mapping and characterization of hydrogen bonds in RNA. *J Biomol NMR*, **64**, 207–221.

30. Duchardt,E. and Schwalbe,H. (2005) Residue specific ribose and nucleobase dynamics of the cUUCGg RNA tetraloop motif by MNMR 13C relaxation. *J Biomol NMR*, **32**, 295–308.

31. Farrow,N.A., Muhandiram,R., Singer,A.U., Pascal,S.M., Kay,C.M., Gish,G., Shoelson,S.E., Pawson,T., Forman-Kay,J.D. and Kay,L.E. (1994) Backbone dynamics of a free and phosphopeptide-complexed Src homology 2 domain studied by 15N NMR relaxation. *Biochemistry*, **33**, 5984–6003.

32. Felli,I.C., Richter,C., Griesinger,C. and Schwalbe,H. (1999) Determination of RNA sugar pucker mode from cross-correlated relaxation in solution NMR spectroscopy [2]. *J Am Chem Soc*, **121**, 1956–1957.

33. Richter,C., Griesinger,C., Felli,I., Cole,P.T., Varani,G. and Schwalbe,H. (1999) Determination of sugar conformation in large RNA oligonucleotides from analysis of dipole-dipole cross correlated relaxation by solution NMR spectroscopy. *J Biomol NMR*, **15**, 241–250.

34. Rinnenthal,J., Richter,C., Ferner,J., Duchardt,E. and Schwalbe,H. (2007) Quantitative Γ-HCNCH: Determination of the glycosidic torsion angle χ in RNA oligonucleotides from the analysis of CH dipolar cross-correlated relaxation by solution NMR spectroscopy. *J Biomol NMR*, **39**, 17–29.

35. Kay,L.E., Nicholson,L.K., Delaglio,F., Bax,A. and Torchia,D.A. (1992) Pulse sequences for removal of the effects of cross correlation between dipolar and chemical-shift anisotropy relaxation mechanisms on the measurement of heteronuclear T1 and T2 values in proteins. *Journal of Magnetic Resonance (1969)*, **97**, 359–375.

36. Dayie,K.T. and Wagner,G. (1994) Relaxation-Rate Measurements for 15N−1H Groups with Pulsed-Field Gradients and Preservation of Coherence Pathways. *J Magn Reson A*, **111**, 121–126.

37. Mulder,F.A.A., De Graaf,R.A., Kaptein,R. and Boelens,R. (1998) An Off-resonance Rotating Frame Relaxation Experiment for the Investigation of Macromolecular Dynamics Using Adiabatic Rotations. *Journal of Magnetic Resonance*, **131**, 351–357.

38. Korzhnev,D.M., Skrynnikov,N.R., Millet,O., Torchia,D.A. and Kay,L.E. (2002) An NMR experiment for the accurate measurement of heteronuclear spin-lock relaxation rates. *J Am Chem Soc*, **124**, 10743–10753.

39. Oxenfarth,A., Kümmerer,F., Bottaro,S., Schnieders,R., Pinter,G., Jonker,H.R.A., Fürtig,B., Richter,C., Blackledge,M., Lindorff-Larsen,K., *et al.* (2023) Integrated NMR/Molecular Dynamics Determination of the Ensemble Conformation of a Thermodynamically Stable CUUG RNA Tetraloop. *J Am Chem Soc*, **145**, 16557–16572.
